# A review on gold nanoparticles derived from plants and their antimicrobial applications

**DOI:** 10.1186/s11671-026-04465-1

**Published:** 2026-02-23

**Authors:** Rafaella Resende Marques, Rachel Ann Hauser-Davis, Enrico Mendes Saggioro

**Affiliations:** 1 Programa de Pós-Graduação em Saúde Pública e Meio Ambiente, Escola Nacional de Saúde Pública, Fundação Oswaldo Cruz, Rio de Janeiro, Brasil; 2 Laboratório de Avaliação e Promoção da Saúde Ambiental (LAPSA), Instituto Oswaldo Cruz (Fiocruz), Av. Brasil, 4.365, Manguinhos, Rio de Janeiro, RJ 21040-360 Brazil; 3 Environmental Health Evaluation and Promotion Laboratory, Oswaldo Cruz Institute, Oswaldo Cruz Foundation, 4365 Avenida Brasil, Manguinhos, Rio de Janeiro, RJ Brazil

**Keywords:** Antimicrobial activity, Green synthesis, Plant extracts, Gold nanoparticles, Sustainability

## Abstract

Gold nanoparticles (AuNPs) have attracted significant attention due to their broad range of applications in the health and environmental fields and the fact that gold is an inert metal with low physiological toxicity, making it a favorable candidate for biomedical use. In line with the growing interest in green nanotechnology, plant-based nanoparticle synthesis has emerged as a sustainable, low-cost, and environmentally friendly alternative to conventional methods. Among these, AuNPs synthesized using plant extracts have been extensively studied, particularly concerning their antimicrobial properties. In this sense, this study aimed to perform a bibliometric review of the antimicrobial potential of plant-based AuNPs focusing on articles published between 2014 and 2025. Searches were conducted at the Web of Science, PubMed, and Scopus databases, totaling 190 eligible studies. India is the leading country in terms of number of publications (36.84%), with leaves as the most employed plant part (42.6%). The reported AuNPs synthesized from various plant parts, including seeds, fruits, as well as leaves, exhibited significant antimicrobial activities. Although some AuNPs, such as those synthesized from seeds, were more effective against Gram-positive bacteria, others, such as those from fruits, showed greater activity against Gram-negative strains, and several leaf-based ones demonstrated broad-spectrum efficacy against both groups. These antimicrobial performance variations are primarily attributed to the presence of specific organic compounds like flavonoids, terpenoids, and phenolics unique to each plant species used in the synthesis process. *Escherichia coli* and *Staphylococcus aureus* were the most frequently tested bacterial strains, with main AuNPs effects comprising damage to their thiol and peptidoglycan cell walls. One hundred thirty three (Emmanuel et al. in Microb Pathog 113:295–302, 2017) articles expanded their evaluations to include fungi, protozoa and cytotoxic effects, with many reports encouraging results against antibiotic-resistant bacterial strains, reinforcing the potential of plant-based AuNPs as innovative antimicrobial therapy alternatives.

**Supplementary Information:**

The online version contains supplementary material available at 10.1186/s11671-026-04465-1.

## Background

Nanotechnology is an interdisciplinary field focused on developing materials with novel structures, properties, and morphologies at the nanoscale. Among these, nanoparticles (NPs), with dimensions below 100 nm, exhibit unique chemical and physical properties due to their high surface-to-volume ratios and zero-dimensional configuration. Metallic NPs, such as gold, silver, platinum, and titanium oxide, are of particular interest because of their applications across biomedical, industrial, and environmental areas, including drug delivery, cancer detection, catalysis, and antimicrobial treatments.

Traditional physical and chemical synthesis methods for metallic NPs—such as UV irradiation, laser ablation, and photochemical reduction—can be costly, energy-intensive, and environmentally hazardous. In response, green synthesis using plant extracts has emerged as a sustainable, low-cost, and non-toxic alternative. Plants act as reducing and stabilizing agents due to their bioactive compounds, including phenolics, terpenes, and alkaloids, allowing controlled nanoparticle formation while eliminating toxic reagents.

Plant-based metallic NPs have demonstrated promising antimicrobial effects. Nanoparticles interact with bacterial membranes, proteins, and genetic material, causing structural and functional damage. Their surface phytochemicals enhance these interactions, providing potential broad-spectrum activity and synergistic effects with antibiotics, particularly against multidrug-resistant bacteria. Given the growing threat of antimicrobial resistance to human, animal, plant, and environmental health, there is an urgent need for novel antibacterial strategies. In this context, green, plant-mediated gold nanoparticles (AuNPs) represent a promising alternative. This study aims to review the antimicrobial effects of plant-based AuNPs, highlighting the main plant compounds involved and their potential against antibiotic-resistant bacteria.

## Introduction

Nanotechnology comprises a major interdisciplinary area of ​​research aimed at developing new structures, properties, particle sizes and morphologies at the nanoscale [2]. Nanoparticles (NPs) are the most representative among nanomaterials, whose morphological properties are summarized in dimensions and diameters of less than 100 nm (10^− 9^ meters) [3]. The chemical and physical properties of NPs depend on their surface atoms [4] as increases surface-to-volume ratios decrease the grain size and melting point of the surface atom, directly affecting their chemical and physical properties [5]. However, to actually be characterized as NPs, they must have a zero-dimensional configuration, that is, all dimensions in nanometric scales [6].

Metallic NPs are of significant interest in this context, due to their potential applications in various areas [7] including biological, industrial and commercial fields, i.e., bioengineering, wastewater treatment, catalysis, electronics and agriculture [8]. Furthermore, some metal NPs, i.e., gold, silver, platinum, titanium oxide, exhibit unique and tunable surface plasmon resonance effects (SPR) [9] and can also be employed in the health and environmental areas, such as in cancer cell detection [10], drug administration [11] and encapsulation [12], as antimicrobial agents [13], nerve cell signaling stimulation [14], in sensor analysis [15], and as nano-barcodes and in membrane filtration [14].

Metallic NPs, like other NPs, can be obtained by various physical, chemical or biological processes [16], resulting in varied shapes such as spheres, cubes, tubes, prisms, octahedrons, and triangles [4]. Each shape exhibits different electrical, magnetic, catalytic and optical physical properties, which can be adjusted by modifying, for example, the length/diameter NP ratio [17]. These adjustments are made during synthesis by employing varying concentrations of reducing agents in the reaction medium, pH, temperature, reaction time and type of dispersant [18].

Numerous chemical and physical synthesis methods, such as UV irradiation [19], aerosol technologies [17], lithography, laser and ultrasonic fields [20] and photochemical reduction [21] are currently applied to obtain metallic NPs, although some disadvantages are noted. These include high costs [18] the use of toxic solvents [22] high energy consumption [23] and the generation of potentially hazardous products with environmentally harmful properties [3]. Thus, alternative methods for their synthesis are required [24]. In this sense, green NP syntheses using plants as raw material have emerged as a promising alternative [7].

Plant NPs are prepared by using plant-derived extracts employing different types of solvents, i.e., aqueous [25], methanolic [20], ethanolic [26] and hexanoic [27], under different concentrations [28], temperatures [29], pH and time [30] conditions. In this sense, plants can act as metal precursor reducing agents during NP synthesis [31], as they contain several antioxidant molecules [32], categorized according to their chemical structure, into three main groups, namely phenolic compounds, terpenes and alkaloids (nitrogen compounds) [16]. The functional groups of these plant molecules are part of the main reagents employed in NP syntheses [33], ensuring sustainable nanomaterials by eliminating the use of toxic solvents [6], and promoting metallic NP capping and stabilization [34]. This avoids additional reactions and NP aggregation, eliminating the need for the stabilizers used in conventional syntheses [35]. Low costs, safety and the ability to generate large production volumes also make plants an attractive option for the synthesis of metallic NPs [36].

Nanoparticles provide several opportunities for the development of innovative vehicles capable of delivering a wide range of drugs directly to their targets [17], as they may attach to drug molecules or trapped them in their central structures [37]. Concerning pathogenic organisms, such as bacteria, NPs behave as xenobiotics, causing damage to membranes, organelles and genetic material [38]. Plant molecules present on the surface of nanoparticles contain charged and reactive groups responsible for interacting with the proteins that make up the bacterial cell [39]. This may result in promising antimicrobial effects where the bacterial membrane and the charges contained in the surface molecules of the NPs are opposite, promoting molecular attraction and reactivity [35]. Antibacterial activities by weakening DNA replication and protein inactivation has also been noted [40]. However, the causes of the antimicrobial action of metallic plant NPs are still not fully understood [39] although it is known that their shape and surface reactivity can directly influence their antimicrobial properties [36].

The need for new antibacterial agents is partly due to the emergence of multidrug-resistant bacteria to known antibiotics [28], attributed to increasing misuse or exploitation of antibiotics [41] since the 1980s. This comprises a significant public health problem, increasingly limiting therapeutic options [42]. Because of this, the emergence and spread of antimicrobial resistance (AMR) has become a significant threat to human [35], animal [43], plants [44], and environmental health [37]. Thus, alternatives aimed at eliminating these pathogens become paramount [45].

Gold nanoparticles (AuNPs) exhibit exceptional biomedicinal properties. Gold belongs to the group of noble metals with inert properties, making it resistant to corrosion [24] and potentiating exhibit low toxicity to eukaryotic cells, depending on certain properties. In this sense one study reported that 5 nm AuNPs exhibited greater cytotoxicity comparedto 15 nm AuNPs in murine fibroblasts, demonstrating that the cytotoxicity of AuNPs is size-dependent [46]. Very high concentrations (> 600 µg mL^− 1^) can also easily penetrate cells and accumulate in organelles such as vacuoles [47], impairing cell viability. Regarding shape, rod-shaped and spherical AuNPs demonstrate less toxicity than other cellular forms [48]. Conversely, the same spherical AuNPs are more easily absorbed by cancer cells, resulting in damage, without altering the viability of normal cells [47]. Concerning charge, cationic AuNPs exhibit greater cytotoxicity than their anionic counterparts [46], as a anionic systems bind less efficiently to eukaryotic cell surfaces due to the electrostatic repulsion forces of negatively charged membranes [39].

With regard to bacteria, AuNPs exhibit a conduction band with six free electrons, making them ideal for interacting with thiol and amine molecules, which are very abundant in bacterial cell membranes [49]. Their strong binding to these functional groups allows them to adhere to the membrane and subsequently be ingested, interacting with intracellular structures such as proteins, lipids, and DNA [46], in addition to potentially damaging membrane integrity and inducing cytoplasmic leakage [50]. The presence of AuNPs in bacteria can also result in the production of cytotoxic reactive oxygen species [51], altered functioning of intracellular structures such as ribosomes, DNA, and enzymes [52], inactivation of transport proteins and the membrane electron transport chain [32], incorporation of metal ions [40] and inhibition of growth, communication and reproduction factors and biofilm formation [53].

Green plant-based metallic gold nanoparticles (AuNPs) display several antibacterial and antioxidant activities that can be harnessed to fight diseases [54]. They have been conjugated with known antibiotics to combat AMR [45], comprising AMR treatment candidates, and versatile platforms for therapeutic applications based on their properties [55] and size ranges, providing additional antibiotic interactions and enhancing AMR effects [32].

Regarding biosynthesized green AuNPs, their use would be advantageous as a novel antibacterial agents, as bacteria are unable to acquire resistance to NPs due to the high variety of plant molecules on their surfaces [56]. In this context, this study comprises a review on green plant-based metallic AuNPs and their antimicrobial effects. The main compounds present in plants and their possible effects against antibiotic-resistant bacteria are also discussed.

## **Methodology**

Articles published over the last ten years (2014–2025) were retrieved without language restrictions from the PubMed, Web of Science, Scopus, and Google Scholar databases using the following keywords: **“**gold nanoparticle,” “and” “plants,” “and” “antibacterial” in a bibliometric review. The choice of this specific time frame reflects the recent increase in studies emphasizing the sustainable synthesis of gold nanoparticles with antimicrobial applications. Data organization and a bibliometric analysis were performed using the R Studio software, and the selected articles were cataloged in Microsoft Excel. Each article was classified according to NP information (including synthesis and characterization), tested microbial strains, experimental results, and proposed antimicrobial action mechanisms.

The applied inclusion criteria comprised exclusively plant-based reducing agents and gold NPs; clearly described characterization techniques; direct antimicrobial activity assessments and identification of the main active plant compounds responsible for the observed antimicrobial effects. The exclusion criteria comprised studies in which gold was reduced using agents such as citrate and only later capped with plant extracts; articles employing other biological reducing agents such as fungi, viruses, or genetically modified bacteria, even when involving AuNPs and green synthesis approaches; studies focusing solely on the cytotoxic or antioxidant properties of plant-based AuNPs; and articles with incomplete or redundant data regarding nanoparticle concentrations or those with superficial discussion of the obtained results. These excluded sources were, however, used to complement the discussion of the reported findings.

## Results and discussion

**Fig. 1:**
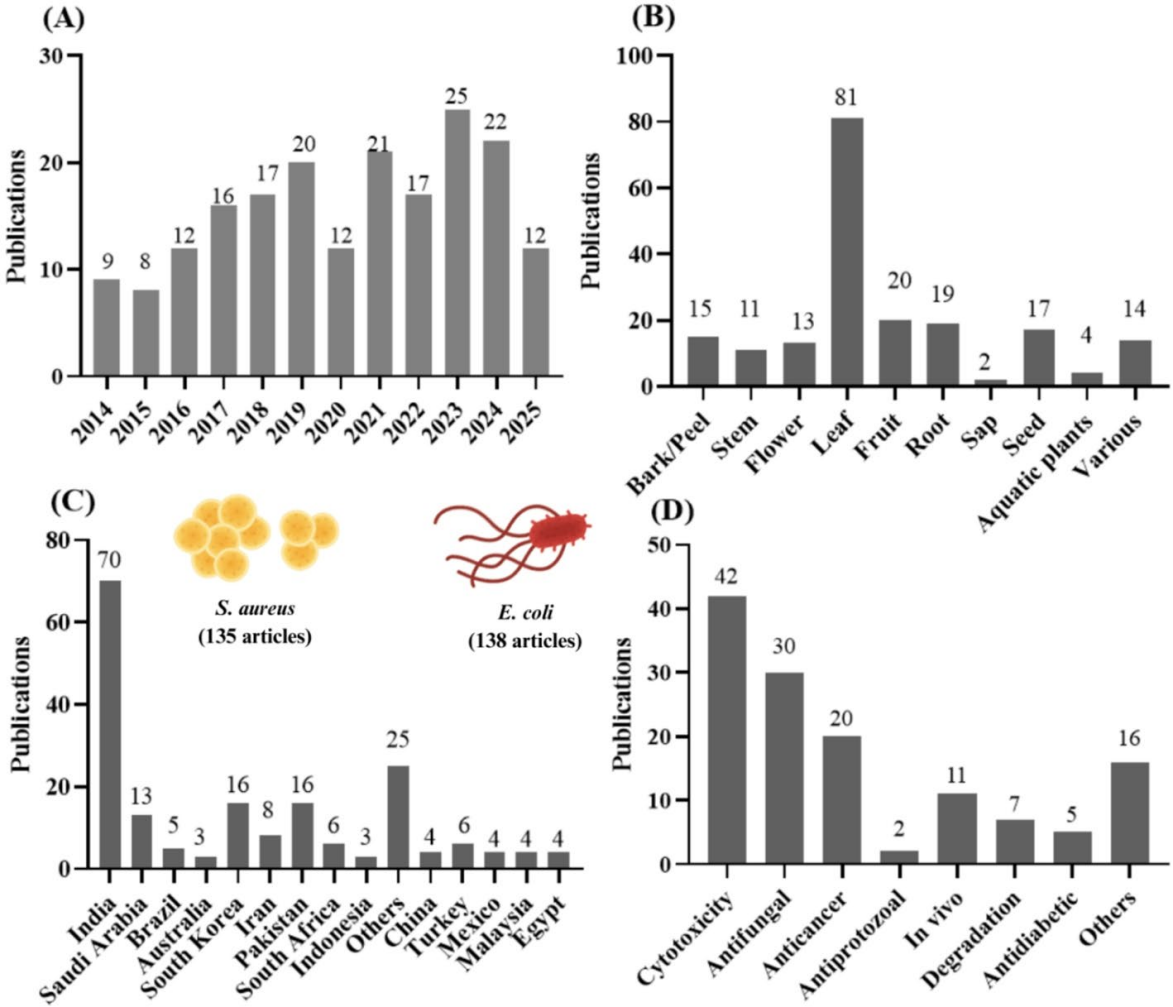
Published research on plant AuNPs displaying antimicrobial applications organized by year (**A**), plant part (**B**), country (**C**) and other additional applications (**D**), in addition to the main bacteria that were most tested

A total of 190 articles from 42 countries were retrieved (Fig. 1C), with India leading the number of publications on this topic (70 articles). Regarding the plant parts used for AuNP synthesis (Fig. 1B), leaves were predominant, appearing in 42.6% of the studies (81 articles), followed by fruits and roots, with 20 and 19 articles respectively.

The antimicrobial potential of the synthesized AuNPs was tested against 55 different bacterial strains, and the most applied methodology was agar isolation at a 0,5 McFarland scale (~ 1.5 × 10^8^ UFC mL). *Escherichia coli* was the most frequently studied, appearing in 130 articles (72.2%), closely followed by *Staphylococcus aureus* in 128 studies (71.1%). Other commonly tested bacteria included *Pseudomonas aeruginosa* (74 studies), *Bacillus subtilis* (50 studies), and *Klebsiella pneumoniae* (41 studies).

*Escherichia coli*, an indicator of poor sanitation, is of particular concern according to the World Health Organization (WHO) [42], as it is a leading cause of nosocomial infections such as peritonitis, catheter-associated urinary tract infections, ventilator-associated pneumonia [57], and fatal prolonged diarrhea [43]. This bacterium is facultatively anaerobic and commonly found in soil, vegetables, water, and undercooked meats, exhibiting high adaptability [58]. Additionally, it can develop resistance to antibiotics, with beta-lactam resistance being the most common [59].

*Staphylococcus aureus* causes a wide range of infections from minor skin conditions to severe illnesses such as meningitis, pericarditis, bacteremia, and toxic shock syndrome [43, 57]. It is particularly dangerous for immunocompromised patients with prolonged hospital stays [60]. Its high susceptibility to acquiring antibiotic resistance is mainly due to plasmid-encoded penicillinase enzymes, which hydrolyze and inactivate beta-lactam antibiotics [44]. Methicillin-resistant *S. aureus* (MRSA) is recognized globally as a serious public health issue [42] with seven studies specifically testing AuNPs against this resistant strain. In regions such as Northeastern Brazil, up to 90% of MRSA infections are untreatable with standard antibiotics, contributing to its rising prevalence in healthcare and community settings [37, 60].

Cytotoxicity assays to evaluate the safety of these nanoparticles are still limited, with only 42 out of the 190 studies (< 25%) included such tests. Nevertheless, this is an important area to encourage, as AuNPs can exhibit diverse bioactivities beyond antimicrobial effects, including antifungal (30 studies), anticancer (20 studies), larvicidal and antiprotozoal activities (2 studies) (Fig. 1D).

### Antimicrobial effects mediated by plant-derived AuNPs

Plants exhibit remarkable versatility as sources of reducing agents for nanoparticle synthesis, with extracts obtained from a wide range of plant parts, including roots [49], seeds [8, 61, 62], leaves [54, 56], flowers [63, 64] fruits [65, 66] bark [28, 67], stems [20], sap or resin [68] aquatic plants [69], and combinations of different parts such as leaves and flowers or bark and roots [70, 71].

**Fig. 2:**
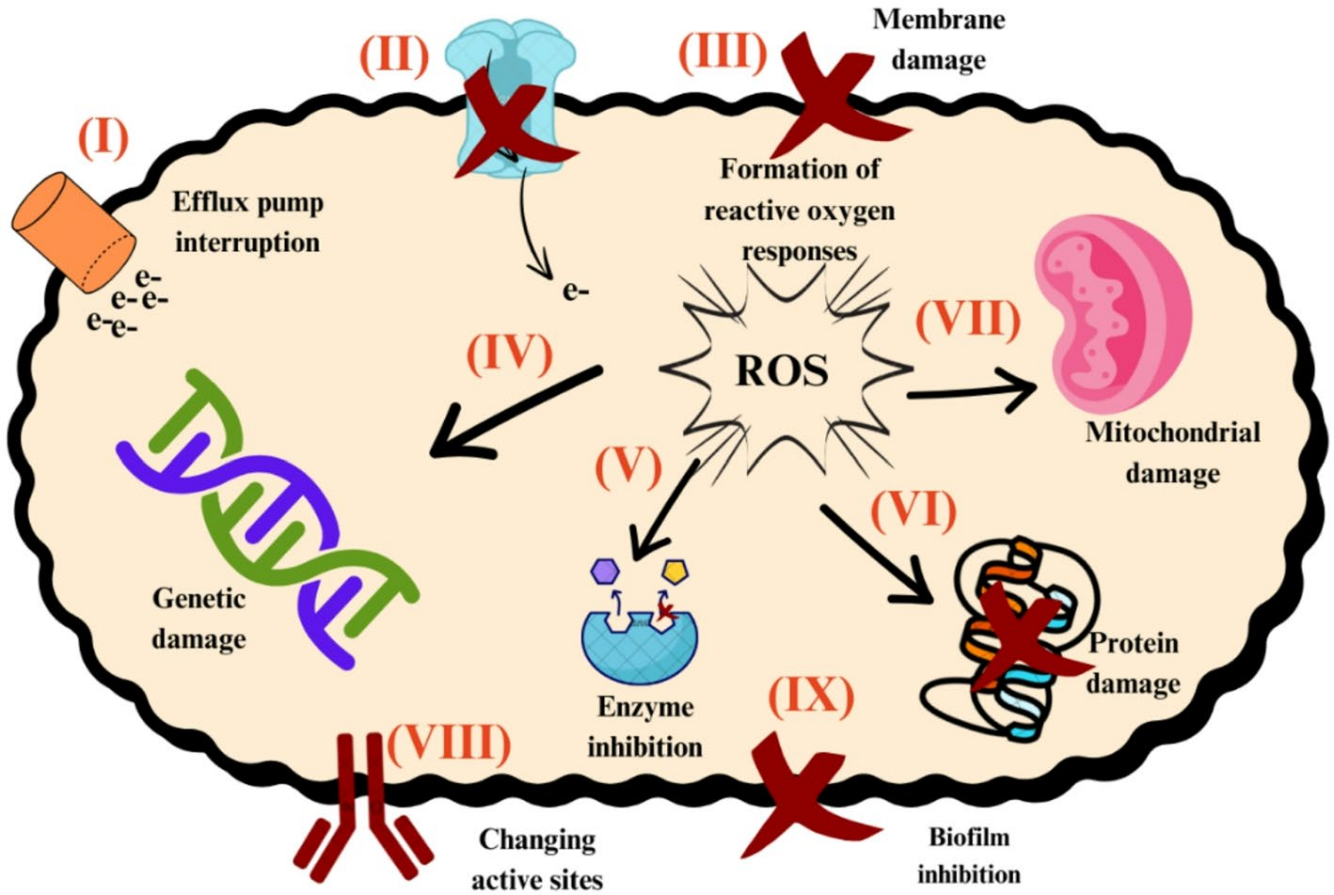
Potential antibacterial AuNP mechanisms of action according to the retrieved studies on green plant-based metallic AuNPs and their antimicrobial effects

The antimicrobial activity of AuNPs synthesized from these diverse plant sources has been heterogeneous. Some studies reported predominant efficacy against Gram-positive bacteria [62], while others observed stronger effects against Gram-negative strains [61], likely due to differing mechanisms of action. A simplified scheme illustrating the potential antibacterial mechanisms of AuNPs is depicted in Fig. 2. These mechanisms include interference with bacterial efflux pumps (reaction I), disruption of electron transport chain (reaction II), membrane damage (reaction III), genetic damage (reaction IV), enzyme inhibition (reaction V), disruption of protein (reaction VI), as well as mitochondrial (reaction VII), active site alterations (reaction VIII) and biofilm formation (reaction IX).

### Gold nanoparticles derived from seed extracts

Seventeen studies employed seeds in the synthesis of AuNPs (Table 1). Seeds are known to possess antinociceptive and anti-inflammatory [62], antimicrobial [8], antiviral, antifungal [28], anticancer [47] and antihyperglycemic properties [61]. They have also been widely used in the treatment of various diseases due to their high antioxidant potential [72]. In the context of AuNP synthesis, seed-derived compounds facilitate both the reduction of gold ions and the stabilization of the resulting nanomaterials, leading to the sustainable production of NPs [73]. Regarding antimicrobial potential, most synthesized seed-derived AuNPs were effective against Gram-positive strains. For example, AuNPs synthesized using aqueous seed extract from bottlebrush (*Callistemon citrinus*) exhibited dose-dependent inhibitory activity against *Staphylococcus enteritidis* and *S. aureus* at 15.6 mg mL⁻¹ [62]. *S. aureus* also showed sensitivity to AuNPs synthesized from white acacia (*Moringa oleifera*) [74], black cumin (*Nigella sativa*) [75], caraway (*Trachyspermum ammi*) [72], and quinoa (*Chenopodium quinoa*) [76] at 200, 10, 10 and 0.2 mg mL⁻¹, respectively, all resulting in over 50% bacterial growth inhibition.

**Table 1:** Studies on the synthesis of green gold nanoparticles from seed extract, including plant species, synthesis methods, characterization techniques, applied concentrations, bacterial strains tested, exposure time, and authors

Species	Synthesis Method	NP Characterization	NP concentrations	Description of Bacteria Used	Experimental Design and Culture Medium	Exposure Time	References
Bottlebrush *(Callistemon citrinus)*	30 g of crushed sample in 250 mL distilled water, stirred at 200 rpm for 24 h and filtered; 12.5 mL of plant extract added to 90 mL HAuCl₄ (1 mmol L⁻¹) solution, incubated for 6 h with continuous stirring	UV-Vis, TEM, XRD, SEM, FT-IR, and DLS	20 mg mL⁻¹, 50 µg mL⁻¹ (antimalarial and antiprotozoal), 15.625–62.5 mg mL⁻¹ (antimicrobial)	*Strains: Escherichia coli*,* Vibrio alginolyticus*,* Salmonella typhi*,* Staphylococcal enteritis*,* Staphylococcus aureus*,* Listeria ivanovii*,* Mycobacterium smegmatis; Protozoa: Plasmodium falciparum*,* Trypanosoma brucei brucei*	Minimum Inhibitory Concentration (MIC); Mueller-Hinton broth	24 h	Rotimi et al., [62]
Mango *(Mangifera indica)*	10 g seed powder in 100 mL distilled water for 5 h under constant stirring at room temperature, filtered; 60 mL extract added to 40 mL HAuCl₄ (1 mmol L⁻¹) at room temperature until color change	UV-Vis, TEM, XRD, Zeta Potential, FT-IR, Raman	10–100 µg mL⁻¹	*Staphylococcus aureus and Escherichia coli*	Minimum Inhibitory Concentration (MIC); Luria-Bertani (LB) broth	4 h	Vimalraj et al., [8]
Durian *(Durio zibethinus)*	60 mg seeds in 90 mL HAuCl₄ (5 mmol L⁻¹) at room temperature for 1 h, refluxed with vigorous stirring at 97 °C for 5–6 h	UV-Vis, TEM, SEM, XRD, Zeta Potential, FTIR, EDAX	1000 and 1500 mg mL⁻¹	*Pseudomonas desmolyticum and Staphylococcus aureus*	Disc diffusion; Mueller-Hinton agar	24 h	Vinay et al., [73]
True cardamom *(Elettaria cardamomum)*	2 g seeds boiled in 100 mL deionized water for 5 min and filtered; 1 mL extract added to 30 mL HAuCl₄ (2.5 × 10⁻⁴ mol L⁻¹) at boiling (373 K) for 2 min; repeated with 5, 10, 15, 20 mL extract to obtain colloids B₂–B₅	UV-Vis, TEM, SEM, XRD, FTIR	165, 330, 495, 660 µg mL⁻¹; antibiotic gentamicina	*Escherichia coli*,* Staphylococcus aureus*,* Pseudomonas aeruginosa*	Disc diffusion; Mueller-Hinton agar	24 h	Rajan et al., [61]
Wild rue *(Peganum harmala L)*	5 g dried seeds in 250 mL distilled water at 80 °C for 90 min, filtered; 4 mL HAuCl₄ added to 100 mL extract (final 0.558 mmol L⁻¹ saline solution) at room temperature	UV-Vis, TEM, SEM, XRD, FTIR, EDX, FESEM	100, 150, 200 µg mL⁻¹	*Escherichia coli and Staphylococcus aureus*	Minimum inhibitory concentration; Luria-Bertani (LB) broth	24 h	Moustafa et al., [77]
Barley *(Hordeum vulgare)*	10 g grains with 90 mL distilled water at 100 °C for 20 min; HAuCl₄ 0.5–2.5 mmol L⁻¹; 60–90 °C, 1–30 min	TEM, SEM, EDS, AFM, DLS, FT-IR, MALDI-TOF, ICP-MS	1–8 µg mL⁻¹	*Escherichia coli and Pseudomonas aeruginosa*	Minimum Inhibitory Concentration (MIC); Mueller-Hinton broth	24 h	Singh et al., 2024
White acacia *(Moringa oleifera)*	Powder mixed with methanol 1:10 (m/v); aqueous solutions with 2.5, 5, 10 mg mL⁻¹ extract, 1:1 (v/v) with Au (III) chloride, stirred 24 h at room temperature, centrifuged at 2500 rpm for 20 min	UV-Vis, TEM, FTIR	200, 400, 800, 1600 mg mL⁻¹	*Escherichia coli and Staphylococcus aureus*	Minimum Inhibitory Concentration (MIC); Mueller-Hinton broth	24 h	Figueroa et al., [73]
Mahua *(Madhuca longifolia)*	5 g dried seeds powdered, boiled under reflux for 60 min, filtered; 0.4 mL extract per 1 mL HAuCl₄, pH 7.0	UV-Vis, TEM, FTIR	25, 50, 75, 100 µg mL⁻¹	*Micrococcus luteus and Proteus vulgaris*	Minimum Inhibitory Concentration (MIC); Lysogeny broth (LB)	–	Dhayalan et al., [55]
Black ebony *(Diospyros celebica)*	Extract: salt (2:1) at 60 °C for 2 h	UV-Vis, Zeta potential, TEM	2.3 µg mL⁻¹ to 0.575 µg mL⁻¹	*Bacillus subtilis*,* Staphylococcus aureus*,* Escherichia coli*,* Pseudomonas aeruginosa*	Minimum Inhibitory Concentration (MIC); Mueller-Hinton broth	24 h	Ariani et al., [78]
Quinoa *(Chenopodium quinoa W.)*	4 g starch in 100 g total solution gelatinized at 82 °C for 30 min; five types of biofilms: control film, biofilms with 5% and 2.5% AuNPs, and two control biofilms (5% and 2.5% blank solution), incubated 35 °C for 16 h	TEM, SEM, TGA	0.1 and 0.2 mg mL⁻¹	*Escherichia coli and Staphylococcus aureus*	500 µL on 20 mm² biofilms; 100 µL aliquots transferred to saline, then 10 µL plated on TSA agar	10 h	Pagno et al., [76]
Caraway *(Trachyspermum ammi)*	1 g extract in 100 mL methanol and/or water; 5 mL aliquots added to 5, 10, 15, 20 mL HAuCl₄, stirred on heated shaker at 40–60 °C for 60 min	UV-Vis, FT-IR	2.5, 3.33, 5, 10 µg mL⁻¹	*Staphylococcus aureus*,* Klebsiella pneumoniae*,* Bacillus subtilis*	Disc diffusion; Mueller-Hinton agar	24 h	Bawazeer et al., [72]
Mango *(Mangifera indica)*	1 g dried powder extracted with 100 mL bidistilled water, boiled 5 min; 6 mL extract added to 40 mL HAuCl₄ 1 mmol L⁻¹ at 25 °C in dark for 24 h	UV-Vis, XRD, TEM, FT-IR, SAED, Zeta Potential	10, 20, 30, 40, 50 mg mL⁻¹; DMSO	*Bacteria: Bacillus cereus*,* Escherichia coli*,* Staphylococcus aureus*,* Klebsiella pneumoniae*,* Salmonella typhimurium; Fungi: Cryptococcus neoformans*,* Candida albicans*,* Candida glabrata*	Agar well diffusion; Mueller-Hinton agar	24 h	Donga et al., [79]
Vidanga *(Embelia ribes)*	1.25 g dried powder in 50 mL deionized water at 60 °C for 1 h; 1 mL extract added to HAuCl₄ (0.1 mmol L⁻¹) in 0.1–1 mL, final volume adjusted to 5 mL, reaction at room temperature until color change	UV-Vis, DLS, HR-TEM, FT-IR, XRD	250, 500, 750, 1000 µg mL⁻¹	*Escherichia coli and Staphylococcus aureus*	Agar well diffusion; Mueller-Hinton agar	24–48 h	Dhayalan et al., [55]
Mint flower *(Lallemantia royleana)*	HAuCl₄·3 H₂O (1 mmol L⁻¹) prepared as solution A; 0.2% (m/v) extract solution as B; 2–20 mL B added to 20 mL A under vigorous stirring at 25–80 °C, pH 3.5–12 for 24 h	UV-Vis, EDX, TEM, XRD, AFM, DLS	180 µg mL⁻¹ (disc); roxithromycin 100 ppm, 10 µL; 180, 90, 45, 22.5, 11.25 µg mL⁻¹ MIC	*Agrobacterium tumefaciens*,* Bacillus subtilis*,* Escherichia coli*,* Staphylococcus aureus*,* Pseudomonas aeruginosa*	Agar well diffusion; Mueller-Hinton agar and MIC; Mueller-Hinton broth	24 h	Iram et al., [232]
Castor *(Ricinus communis)*	100 mg seeds dissolved in 100 mL methanol; 100 mL extract reacted with 1 mmol L⁻¹ HAuCl₄ in 1:1–1:5 (extract: salt), 30–80 °C, 1–24 h	UV-Vis, FT-IR, DLS, TEM	1 mg mL⁻¹; ampicillin	*Bacillus cereus*,* Salmonella typhi*,* MRSA*,* Escherichia coli*,* Klebsiella pneumoniae*	Agar well diffusion; Mueller-Hinton agar	24 h	Rahman et al., [80]
Caraway *(Trachyspermum ammi)*	20 g seed powder mixed in 500 mL bidistilled water for 24 h; 6 mL extract added to 2 mL HAuCl₄ (10 mmol L⁻¹) on magnetic stirrer 30 min, then microwave 2 min (2.45 GHz, 300 W)	UV-Vis, XRD, TEM, DLS	30 mg mL⁻¹	*Listeria monocytogenes*,* Serratia marcescens*	Minimum Inhibitory Concentration (MIC); Lysogeny broth (LB)	24 h	Perveen et al., [47]
Black cumin *(Nigella sativa)*	200 g air⁻dried seeds in 200 mL water for 12 h; 2 mL extract added to 30 mL HAuCl₄ (1 mmol L⁻¹) at 100 °C, stirred 1 min, repeated with 5–8 mL	UV-Vis, XRD, TEM	3, 5, 10 µg mL⁻¹ (disc); 2–10 µg mL⁻¹ (MIC); 20–80 µg mL⁻¹ (anti-biofilm)	*Staphylococcus aureus*,* Vibrio harveyi*	Disc diffusion; Mueller-Hinton agar	24 h	Manju et al., [75]

*Thermogravimetric analysis (TGA), Gas chromatography-mass spectrometry (GC-MS/MS) and High performance liquid chromatography/ultraviolet-visible (HPLC/UV-VIS), Electron diffraction (SAED), X-ray diffraction (XRD), Dynamic light scattering (DLS), Energy dispersive spectroscopy (EDX), Energy dispersive spectroscopy (EDS), Fourier transform infrared spectroscopy (FTIR), Raman spectroscopy (Raman), Ultraviolet-visible spectroscopy (UV-Vis), Field emission splitting electron microscopy (FE-SEM), Transmission electron microscopy (TEM), High resolution transmission electron microscopy (HRTEM), Scanning electron microscopy (SEM), Zeta Potential (Zeta)

Additionally, AuNPs synthesized from black cumin and quinoa inhibited 98% and 78%, respectively, of *S. aureus* biofilm formation, as well as 99% of *E. coli* biofilm formation in anti-biofilm assays [76]. These AuNPs also sensitized the Gram-positive strain *Serratia marcescens*, with 81% inhibition observed [75]. Other seed-derived AuNPs active against Gram-positive bacteria included those synthesized from Mahua (*Madhuca longifolia*), which inhibited *Micrococcus luteus* growth by 50% at 100 µg mL⁻¹ [55]. The same author also tested AuNPs synthesized from Vidanga (*Embelia ribes*) at 1000 µg mL⁻¹, which exhibited greater *E. coli* and *S. aureus* inhibition compared to the standard antibiotic tetracycline.

Two studies evaluated AuNPs synthesized from mango (*Mangifera indica*) seeds. In the experiments conducted by Donga; Bhadu; Chanda [79], these NPs inhibited the growth of *Bacillus cereus* and *Bacillus subtilis* by over 70% at the lowest concentrations tested (20 and 30 mg mL⁻¹, respectively). Vimalraj et al. [8] reported a dose-dependent reduction in *E. coli* and *S. aureus* was observed with increasing AuNP concentrations, with an LC₅₀ of 25 µg mL⁻¹ for both species. These results demonstrate that mango seed-derived AuNPs exhibit high antimicrobial efficacy against a broad range of both Gram-positive and Gram-negative bacteria. Similarly, AuNPs synthesized from caraway seeds also showed antimicrobial activity in two studies, inhibiting *Listeria monocytogenes* [47] and *B. subtilis* [72] by over 70%, in addition to reducing biofilm formation by 58%.

Seeds contain numerous terpene compounds, such as caryophyllene, limonene, and pinene [62]. AuNPs reduced by terpenes often acquire a negative surface charge, which can strongly interact with the positively charged membranes of Gram-positive bacteria. This positive charge is due to the presence of Intercellular Adhesion Polysaccharide (PIA), a key component of their biofilm and membrane structures [79]. Other bioactive groups of interest include polyphenols such as gallic acid, gallotannins, ellagic acid, xanthones (e.g.., mangiferin), methyl gallate, digallic acid, α-gallotannin, and β-glucogallin, found in mango [81] and Macadamia (*Macadamia integrifolia*) seed extracts [28]. These molecules contain hydroxyl and carbonylamide groups that, at high concentrations, can disrupt microbial cell integrity by binding to carbohydrates, lipids, and proteins in the thick peptidoglycan layer of Gram-positive strains [78]. This interaction alters membrane permeability [82], leading to the formation of cavities, cracks, and pores, ultimately causing cell death via cytoplasmic leakage [16]. These findings support the strong antibacterial potential of seed-derived AuNPs [8, 62].

Rajan et al. [61] synthesized AuNPs using seed extract from fresh fruits of true cardamom (*Elettaria cardamomum*). Three bacterial species (*S. aureus*, *Pseudomonas aeruginosa*, and *E. coli*) were sensitive to the synthesized nanoparticles at a concentration of 0.5 mg mL⁻¹. In another study, fresh durian (*Durio zibethinus*) seeds were used against *Pseudomonas desmolyticum*, a Gram-negative bacterium, showing high dose-dependent antimicrobial efficiency [73] ., a finding also reported by Rotimi et al. [62] and Vimalraj et al. [8].

**Fig. 3:**
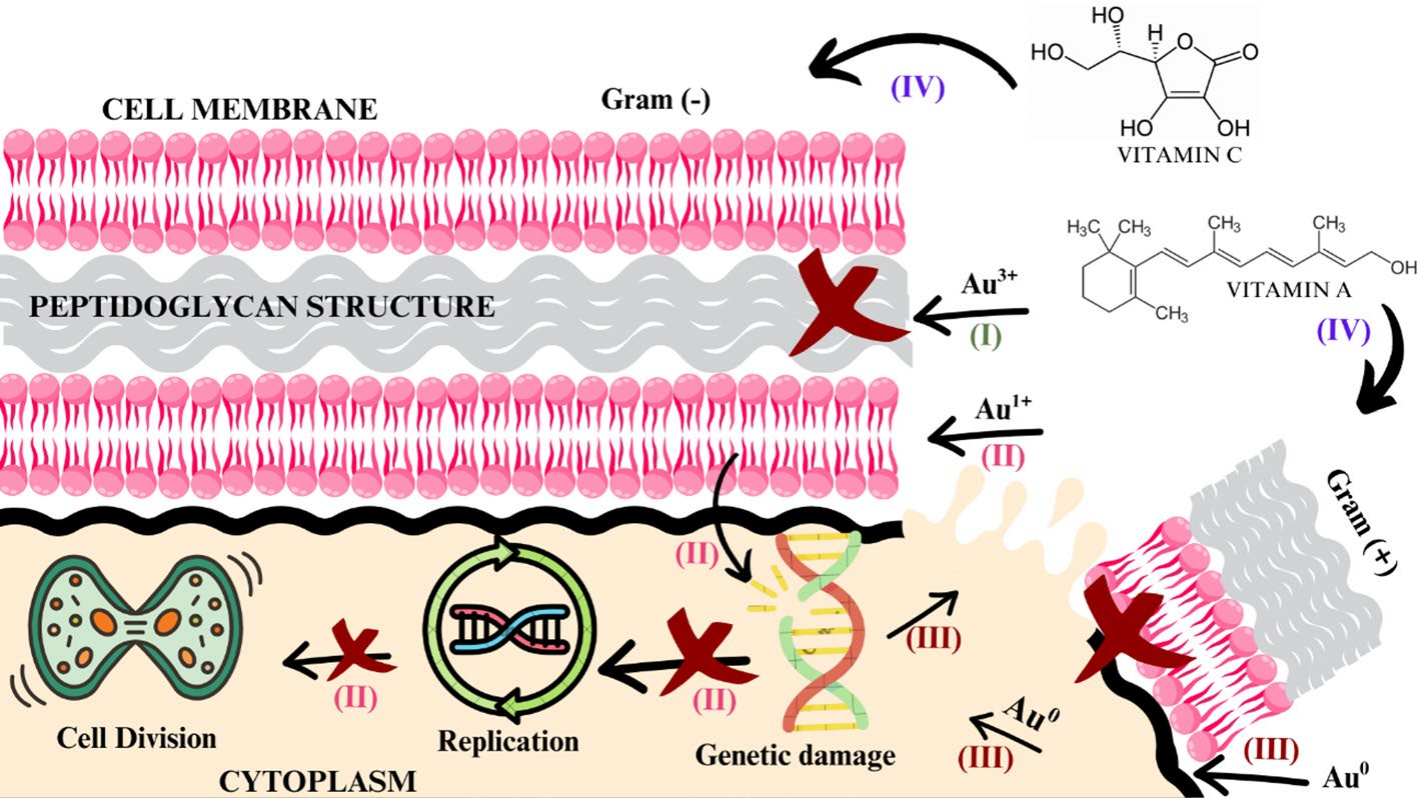
Scheme representing (I) the reaction of Au^3+^ with the peptidoglycan wall of a gram-negative bacterium, (II) Au^1+^ reaction, causing genetic damage and preventing cell replication and division, (III) Au^0^ causing cell leakage and (IV) Au^0^ reaction between vitamin A in bacterial gram-positives membranes, according to the retrieved studies on green plant-based metallic AuNPs and their antimicrobial effects

Seeds obtained from fresh fruits retain various bioactive molecules that are often degraded over time during storage or by lyophilization processes. These include vitamins A, C, and E, which may exert harmful effects on Gram-negative [73].This antimicrobial activity may also be influenced by the presence of residual Au¹⁺ and Au³⁺ ions, as well as by the composition of the nanoparticle surface coating formed during the bioreduction process [2]. These ions, although considered products of an incomplete synthesis reaction—since gold is expected to be fully reduced from Au³⁺ to its elemental form (Au⁰)—may still contribute to antibacterial activity [4] (Fig. 3, reaction IV).

Seed extracts can also be combined with extracts from other parts of the same plant. In one study, the combination of aqueous extracts from seeds and leaves of Syrian rue (*Peganum harmala* L.) showed promising antimicrobial activity against *E. coli* and *S. aureus*, with inhibition observed at the highest concentration tested (200 mg mL⁻¹) [83]. These results outperformed those reported by Ullah et al. [84], who used AuNPs synthesized from leaf extracts of the same species and observed antimicrobial activity only against the Gram-positive bacteria *B. subtilis* and *S. aureus* (72% and 76%, respectively), compared to standard antibiotic. No significant inhibition was reported for *E. coli*,* P. aeruginosa*, or *Salmonella typhi*. The enhanced effect observed against *E. coli* in the seed–leaf extract combination suggests a synergistic interaction between the plant components.

The results reported by Moustafa and Alomari [77] are consistent with those Rajan et al. [61], who also observed inhibition of both Gram-positive and Gram-negative bacteria. This broader activity may be attributed to the greater molecular diversity in combined extracts, for example, catechins and quercetins present in seeds [11], along with alkaloid compounds found in leaves [66]. These compounds are charged and contain reactive hydroxyl (–OH) groups, which, when incorporated onto the surface of AuNPs, enhance their antimicrobial properties [80].

**Fig. 4:**
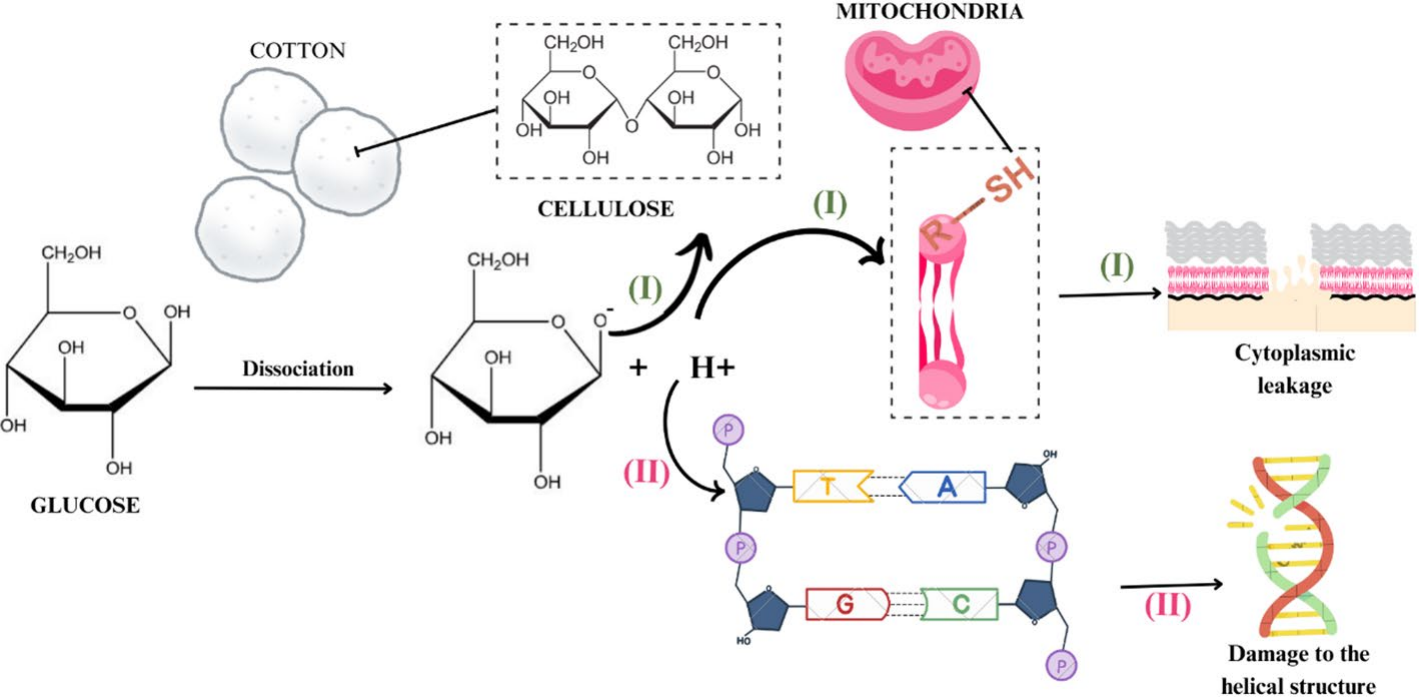
Reaction mechanisms between (I) glycosate and cotton cellulose and the H^+^ ion with the thiol group in promoting cytoplasmic leakage; (II) reaction mechanism between H + and the DNA phosphate group, according to the retrieved studies on green plant-based metallic AuNPs and their antimicrobial effects

In this context, the surface charge of AuNPs facilitates interactions with sulfur-containing membrane proteins and the phosphorus groups present in bacterial DNA, leading to cross-linking [85, 86]. These interactions can cause membrane rupture, disruption of the helical structure of nucleic acid chains [87], or primarily interfere with bacterial cell division, ultimately resulting in cell death [88] (Fig. 4, reaction I).

### Gold nanoparticles derived from root extracts

Roots were used in the synthesis of AuNPs in 19 of the retrieved studies (Table 2). In traditional Arab medicine, rhizomes have been employed to treat various conditions such as stomach pain, dizziness, headaches, and as diaphoretic and diuretic agents [49]. More recent research has demonstrated neuroprotective effects of rhizomes against stroke and chemically induced neurodegeneration in rats [89].

**Table 2:** Studies on the synthesis of green gold nanoparticles from root extract, including plant species, synthesis methods, characterization techniques, applied concentrations, tested bacterial strains, exposure time, and authors

Species	Synthesis Method	NP Characterization	NP concentrations	Bacteria Described	Experimental Design and Culture Medium Used	Exposure Time	References
Sweet Flag *(Acorus calamus)*	For extraction at 60 °C and 100 °C, 30 g of washed rhizome was ground with 90 mL of water and heated for 15 min using a Soxhlet apparatus at 60 °C and 100 °C separately and filtered; 2.5 mL of appropriate extract, 2.5 mL of chloroauric acid 0.001 mmol L⁻¹ and 1 mL of buffer solution at appropriate pH (4, 7, and 9.2) were added. The mixture was stirred at 240 rpm using a magnetic stirrer until color change	UV-Vis, TEM, XRD, FT-IR	1 g cotton with 750 mg mL⁻¹ AuNPs	*Staphylococcus aureus and Escherichia coli*	Cotton coating using pad-dry-cure method	24 h and 48 h	Ganesan et al., [49]
Apricot *(Mammea suriga)*	50 g of root bark powder in 200 mL of water with continuous stirring for 5 h and filtered; 5 and 15 mL of aqueous root bark extract with 10 mL of HAuCl₄ for 24 h at 80 °C; centrifuged at 10,000 rpm for 20 min and dried at 80 °C in an oven	UV-Vis, SEM, EDX	1 and 3 mmol L⁻¹	*Bacillus subtilis*,* Staphylococcus aureus*,* Pseudomonas aeruginosa*	Disk diffusion; Mueller-Hinton Agar	18 h	Poojary, [90]
Ginger *(Zingiber officinale*) and Curcumin *(Turmeric rhizome)*	20 g of ground ginger in 250 mL deionized water boiled for 20 min; 1 mL of 1 mmol HAuCl₄ diluted with deionized water to a final volume of 10 mL and boiled. Then, 1 mL of ginger extract was added to the boiling solution and stirred at 600 rpm until solution turned purple. 3.68 mg of curcumin rhizome (95% purity) dissolved in 2 mL of 10 mmol L⁻¹ NaOH solution, volume adjusted to 10 mL with deionized water. 1 mL of 1 mmol L⁻¹ HAuCl₄ added to 8 mL of water. Then, 1 mL of freshly prepared curcumin solution added dropwise under stirring at 600 rpm for 20 min	UV-Vis, TEM, Zeta Potential, DLS, XRD, FT-IR	80 mg mL⁻¹ (ginger); 1.84 mg mL⁻¹ (curcumin)	*Pseudomonas aeruginosa*,* Staphylococcus aureus*,* Escherichia coli*	Minimum Inhibitory Concentration (MIC); Mueller-Hinton Broth	24 h	Kalantari et al., [91]
Curcumin *(Turmeric rhizome)*	10 g of turmeric immersed in 150 mL deionized water; 8 mL of 1 mmol L⁻¹ gold salt solution combined with 2 mL of turmeric extract for 5 min	FT-IR, Zeta Potential, DLS, XRD, TEM	14 mg mL⁻¹	*Staphylococcus aureus*,* Escherichia coli*	Nutrient agar	18 h	Mohammad et al., [92]
Neem *(Azadirachta indica)* and Ginger *(Zingiber officinale)*	5 g of each compound in 100 mL water separately. 10 mL of neem and ginger extracts added to 100 mL of aqueous gold chloride 1 mmol L⁻¹ at room temperature until color change	EDS, SEM	5 mg mL⁻¹	*Bacteria: Streptococcus mutans*,* Staphylococcus aureus*,* E. faecalis; Fungus: Candida albicans*	Well diffusion; Mueller-Hinton Agar	24 h	Kasabwala et al., [233]
Stinking Benjamin *(Trillium govanianum)*	20 g of dried and powdered rhizome in 50 mL distilled water; addition of 10 mg mL⁻¹ to 1 mmol L HAuCl₄ solution in three ratios: 1:1, 1:5, 1:10 (salt: extract) at 25 °C with constant stirring for 24 h	UV-Vis, FE-SEM, SEM, FT-IR, XRD	40 mg mL⁻¹ (1:10) Azithromycin (50 µg, 6 µL–1) Gram-positive, Ciprofloxacin (30 µg, 6 µL–1) Gram-negative, Clotrimazole (50 µg, 6 µL–1) fungi. AuNPs 6–18 µL–1	*Bacteria: Staphylococcus aureus*,* Pseudomonas aeruginosa*,* Escherichia coli*,* Bacillus subtilis*,* Klebsiella pneumoniae*,* Xanthomonas campestris; Fungi: C. albicans*,* Curvularia*,* R. oryzae*,* A. niger*,* A. alternaria*,* Paecilomyces*	Disk diffusion; Mueller-Hinton Agar	nd	Zaman et al., [93]
Licorice *(Glycyrrhiza uralensis)*	10 g of root powder extracted for 1 h in 100 mL distilled water at 100 °C; HAuCl₄ (1 mmol L⁻¹) at 80 °C until color change	FE-TEM, XRD	500, 1000, 1500 µg mL⁻¹	*Escherichia coli*,* Staphylococcus aureus*,* Pseudomonas aeruginosa*,* Salmonella enterica*	Disk diffusion; Mueller-Hinton Agar	24 h	Nguyen et al., [89]
Ginger *(Zingiber officinale)*	45 mL Milli⁻Q water and 5 mL root extract; reaction parameters optimized for pH 4–10, reaction temperature 20–70 °C, time 0–210 min, metal ion concentration 0.25–4 mmol L⁻¹	UV-Vis, FE-SEM, FT-IR, XRD	0.1 mg mL⁻¹ each	*Staphylococcus spp.*,* Listeria spp.*,* Bacillus spp.*	Disk diffusion; Mueller-Hinton Agar	24 h	Velmurugan et al., [94]
Leadwort *(Plumbago zeylanica roots)*	1:1 ratio, 24 h at 70 °C	UV-Vis, FTIR, XRD, TEM, DLS	25 µg mL⁻¹	*Escherichia coli*,* Staphylococcus aureus*,* Acinetobacter baumannii*	Disk diffusion; Mueller-Hinton Agar	18 h	Chopade et al., [100]
Black Gold *(Curculigo orchioides)*	25 g of root boiled for 30 min in 100 mL sterile water. 1 mL of extract added to 5 mL HAuCl₄·3 H₂O (1 mmol L⁻¹) at 80 °C for 2 h	UV–Vis, HR-TEM, XRD, EDX, FT-IR	50 and 100 µg mL⁻¹	*Escherichia coli*,* Klebsiella pneumoniae*,* Proteus vulgaris*,* Staphylococcus aureus*,* Serratia marcescens*	MIC; Mueller-Hinton Broth	24 h	Thamilchelvan et al., [95]
African Asparagus *(Asparagus racemosus)*	5 g root powder in 50 mL distilled water; 1:1 (v/v) extract and HAuCl₄ (1 mmol L⁻¹) at 28 °C until deep wine red color, ensuring AuNP formation	FT-IR, SAED, SEM, TEM, FE-SEM, EDX, Zeta Potential, DLS	20, 40, 80 µg mL⁻¹ (Streptomycin 25 µg and Gentamicin 25 µg)	*Escherichia coli*,* Bacillus subtilis*,* Klebsiella pneumoniae (urine)*,* Pseudomonas aeruginosa*,* Staphylococcus aureus*	Disk diffusion; Luria-Bertani (LB) Agar	24 h	Amina et al., [96]
Ginger *(Zingiber officinale)*	50 g extracted with ethyl acetate in microwave at 630 W for 15 min obtaining 8.7 g extract; 5 mL extract added individually to reaction vessels containing 50 mL HAuCl₄ (1 mmol L⁻¹) at room temperature until color change	Extract: GC–MS; NPs: UV-Vis, TEM, FT-IR, Zeta Potential, AAS, DLS	17.95, 8.97, 4.48, 2.24, 1.12, 0.56, 0.28, 0.14 µg mL⁻¹ (Streptomycin 30 µg mL⁻¹)	*Staphylococcus aureus*,* Escherichia coli*	Disk diffusion; Mueller-Hinton Agar	24 h	Yadi et al., [71]
Burdock *(Arctium lappa)*	2 g powder with 80 mL ethanol at 40 °C for 24 h; 1 mL extract added to boiling 50 mL HAuCl₄·3 H₂O (1 mmol L⁻¹) for 5 min	UV-Vis, XRD, TEM, XRD, TGA	0.1, 0.2, 0.3, 0.5, 1, 2, 3, 4 µg mL⁻¹; Standard antibiotic ampicillin (0.01 mg mL⁻¹)	*Escherichia coli*,* Agrobacterium tumefaciens*,* Lactobacillus acidophilus*,* Staphylococcus aureus; Fungus: Trichoderma harzianum*	Disk diffusion; Luria-Bertani (LB) Agar	12 h	Nguyen et al., [89]
Cynodon *(Cynodon dactylon)*	10 g powder boiled with 100 mL distilled water for 1 h; extract and HAuCl₄·3 H₂O (1 mmol L⁻¹) ratios: 2:1, 5:1, 10:1, 20:1, 30:1, 2 h, 1200 rpm, 90 °C	UV-Vis, FT-IR, XRD, SEM, TEM	100, 75, 50, 25 µg mL⁻¹; Ciprofloxacin (100 µg mL⁻¹)	*Enterobacter cloacae*,* Staphylococcus haemolyticus*,* Staphylococcus petrasii subsp. pragensis*,* Bacillus cereus*	Disk diffusion; Mueller-Hinton Agar	24 h	Vinayagam et al., [97]
Black ginseng *(Panax Ginseng)*	5 g crushed roots in 50 mL H₂O, boiled at 60 °C for 20 min and cooled; extract and HAuCl₄·3 H₂O (1 mmol L⁻¹) ratios: 2:8, 5:5, 7:3, 8:2, incubated in sunlight for 2 h	UV-Vis, FE-TEM, EDX, XRD, FTIR	15, 30, 55 µg mL⁻¹	*Escherichia coli*,* Staphylococcus aureus*	Disk diffusion; Mueller-Hinton Agar	24 h	Wang et al., [234]
Gold Root *(Rhodiola rosea)*	5 mL ginseng in 25 mL sterile distilled water; 1:1 volumetric ratio (extract: HAuCl₄·3 H₂O 1 mmol L⁻¹), monitored at room temperature, 16,000 rpm for 15 min and air⁻dried	AFM, UV-Vis, FTIR, Zeta Potential, TEM, MALDI-TOF	6.25–400 µg mL⁻¹	*Pseudomonas aeruginosa and Escherichia coli biofilms*	MIC; Mueller-Hinton Broth	24 h	Singh et al., [53]
Shishiudo *(Angelica pubescens)*	10 g rhizome ground and boiled 30 min with 100 mL sterile water; 1:1 volumetric ratio (extract: HAuCl₄·3 H₂O 1 mmol L⁻¹), monitored at room temperature	UV-Vis, FE-TEM, EDX, XRD, SAED, DLS, FTIR	500, 1000, 1500 µg mL⁻¹; Neomycin 30 µg	*Escherichia coli*,* Staphylococcus aureus*,* Pseudomonas*	Disk diffusion; Mueller-Hinton Agar	24 h	Oh et al., [194]
Black ginseng *(Panax Ginseng)*	5 g root powder in 100 mL distilled water and autoclaved 30 min at 100 °C to obtain aqueous root extract. HAuCl₄·3 H₂O (1 mmol L⁻¹) at 1, 3, 5, 7, 9 mmol L⁻¹; extract solutions at 10, 30, 50, 70, 90% (v/v); temperatures 40–100 °C, pHs 2, 4, 6, 8, 12	UV-Vis, FE-TEM, EDX, XRD	100 µg mL⁻¹; novobiocin and lincomycin	*Bacillus anthracis*,* Vibrio parahaemolyticus*,* Bacillus cereus*	Disk diffusion; Mueller-Hinton Agar	24 h	Singh et al., [196]

* Thermogravimetric analysis (TGA), Gas chromatography-mass spectrometry (GC-MS/MS) and High performance liquid chromatography/ultraviolet-visible (HPLC/UV-VIS), Electron diffraction (SAED), X-ray diffraction (XRD), Dynamic light scattering (DLS), Energy dispersive spectroscopy (EDX), Energy dispersive spectroscopy (EDS), Fourier transform infrared spectroscopy (FTIR), Raman spectroscopy (Raman), Ultraviolet-visible spectroscopy (UV-Vis), Field emission splitting electron microscopy (FE-SEM), Transmission electron microscopy (TEM), High resolution transmission electron microscopy (HRTEM), Scanning electron microscopy (SEM), Zeta Potential (Zeta)

Curcumin-based AuNPs (from turmeric rhizomes) were effective in inhibiting both Gram-positive and Gram-negative bacteria, including *P. aeruginosa*, *S. aureus*, and *E. coli*, with inhibition rates exceeding 80% in the studies carried out by Kalantari et al. [91] and Mohammadi et al. [92] at concentrations of 1.84 mg mL⁻¹ and 14 mg mL⁻¹, respectively. Ginger (*Zingiber officinale*)-derived AuNPs were also tested by Kalantari et al. [91], Yadi et al. [71] and Chitra et al. [98] against the same bacterial strains, achieving inhibition at a concentration of 80 mg mL⁻¹.

Additional studies using ginger (*Zingiber officinale*), including those by Chitra et al. [98], Rajeshkumar et al. [99] and Velmurugan et al. [94]. also reported inhibition of *S. aureus* at 5 mg mL⁻¹, as well as effects against *K. pneumoniae* [98] with inhibition rates above 60% compared to standard antibiotics, and inhibition of Listeria spp [94]. and *Streptococcus mutans*, both at 20 µg mL⁻¹ [85].

Gold NPs synthesized using root extracts consistently demonstrated inhibitory effects against both Gram-positive and Gram-negative strains in all studies reporting antimicrobial activity. AuNPs from *Trillium govanianum* [93], African asparagus (*Asparagus racemosus*) [96], and lemon grass (*Cymbopogon citratus*) [100] inhibited *P. aeruginosa* by 66.6%, 75%, and 50% at 40 mg mL⁻¹, 80 µg mL⁻¹, and 0.25 mg mL⁻¹, respectively. The latter two also inhibited *S. aureus* by 62.5% and 80%, respectively, and the last demonstrated over 80% inhibition of biofilm growth.

The gold NPs synthesized from Neem (*Azadirachta indica*) at 5 mg·mL⁻¹ inhibited *Enterococcus faecalis* [85], while AuNPs from silk grass (*Cynodon dactylon*) [97] at 100 µg mL⁻¹ inhibited more than 60% of the growth of *Enterobacter cloacae*, *Staphylococcus haemolyticus*, *Staphylococcus petrasii* subsp. *pragensis*, and *Bacillus cereus*. Those synthesized from silk grass showed a significant increase in reactive oxygen species (ROS) generation, DNA fragmentation, and mitochondrial membrane alterations, as observed through assays using dichlorodihydrofluorescein diacetate (DCFH-DA), 4’,6-diamidino-2-phenylindole (DAPI), rhodamine-123, and acridine orange/ethidium bromide (AO/EtBr) staining [97]. Nanoparticle-induced damage to the bacterial cell wall, metabolic disruption, and DNA damage have been reported as potential antibacterial mechanisms [47]. The bactericidal efficacy is strongly influenced by nanoparticle size, high surface area-to-volume ratio, and shape. The AuNPs in question typically range from 12 to 25 nm in size, as confirmed by characterization studies [96], which facilitates their entry into bacterial cells [95].

Previous studies have shown that ginger (*Zingiber officinale*) extracts contain high levels of n-hexane, ethyl acetate, and Soxhlet-extractable compounds, including gingerol, shogaols, zingerone and paradol [101]. These compounds exhibit antibacterial activity and inhibit bacterial biofilm formation, explaining the broad inhibitory effects against both planktonic strains and biofilms [88].

A noteworthy study by Ganesan et al. [49] evaluated the antibacterial activity of AuNPs synthesized using rhizome extract from sweet flag (*Acorus calamus*), incorporated into cotton fabric for wound healing applications. This approach not only demonstrated antimicrobial activity but also promoted improved healing of infections caused by Gram-positive *S. aureus* and Gram-negative *E. coli*. The results showed that cotton fabrics coated with AuNPs exhibited superior antibacterial effects compared to those coated with pure extract or uncoated cotton [49].

The main bioactive chemical constituents identified in *A. calamus* roots include α- and β-asarone and isoasarone [102]. Hydroxyl groups present in gooseberry and cotton extracts may stabilize AuNPs on the cotton surface, which contains repeating units of 4-D-glucopyranose in its fibers [49]. This interaction may increase the surface area for enhanced AuNP adsorption and improve the functionality of the cotton-nanomaterial composite [85].

Regarding antimicrobial effects, the hydroxyl groups of glucose can lose protons (H⁺) due to affinity with nitrogen- and sulfur-containing compounds in bacterial cell walls [31]. Neutralization reactions may alter or disrupt membrane protein structures by binding to thiol and amino groups [39], as well as inhibit cellular respiration by interacting with sulfur-rich mitochondrial membranes [46] (Fig. 4, reaction II).

The study by Poojary et al. [90] on AuNPs synthesized from apricot (*Mammea suriga*) was the only one demonstrating significant efficacy against Gram-positive bacteria. Nanomaterials produced at a concentration of 3 mmol L⁻¹ were able to inhibit the growth of *B. subtilis* and *S. aureus* by more than 60%, suggesting that the apricot components incorporated into the AuNPs carry predominantly negative charges due to N–H, –C–C–C–, and –C–N groups and bonds. This mechanism resembles the effect observed with AuNPs synthesized from aqueous seed extract of bottlebrush (*Callistemon citrinus*) [62].

Studies involving the same AuNPs yield differing antimicrobial results, as illustrated by Wang et al. [31] and Singh et al. [53], who synthesized AuNPs using black ginseng (*Panax ginseng*). Wang reported inhibition rates above 65% at a nanoparticle concentration of 55 µg mL⁻¹ against *E. coli* and *S. aureus*, whereas Singh found no inhibitory effect at a higher concentration of 100 mg L⁻¹ for any tested strain. This discrepancy may be attributed to the lower effective concentration tested by the latter authors.

Another form of ginseng, ginseng berry (*Panax ginseng*), was tested against both Gram-positive and Gram-negative strains using fruit extract as the reducing agent [103]. Significant antimicrobial effects were observed, indicating that different parts of the ginseng plant can be exploited for AuNP synthesis and their potential antimicrobial applications.

### Gold nanoparticles derived from leaf extracts

Leaf extracts have been widely used historically in the form of teas and mixtures to treat various ailments [104]. Herein, 81 studies were retrieved on AuNPs from plant leaves, with 15 reporting success in inhibiting Gram-positive bacteria (Table 3).

**Table 3:** Studies on the synthesis of green gold nanoparticles from leaf extract, including plant species, synthesis methods, characterization techniques, applied concentrations, bacterial strains tested, exposure time, and authors

Species	Synthesis Method	NP Characterization	NP Concentrations	Description of Bacteria	Experimental Design and Culture Medium	Exposure Time	References
Jamelão *(Syzygium cumini)*	10 g of leaves in distilled water and filtered; salt and plant extract concentration, temperature, reaction time, and pH	UV-Vis, TEM, XRD, and FT-IR	100, 150, 250, 350, 450, and 500 µg mL⁻¹	*Staphylococcus aureus*,* Acinetobacter baumannii*,* Escherichia coli*,* Pseudomonas aeruginosa*,* Enterococcus faecalis*,* Klebsiella pneumoniae*,* and Proteus vulgaris*	Disk diffusion; Mueller-Hinton Agar. Minimum Inhibitory Concentration (MIC); Luria-Bertani (LB) broth	24 h	Diksha et al., [56]
Graviola *(Annona muricata)*	20 g of leaves in 150 mL deionized water, boiled for 20 min, and filtered; 1 mL of extract added to 10 mL HAuCl₄ 1 mmol L⁻¹, stirred at room temperature until color change	UV-Vis, TEM, and FT-IR	2 and 4 mg L⁻¹	*Staphylococcus aureus*,* Enterococcus faecalis*,* Klebsiella pneumoniae*,* Clostridium sporogenes*	Disk diffusion; Nutrient Agar	28 h	Folorunso et al., [54]
Maple *(Acer pentapomicum)*	15–20 g of dried leaf powder in 150 mL deionized water, boiled 20 min, filtered; 1 mL of aqueous leaf extract with varying proportions of 1 mmol L⁻¹ gold chloride solution for 24 h	UV-Vis, TEM, XRD, and SEM	1 and 2 mg µL⁻¹	*Staphylococcus aureus*,* Pseudomonas aeruginosa*,* Klebsiella pneumoniae*,* Xanthomonas*,* Escherichia coli*,* Citrobacter freundi*,* Bacillus subtilis*	Disk diffusion; Nutrient Agar	24 h (bacteria); 48–96 h (fungi)	Khan et al., [7]
Papaya *(Carica papaya)*	20 g extracted using Soxhlet with 150 mL sterile distilled water; 1 mmol L⁻¹ HAuCl₄ with varying extract concentrations (CP, CR, CPCRM) 5–200 µL at 60 °C for 5 min	HRTEM and FTIR	10 mg mL⁻¹ of each solution	*Staphylococcus aureus*,* Escherichia coli*,* Bacillus subtilis*,* Proteus vulgaris*	Disk diffusion; Mueller-Hinton Agar. MIC; Mueller-Hinton broth	24 h	Muthukumar et al., [25]
Castor *(Ricinus communis)*	1 g dry substrate in 100 mL 70% acetone (stock solution 1%); 1 mL HAuCl₄·3 H₂O 1 mmol L⁻¹ with 99 mL stock solution of 1% extract	Extract: GC-MS/MS chromatography, HPLC/UV-Vis; RcExt-AuNPs: UV-Vis, SEM, and FT-IR	100 µg mL⁻¹	*Strains: Escherichia coli*,* Proteus mirabilis*,* Shigella flexneri*,* Staphylococcus aureus; Fungus: Candida albicans*	Disk diffusion; Nutrient Agar	24 h	Ghramh et al., [26]
Midnapore Creeper *(Rivea hypocrateriformis)*	10 g plant material in 200 mL distilled water, microwave irradiated (700 W, 2.45 GHz); 20 mL extract added to 50 mL 1 mmol L⁻¹ HAuCl₄ solution and similarly irradiated	UV-Vis, TEM, XRD, FT-IR, and EDAX	25, 50, 75, 100 µg mL⁻¹	*Strains: Klebsiella pneumoniae*,* Staphylococcus aureus*,* Bacillus subtilis*,* Pseudomonas aeruginosa*,* Escherichia coli; Fungi: Candida albicans*,* Trichophyton rubrum*,* Chrysosporium indicum*	Disk diffusion; Mueller-Hinton Agar	24 h	Godipurge et al., [240]
Indian jujube *(Ziziphus zizyphus)*	10 g leaves in 100 mL bidistilled water, boiled 5 min; 5 mL extract added to 45 mL HAuCl₄ 1 mmol L⁻¹ until color change	TEM, SEM, AFM, XRD, UV-Vis, EDX, and TGA	10, 25, 50, 100, 250, 500, 1000 µg mL⁻¹	*Klebsiella pneumoniae*,* Staphylococcus aureus*,* Bacillus subtilis*,* Pseudomonas aeruginosa*,* Escherichia coli*,* and X compstri*	Disk diffusion; Mueller-Hinton Agar	24 h	Aljabali et al., [3]
Princess ear *(Pergularia daemia)*	10 g dried leaves in 100 mL deionized water; leaf extracts obtained at 0, 30, 60, 90, 120 min, treated with 100 mL 1 mmol L⁻¹ HAuCl₄ solution	HRTEM, FESEM, XRD, UV-Vis	100, 200, 300 µL (1 mg mL⁻¹)	*Escherichia coli*,* Pseudomonas aeruginosa*,* Bacillus subtilis*	Disk diffusion; Mueller-Hinton Agar	48 h	Rajendran et al., [105]
Starfruit *(Terminalia arjuna)*	10 g fruit in 100 mL bidistilled water, boiled 50–60 °C for 5 min; 1 mL extract added to 100 mL 1 mmol L⁻¹ HAuCl₄ at room temperature for 15 min	UV-Vis, TEM, EDX	100 µL (20 mg mL⁻¹)	*Staphylococcus aureus*,* Pseudomonas aeruginosa*,* Salmonella typhimurium*	Disk diffusion; Nutrient Agar	24 h	Dudhane et al., [11]
Carnivorous plant *(Nepenthes khasiana)*	20 g in 200 mL autoclaved bidistilled water, boiled 30 min, filtered; 25 mL extract added to 225 mL 1 mmol L⁻¹ gold chloride solution for 3 h under gentle stirring	UV-Vis, TEM, SEM, XRD, FT-IR	5 mg mL⁻¹	*Strains: Escherichia coli*,* Bacillus species; Fungi: Aspergillus niger*,* Candida albicans*	Disk diffusion; Nutrient Agar	24 h (bacteria); 48 h (fungi)	Bhau et al., [106]
Desert rose *(Justicia glauca)*	1 g fresh leaves boiled 15 min in 300 mL sterile distilled water; 1 mmol L⁻¹ HAuCl₄·3 H₂O mixed 1:1 with extract at room temperature for 10 min	UV-Vis, TEM, SEM, XRD, FT-IR, EDAX	50, 25, 12.5, 6.25, 3.125, 1.56, 0.78 µg mL⁻¹; standard antibiotics azithromycin and clarithromycin 100 µg mL⁻¹	*Bacteria: Micrococcus luteus*,* Bacillus subtilis*,* Staphylococcus aureus*,* Streptococcus mutans*,* Lactobacillus acidophilus*,* Escherichia coli*,* Pseudomonas aeruginosa; Fungi: Saccharomyces cerevisiae*,* Candida albicans*	Minimum inhibitory concentration; Nutrient broth	24 h	Emmanuel et al., [1]
Camellia *(Camellia japonica L.)*	5 g finely cut dried leaves with 200 mL water for 10 min, filtered; 10 mL extract with 50 mL 0.5 mmol L⁻¹ gold solution, stirred at room temperature 40 min	UV-Vis, TEM, SEM, XRD, FT-IR	100, 150, 200 µg mL⁻¹	*Strains: Bacillus subtilis*,* Staphylococcus aureus*,* Streptococcus faecalis*,* Klebsiella pneumoniae*,* Pseudomonas aeruginosa*,* Escherichia coli; Fungus: Candida albicans*	Disk diffusion; Nutrient Agar	24 h	Sharma et al., [2]
Chita plant *(Alternanthera bettzickiana)*	10 g fine powder in 100 mL bidistilled water at 80 °C for 10 min in water bath; 5–20 mL of aqueous gold solution heated to 80 °C for 10 min	UV-Vis, TEM, SEM, XRD, FT-IR, EDX	10, 20, 30, 40 µL (20 mg mL⁻¹)	*Bacillus subtilis*,* Staphylococcus aureus*,* Salmonella typhi*,* Pseudomonas aeruginosa*,* Micrococcus luteus*,* Enterobacter aerogenes*	Disk diffusion; Nutrient Agar	24 h	Nagalingam et al., [107]
Dandelion *(Tragopogon dubius)*	10 g in 100 mL distilled water, boiled 5 min; 1 mL leaf extract added to 10 mL 1 mmol L⁻¹ HAuCl₄ solution at room temperature for 24 h	UV-Vis, TEM, SEM, XRD, FT-IR	2.5, 5, 7.5, 10 mg mL⁻¹	*Klebsiella pneumoniae*,* Bacillus cereus*,* Escherichia coli*,* Staphylococcus aureus*	Disk diffusion; Nutrient Agar	24 h	Layeghi-ghalehsoukhteh et al., [108]
Oregano *(Origanum vulgare)*	2 g with 20 mL water for 30 min at 60 °C; 20 mL HAuCl₄ (1 mmol L⁻¹) with 0.25–2 mL plant extract at room temperature until color change	UV-Vis, TEM, FT-IR, Raman	1.23, 2.4, 4.7, 6.9, 9 mg mL⁻¹	*Staphylococcus aureus*,* Listeria monocytogenes*,* Escherichia coli*,* Salmonella typhimurium*	Disk diffusion; Mueller-Hinton Agar	Overnight	Benedec et al., [70]
Wild fennel *(Nigella arvensis)*	10 g powder in 200 mL boiling water for 15 min, filtered; 10 mL extract with 90 mL 1 mmol L⁻¹ HAuCl₄ solution until color change	UV-Vis, TEM, XRD, FT-IR	100 µL (disk), 50 µL (MIC) (5 mg mL⁻¹)	*Staphylococcus epidermidis*,* Bacillus subtilis*,* Staphylococcus aureus*,* Escherichia coli*,* Serratia marcescens*,* Pseudomonas aeruginosa*	Disk diffusion; Mueller-Hinton Agar; MIC (Mueller-Hinton broth)	24 h	Chahardoli et al., [109]
Sambacaetá *(Dracocephalum kotschyi)*	10 g fine powder in 200 mL water, boiled 15 min; 10 mL extract with 90 mL 1 mmol L⁻¹ HAuCl₄ solution for 10 min at room temperature	UV-Vis, TEM, FESEM, XRD, FT-IR, EDX	50 µL (5 mg mL⁻¹)	*Staphylococcus epidermidis*,* Bacillus subtilis*,* Staphylococcus aureus*,* Escherichia coli*,* Serratia marcescens*,* Pseudomonas aeruginosa*	MIC; Nutrient broth	24 h	Chahardoli et al., [109]
Starfruit *(Terminalia arjuna)*	10 g leaves in 100 mL bidistilled water, boiled 50–60 °C for 5 min, filtered; 1 mL extract with 100 mL 1 mmol L⁻¹ HAuCl₄ at room temperature for 15 min	UV-Vis, TEM, XRD, FT-IR, DLS, Zeta Potential	500 µmol mL⁻¹, 1000 µmol mL⁻¹ (1 mg mL⁻¹)	*Staphylococcus aureus*,* Klebsiella pneumoniae*,* Proteus vulgaris*	Disk diffusion; Nutrient Agar	24 h	Gopinath et al., [11]
Glory tree *(Clerodendrum trichotomum)*	15 g powdered leaves combined with 100 mL water at 50–60 °C for 15 min; 10–20 g HAuCl₄ in 100 mL extract for green synthesis of AuNPs (Au³⁺ concentration 300 mmol L⁻¹), stirred 80 min at 65 °C	TEM, XRD, EDX	666 µg mL⁻¹	*Klebsiella pneumoniae*,* Staphylococcus aureus*	MIC; Mueller-Hinton broth	—	Shakoor et al., [110]
Wild Rue *(Peganum harmala L.)*	4 g of powder in 400 mL distilled water for 2 weeks. Volumetric ratios of 2:1, 3:1, 4:1, 6:1, 7:1 (HAuCl₄ 1 mmol L⁻¹ : extract) at room temperature until color change	UV-Vis, TEM, XRD, FT-IR	5; 3.3; 2.5; 1.6; 1.43 µg mL⁻¹	*Bacteria: Bacillus subtilis*,* Staphylococcus aureus*,* Escherichia coli*,* Pseudomonas aeruginosa*,* Salmonella typhi; Fungi: Candida albicans*,* Aspergillus niger*,* Penicillium notatum*	Disk diffusion; Nutrient agar	24 h	Ullah et al., [242]
Tiger Claw *(Martynia annua)*	1 g leaves in 100 mL water; 5 mL extract with 5 mL of 1 mmol L⁻¹ HAuCl₄ in dark until color change	UV-Vis, SEM, XRD, DLS, Zeta Potential	6.25, 12.5, 25, 50, 100 µg mL⁻¹	*Escherichia coli*,* Staphylococcus aureus*,* Streptococcus sp*,* Bacillus subtilis*,* Enterococcus faecalis*	Minimum Inhibitory Concentration (MIC); Mueller-Hinton broth	20 h	Palaniswamy et al., [111]
Geranium *(Pelargonium graveolens)*	Volume of HAuCl₄ added to 50 mL extract to reach 250 µg mL	UV-Vis, TEM, FESEM, XRD, FT-IR, EDS	0.2 mL of agent in concentrations 0.06, 0.12, 0.25, 0.5 mg mL⁻¹	*Bacteria: Streptococcus mutans; Fungi: Candida albicans*	Agar well diffusion; Mueller-Hinton	24 h	Asker et al., [112]
Sapodilla *(Manilkara zapota)*	6 mL de HAuCl₄·3 H₂O em 1 mL de extrato aquoso agitada no escuro por 10 minutos à temperatura ambiente. A cor da solução mudou imediatamente para púrpura.	UV-Vis	50, 33.3, 1.67 mg mL⁻¹	*Escherichia coli*,* Salmonella typhi*,* Staphylococcus aureus*,* MRSA*	Agar well diffusion; Mueller-Hinton	24 h	Hutagalung et al. [167]
Sweet Violet *(Viola odorata)*	1:1 to 1:50 (salt: extract 0.3 g mL), 1 h at 100 °C	UV-Vis, SEM	300 mg mL⁻¹	*Bacteria: Staphylococcus aureus*,* Salmonella typhimurium*,* Pseudomonas aeruginosa*,* Escherichia coli; Fungi: Candida albicans*,* Aspergillus niger*	Agar well diffusion; Nutrient agar	24 h	Ridwansyah et al., [113]
Blackberry *(Rubus spp.)*	460 g mol L⁻¹ extract with HAuCl₄·3 H₂O 1 mmol L⁻¹, 1:1 molar ratio at room temperature until color change	UV-Vis, SEM, XRD, EDS, DLS, Zeta Potential	Same as synthesis	*Bacillus subtilis*,* Staphylococcus aureus*,* Escherichia coli*,* Proteus vulgaris*,* Pseudomonas aeruginosa*,* Klebsiella pneumoniae*,* Listeria monocytogenes; Candida albicans; Amoxicillin + Clavulanic acid 20/10 µg*	Disk diffusion; Mueller-Hinton agar	24 h	Tasić et al., [114]
Suren *(Toona sureni (Blume) Merr.)*	10 mg mL⁻¹ extract in HAuCl₄ 0.1 mmol L⁻¹, ratio 1:19 (v/v); 900 W microwave irradiation for heating	UV-Vis, TEM, FESEM, XRD, FT-IR, EDS, DLS, Zeta Potential	100 µL (15.6–62.5 µg mL⁻¹)	*Staphylococcus epidermidis*,* Propionibacterium acnes*,* Staphylococcus aureus*,* Escherichia coli*,* Pseudomonas aeruginosa MRSA*	MIC; Brain Heart Infusion (BHI)	24 h	Lestari et al. [157]
Castor *(Ricinus communis)*	20 g leaves boiled with 200 mL distilled water at 70 °C for 1 h; 2 mL extract to 18 mL of 1 mmol L⁻¹ HAuCl₄·3 H₂O for 15 min	UV-Vis, TEM	11 mg mL⁻¹	*Bacteria: Bacillus subtilis*, Pseudomonas aeruginosa; Fungi: Candida albicans*,* Aspergillus fumigatus*	MIC; Mueller-Hinton broth	24 h	Parmar et al., [168]
Dancing Plant *(Codariocalyx motorius)*	2 g leaves in 100 mL water, heated and filtered; 20 mL of 1 mmol L⁻¹ HAuCl₄ solution in 2:8 ratio (v/v) with extract; dried at 100 °C for 10 min	UV-Vis, TEM, FESEM, XRD, FT-IR, EDS, DLS, Zeta Potential	10 µL (125 µg mL⁻¹)	*Bacteria: Escherichia coli*,* Staphylococcus aureus; Fungi: Aspergillus flavus*,* Candida albicans*	Disk diffusion; Mueller-Hinton agar	24 h	Deivanathan et al., [155]
Sugar Apple *(Annona squamosa L.)*	5 g leaves in 100 mL deionized water at 60 °C for 10 min, filtered; 5 mL to 25 mL of 1 mmol L⁻¹ HAuCl₄ at room temperature, 350 rpm, 20 h	TEM, UV-Vis, XRD, FT-IR, DLS	1.953, 3.906, 7.8125, 15.625, 31.25, 62.5, 125, 250, 500, 1000, 2000 µg mL⁻¹	*Escherichia coli*,* Staphylococcus aureus*	MIC; Mueller-Hinton broth	overnight	Jibrin et al., [115]
Caribbean Jasmine *(Plumeria pudica)*	20 g chopped leaves with 200 mL deionized water, heated 105 °C for 2 h; different volumes of 0.5–2.5 mL HAuCl₄ solution in 5 mL extract; 50 °C, 20 min	UV-Vis, TEM, XRD, FT-IR, EDS, PL, Zeta Potential	0.1; 0.2; 0.3; 0.4; 0.5 mg mL⁻¹	*Bacillus subtilis*,* Escherichia coli*,* Pseudomonas aeruginosa*	Disk diffusion; Mueller-Hinton agar	overnight	Anamika et al., [116]
Mozambique Flower *(Elytraria acaulis)*	5 g leaf in 250 mL deionized water at 60 °C for 20 min; 34 mL of 1 mmol L⁻¹HAuCl₄ aqueous solution with 3 mL extract incubated overnight	UV-Vis, TEM, XRD, FT-IR, FESEM, PL	10 mg mL⁻¹	*Escherichia coli*,* Staphylococcus aureus*	Disk diffusion; Mueller-Hinton agar	18 h	Sathiyaraj et al., [117]
King of Bitters *(Andrographis paniculata)*	10 g powdered leaves in 100 mL water; 10 mL extract added to 90 mL HAuCl₄; pH 7.0, 70 °C until color change	TEM, UV-Vis, XRD, FT-IR	10 mg mL⁻¹	*Salmonella typhi*,* Bacillus cereus*,* Pseudomonas aeruginosa*,* Escherichia coli*	Disk diffusion; Mueller-Hinton agar	overnight	Chandran et al. [161]
Showy Violet *(Viola betonicifolia)*	20 g leaves in 150 mL water; 1 mmol L⁻¹ HAuCl₄·3 H₂O added to 25 mL leaf extract at 40 °C for 60 min	TEM, UV-Vis, XRD, FT-IR, EDS, DLS	250 µg mL⁻¹	*Bacillus subtilis*,* Staphylococcus aureus*,* Escherichia coli*,* Pseudomonas aeruginosa*	Disk diffusion; TSB & RPMI agar	24 h	Wang et al., 2021
Chinese Bellflower *(Platycodon grandiflorus)*	10 g dried leaves in 100 mL distilled water; 30 mL of 1 mmol L⁻¹ HAuCl₄·3 H₂O with 10 mL extract, incubated at different temperatures (20, 37, 50 °C); optimized with 2.5, 5, 10 mL extract at 50 °C	TEM, UV-Vis, XRD, FT-IR, EDS, DLS	5, 10, 15, 20 µg mL⁻¹	*Escherichia coli*,* Bacillus subtilis*	Disk diffusion; LB agar	24 h	Anbu et al., [118]
White Wormwood and Mulberry *(Artemisia herba-alba and Morus alba)*	10 g powder in 100 mL; 10 µL extract with 90 µL of 1 mmol L⁻¹ HAuCl₄ at 25 °C for 20 min	UV-Vis, SEM, TEM	10, 20, 40, 60, 80 µg mL⁻¹	*Escherichia coli*,* Salmonella typhi*	Agar well diffusion; Mueller-Hinton	24 h	Abdalhamed et al., [119]
Paradise Tree *(Simarouba glauca)*	1 g powdered leaves in 50 mL distilled water, stirred 1 h; extract: salt ratios 0.3:27, 0.6:24, 0.9:21, 1.2:1.8 at room temperature until color change	TEM, UV-Vis, XRD, FT-IR, EDS	2, 4, 6, 8 mg mL⁻¹	*Staphylococcus aureus*,* Streptococcus mutans*,* Bacillus subtilis*,* Escherichia coli*,* Proteus vulgaris*,* Klebsiella pneumoniae*	Disk diffusion; Mueller-Hinton agar	24 h	Thangamani et al., [120]
Mast Tree *(Polyalthia longifolia)*	10 g dried green leaves in 100 mL boiling water; 10 mL of extract added to 100 mL aqueous solution of 1 mmol L⁻¹ HAuCl₄ ×H₂O at room temperature, stirred for 20 min	TEM, SEM, UV-Vis, XRD, FT-IR, EDS	100 µg mL⁻¹ each	*Escherichia coli*,* Bacillus subtilis*	Disk diffusion; Mueller-Hinton agar	24 h	Mumtaz et al., [121]
Gundelia *(Gundelia tournefortii L.)*	10 g dried green leaves in 100 mL boiling water; 50 mL extract + 75 mL HAuCl₄·3 H₂O 3 mmol L⁻¹ at 45 °C until color change	FT-IR, FESEM, EDS, UV-Vis	AuNPs 40 mg mL⁻¹; fluconazole (fungus), vancomycin (Gram+), colistin (Gram-) 128 mg mL⁻¹	*Bacteria: Escherichia coli*,* Pseudomonas aeruginosa*,* Bacillus subtilis*,* Staphylococcus aureus; Fungi: Candida albicans*	MIC; BHI	24 h	Keskin et al., [122]
Sambacaetá *(Dracocephalum kotschyi)*	5 g leaves boiled with 75 mL deionized water; 10 mL HAuCl₄ 1 mmol L⁻¹ added to 2 mL extract, stirred vigorously for 2 min	EM-SEAD, SEM-EDAX, XRD, Zeta Potential, DLS, FT-IR	11 mg mL⁻¹	*Bacillus subtilis*,* Staphylococcus aureus*,* Bacillus cereus*,* Escherichia coli*,* Pseudomonas aeruginosa*,* Proteus vulgaris*	Agar well diffusion; Mueller-Hinton	24 h	Dorosti; Jamshidi, [123]
Garlic *(Allium noeanum)*	40 g in distilled water for 48 h at 30 °C; 10 mL of extract added to 100 mL aqueous solution of 1 mmol L⁻¹ HAuCl₄ × H₂O at room temperature, stirred for 30 min; dried in oven at 50 °C	FT-IR, UV, XRD, EDS, FE-SEM, TEM	2, 4, and 8 mg mL⁻¹	*Streptococcus pneumoniae*,* Bacillus subtilis*,* Staphylococcus aureus*,* Staphylococcus saprophyticus*,* Salmonella typhimurium*,* Pseudomonas aeruginosa*,* Shigella flexneri*,* Escherichia coli*	Disk diffusion; Mueller-Hinton agar	24 h	Shahriari et al., [124]
Arabian Jasmine *(Jasminum sambac)*	10 g leaves in 200 mL distilled water; 10 mL extract to 50 mL of 1 mmol L⁻¹ aqueous HAuCl₄; microwave irradiation at maximum power (700 W, 2.45 GHz); vacuum oven at 70–80 °C for ~ 12 h to dry	XRD, EDX, FESEM, FT-IR	1 mg mL⁻¹	*Staphylococcus aureus*,* Salmonella typhimurium*,* Pseudomonas aeruginosa*,* Escherichia coli*	Disk diffusion; Mueller-Hinton agar	24 h	Yallappa et al., [125]
Qinghaosu *(Artemisia annua)*	4 g leaves in 200 mL deionized water, boiled for 5 min; 5 mL extract added to 50 mL saline solutions (5 mmol L⁻¹), stirred 10 min at room temperature; dried at 40 °C for 2 h	UV-Vis, XRD, TEM, EDX, FT-IR, TGA	1000, 100, 10, 1 µg mL⁻¹	*Escherichia coli*,* Pseudomonas aeruginosa*,* Enterobacter aerogenes*,* Bacillus cereus*,* Staphylococcus aureus*	Disk diffusion; Mueller-Hinton agar	24 h	Basavegowda et al., [195]
Boldo *(Coleus forskohlii)*	100 mg in 100 mL Milli-Q water, filtered; 1–5 mL extract added to 10 mL 1 mmol L⁻¹ HAuCl₄ at 80 °C for 15 min	UV-Vis, XRD, TEM, FT-IR	15, 25, 35 µL (0.5 mg mL⁻¹)	*Escherichia coli*,* Pseudomonas aeruginosa*,* Staphylococcus aureus*	Disk diffusion; Mueller-Hinton agar	24 h	Naraginti et al., [126]
Olive *(Olea europaea)*	10 g powdered leaves in 100 mL distilled water, boiled 5 min, filtered; 25 mL extract added to 1 mmol L⁻¹ HAuCl₄·3 H₂O, heated at 80 °C for 65 min; centrifuged 15 min at 15,000 rpm, washed thrice with deionized water, dried at 70 °C	UV-Vis, XRD, TEM, EDX	500 µg/mL; ciprofloxacin	*Staphylococcus aureus*,* Klebsiella pneumoniae*	Agar well diffusion; Mueller-Hinton agar	24 h	Sellami et al., [127]
King of bitters *(Andrographis paniculata)*	50 mL 1 mmol L⁻¹ HAuCl₄ solution heated at 80 °C for 10 min, stirred 15 min at 90 °C, 5 mL leaf extract (10% w/v) added dropwise; dried at 50 °C	UV-Vis, SEM, TEM, FT-IR	50, 100 µg mL⁻¹	*Escherichia coli*,* Staphylococcus aureus*,* Enterococcus faecalis; Fungi: Aspergillus niger*,* Candida albicans*	Agar well diffusion; Mueller-Hinton agar	24 h	Paramasivam et al., [128]
Bird Plum *(Sageretia thea)*	500 g of leaf powder was in 1500 mL of ethanol for 2 weeks. 1 mL of leaf extract solution with 1 mmol L⁻¹ of HAuCl₄ in different proportions (1:1 to 1:10) in volume and stirred for 30 min at room temperature until color change	UV-Vis, XRD, TEM, SEM	100, 200 µg mL⁻¹	*Klebsiella pneumoniae*,* Staphylococcus aureus*,* Bacillus subtilis*	Agar well diffusion; Mueller-Hinton agar	24 h	Shah et al., [40]
Parsley *(Petroselinum crispum)*	20 g dried leaves in 200 mL deionized water at 90 °C for 20 min; 2.5, 5, 10, 20 mL extract (AuNPs A–D), keeping HAuCl₄·H₂O molarity 1 mmol L⁻¹ until color change	UV-Vis, XRD, TEM, EDX	200 µg mL⁻¹	*Bacillus subtilis*,* Enterococcus faecalis*,* Escherichia coli*,* Enterobacter ludwigii*	Agar well diffusion; Mueller-Hinton agar	48 h	El-borady et al., [129]
Wheat *(Triticum aestivum)*	20 g chopped leaves in 100 mL HPLC purified water; 10 mL hot extract added to 90 mL 1 mmol L⁻¹ HAuCl₄.XH₂O; boiled 10 min at 90 °C	UV-Vis, FT-IR, DLS, XRD, HRTEM	0.5, 1.0, 2.0, 3.0 mg mL⁻¹	*Klebsiella pneumoniae*,* Salmonella typhimurium*,* Enterobacter aerogenes*,* Escherichia coli*,* Micrococcus luteus*,* Staphylococcus aureus*,* Staphylococcus epidermidis*,* Streptococcus mutans*	Agar well diffusion; Mueller-Hinton agar	24 h	Pahal et al., [130]
Almonds *(Amygdalus communis)*	10 g in 100 mL water; 30 mL extract added to 120 mL HAuCl₄ (20 mmol L⁻¹) at 1:4 ratio; 10, 15, 30, 45, 60 min at room temperature	Extract: LC-ESI-MS/MS; NPs: XRD, TEM, FT-IR, FESEM, UV-Vis	5–0.156 mg mL⁻¹	*Staphylococcus aureus*,* Bacillus subtilis*,* Escherichia coli*,* Pseudomonas aeruginosa; Fungi: Candida albicans*	MIC in BHI	24 h	Baran et al., [131]
Imperial cassia *(Cassia fistula)*	100 mL of deionized water was poured over 15 g of cut leaves and boiled at 90 °C for 45 min and filtered. 10 mL of HAuCl₄0.3 H₂O aqueous solution (1 mmol L⁻¹) was mixed dropwise with 10 mL of extract and shaken vigorously at 450 rpm for 6 min until color change.	UV-Vis, TEM, EDX, FT-IR, DLS and Zeta Potential	0,15 mg mL⁻¹	*Escherichia coli*	MIC; Mueller-Hinton broth	24 h	Maji et al., [152]
Albizia *(Albizia amara Roxb.)*	1 g leaf powder in 100 mL deionized water, boiled 30 min, filtered; 8 mL aqueous extract to 60 mL HAuCl₄ 1 mmol L⁻¹, orbital shaker at room temperature; overnight drying	FESEM, EDX, XRD, FT-IR, UV-Vis	20, 40, 60, 80, 100 µg mL⁻¹	*Staphylococcus aureus*	Agar well diffusion; Mueller-Hinton agar	24 h	Balasubramani et al., [132]
Bee Sting Bush *(Azima tetracantha Lam.)*	20 g fine powder in 200 mL Milli-Q water, boiled 10 min at 60 °C; 10 mL extract with 90 mL HAuCl₄ (1 mmol L⁻¹) at 60 °C for 10 min	DLS, SEM, UV-Vis, FT-IR, XRD	15, 30 µL (10 mg mL⁻¹)	*Aeromonas liquefaciens*,* Enterococcus faecalis*,* Micrococcus luteus*,* Salmonella typhimurium; Fungi: Candida albicans*,* Cryptococcus sp.*,* Microsporum canis*,* Trichophyton rubrum*	Agar well diffusion; Mueller-Hinton agar	24 h	Hariharan et al., [133]
Pecah beling *(Strobilanthes crispa)*	2 g leaf powder in 100 mL deionized water, boiled 15 min at 100 °C; 10 mL HAuCl₄ (1 mmol L⁻¹) with 5 mL extract at room temperature for 1 h	UV-Vis, FT-IR, HRTEM, XRD	6.6 g mL⁻¹	*Escherichia coli*,* Staphylococcus aureus*	Agar well diffusion; Mueller-Hinton agar	24 h	Samsulkahar et al., [241]
River Willow *(Combretum erythrophyllum)*	5 g dried leaves in 100 mL deionized water at 90 °C for 1 h; different extract volumes (0.5, 1.0, 1.5, 2.0 mL) to 25 mL HAuCl₄ 1 mmol L⁻¹ at room temperature until color change	UV-Vis, FT-IR, HRTEM, XRD	2000, 1000, 500, 250, 125, 62.5 µg mL⁻¹	*Staphylococcus epidermidis*,* Staphylococcus aureus*,* Mycobacterium smegmatis*,* Proteus mirabilis*,* Escherichia coli*,* Klebsiella pneumoniae*,* Klebsiella oxytoca*	MIC; Mueller-Hinton broth	24 h	Fanoro et al., [134]
Smoke Tree *(Cotinus coggygria Scop.)*	20 g chopped plant material extracted with 350–400 mL ethanol for 3 h using magnetic stirrer at room temperature; different volumes (0.1, 0.2, 0.5 mL) of aqueous extract added to chloroauric acid solution, final volume 25 mL, stirred at 250 rpm, 25 °C until color change	UV-Vis, FT-IR, Fluorescence, TEM	30 µL (0.2 mg mL⁻¹)	*Escherichia coli*,* Staphylococcus aureus*	Agar well diffusion; Mueller-Hinton agar	24 h	Ozgur et al., [135]
Chinese Mulberry *(Cudrania tricuspidata)*	30 g leaves in 100 mL deionized water, boiled 30 min, cooled; 100 mL extract added to 1 L (1 mmol L⁻¹) HAuCl₄ at room temperature until color change	UV-Vis, FT-IR, HRTEM, SAED, EDS, XRD	50, 100, 150, 200 mg mL⁻¹	*Bacillus subtilis*,* Pseudomonas aeruginosa*	Agar well diffusion; Mueller-Hinton agar	24 h	Velmurugan et al., [136]
Christmas Box *(Sarcococca saligna)*	10 g fine powder in 50 mL distilled water for ~ 6 h at 40 °C; 5 mL extract to 95 mL HAuCl₄ 1 mmol L⁻¹	UV-Vis, TEM	10 mg mL⁻¹	*Candida albicans; Staphylococcus aureus*,* Escherichia coli (comparable to amoxicillin and fluconazole)*	Agar well diffusion; Nutrient agar	24 h	Rehman et al., [137]
Saltwort *(Cressa cretica)*	10 g leaf powder in 100 mL at 80 °C for 20 min, filtered; 10 mL extract with 90 mL HAuCl₄·3 H₂O (1 mmol L⁻¹) at room temperature for 2 h	UV-Vis, FT-IR, TEM, SEM, XRD, EDX	1, 2, 3 mg mL⁻¹; Chloramphenicol 10 µg	*Streptococcus pyogenes*,* Staphylococcus aureus*,* Escherichia coli*,* Klebsiella pneumoniae*	Agar well diffusion; Mueller-Hinton agar	24 h	Balasubramanian et al., [85]
Kalmegh *(Andrographis paniculata)*	4 g of leaves in 250 mL of water at 50 °C, filtered; 1.5 mL of extract in 0.5 mmol L⁻¹ HAuCl₄, 60 °C for 30 min, pH 6	UV-Vis, FTIR, TEM, XRD, EDX	0.75 mg/mL	*Escherichia coli*,* Salmonella enterica*,* Bacillus subtilis subsp. spizizenii*,* Staphylococcus aureus*	Disc diffusion; Mueller-Hinton agar	24 h	Do Dat et al., [138]
*Black nightshade (Solanum nigrum)*	10 g in 100 mL distilled water at 100 °C for 30 min; HAuCl₄ (12 mL, 0.3 mmol L⁻¹) to 8 mL extract with constant stirring for 10 min until purple	UV-Vis, FTIR, TEM, XRD, EDAX	50 µg mL⁻¹	*Escherichia coli*	Disc diffusion; Mueller-Hinton agar	24 h	Vijilvani et al., [139]
Samar tree *(Haloxylon salicornicum)*	2 g powder in 100 mL distilled water, boiled at 80 °C in a water bath for 15 min; 1 mL plant extract with 9 mL 1 mmol L⁻¹ HAuCl₄·3 H₂O at 80 °C for 30 min until reddish-pink color stabilized	UV-Vis, FTIR, XRD, Zeta potential	2 mg mL⁻¹	*Staphylococcus aureus*,* Bacillus cereus*,* Escherichia coli*,* Klebsiella pneumoniae*	Minimum Inhibitory Concentration (MIC); Mueller-Hinton broth	24 h	Hamida et al., [140]
Wild jasmine *(Clerodendrum inerme)*	10 g leaf powder in 100 mL deionized water; 25 mL extract with 1 mmol L⁻¹ HAuCl₄·3 H₂O at 80 °C for 65 min, dried at 70 °C after 15,000 rpm for 15 min	UV-Vis, FTIR, TEM, XRD, DLS	250 µg mL⁻¹ (well); 40–0.781 µg mL⁻¹ (broth)	*Bacillus subtilis*,* Staphylococcus aureus*,* Klebsiella*,* Escherichia coli; Fungi: Aspergillus niger*,* Trichoderma harzianum*,* Aspergillus flavus*	Well diffusion; Mueller-Hinton agar; MIC; Mueller-Hinton broth	24 h	Khan et al., [141]
Orchid tree *(Bauhinia purpurea)*	25 g boiled for 20 min with 100 mL bidistilled water; extract and HAuCl₄ (1 mmol L⁻¹) in 1:10 ratio exposed to microwave radiation 800 W, 2450 MHz	UV-Vis, FTIR, TEM, XRD, EDX	2.5 mg mL⁻¹	*Staphylococcus aureus*,* Bacillus subtilis*,* Escherichia coli*,* Pseudomonas aeruginosa; Fungi: Aspergillus flavus*,* Aspergillus nidulans*	Well diffusion; Mueller-Hinton agar	24–48 h	Vijayan et al., [86]
Acacia *(Acacia baileyana)*	10 g powder in 100 mL bidistilled water for 10 min at 80 °C, filtered; 10 mL AuCl₂, 10 mL aqueous Acacia extract, 80 mL distilled water for 1, 12, 18, 24, 48 h at room temperature	UV-Vis, TEM	30, 60, 100 mg mL⁻¹; ampicillin	*Streptococcus mutans*,* Escherichia coli*	Well diffusion; Mueller-Hinton agar	—	Al Shayeb et al., [142]
Indian tea *(Camellia sinensis)*	50 g leaves in 500 mL water, infused for 24 h at 10 °C; 900 µL tea with 100 µL HAuCl₄ (10 mmol L⁻¹) at room temperature for 24 h, then air dried	UV-Vis, TEM	Read carefully	*Staphylococcus aureus*,* Klebsiella pneumoniae*	Antimicrobial tests on dyed cotton fabrics; bacteria grown in nutrient broth	18 h	Onitsuka et al., [143]
Pepper *(Capsicum annuum L.)*	10 g in 250 mL water, filtered; 125 mL extract + 500 mL 5 mmol L⁻¹ HAuCl₄·3 H₂O at 25 °C until color change	UV-Vis, FTIR, SEM, XRD	25 µg mL⁻¹	*Escherichia coli*,* Staphylococcus aureus*,* Bacillus subtilis; Fungi: Candida albicans*	MIC; Mueller-Hinton broth	Overnight	Baran et al., [144]
Eastern Indian lemongrass *(Cymbopogon flexuosus)*	2 mL essential oil in 10 mL acetone; 7 mL oil added to 0.1 mol L⁻¹ HAuCl₄ at 40–60 °C for 2 h; 1.5 mL NaOH 0.5 mol L⁻¹ added to maintain pH	UV-Vis, FTIR, SEM, XRD	140 mg mL⁻¹	*Staphylococcus aureus*,* Escherichia coli; Fungi: Fusarium oxysporum*	Well diffusion; Mueller-Hinton agar	Overnight	Pathania et al., [145]
Vernonia Cinerea *(Cyanthillium cinereum)*	10 g crushed leaves in 100 mL bidistilled water; 5 mL aqueous extract to 45 mL HAuCl₄ (1 mmol L⁻¹) at room temp for 5 min	UV-Vis, EDX, SEM, XRD	1, 2 mg mL⁻¹; Ampicillin, Chloramphenicol, Fluconazole 0.1 0.04 mg mL⁻¹	*Bacillus subtilis*,* Chromobacterium violaceum*,* Escherichia coli*,* Pseudomonas aeruginosa*,* Staphylococcus aureus*,* Streptococcus pyogenes; Fungi: Aspergillus niger*,* Fusarium oxysporum*,* Rhizopus oryzae*,* Penicillium expansum*	Disc diffusion; Czapek-dox and potato dextrose agar	Overnight	Singh et al., [153]
*S*pearmint *(Mentha spicata)*	0.5 mL commercial essential oil + 19 mL HAuCl₄ (10 mmol mL⁻¹) at room temp for 24 h, then oven dried	Extract: GC-MS; NPs: UV-Vis, TEM, DLS, XRD, FTIR	100, 50, 25, 12.5 µg mL⁻¹	*Listeria monocytogenes*,* Salmonella Typhimurium*,* Staphylococcus aureus*,* Escherichia coli*,* Bacillus cereus*	Well diffusion; Mueller-Hinton agar	24 h	Moosavy et al., [146]
Gnidia *(Lasiosiphon eriocephalus)*	10 g leaf powder in 250 mL water; 100 mL extract + 900 mL HAuCl₄·3 H₂O (1 mmol L⁻¹) at 80 °C in dark until color change	UV-Vis, SEM, TEM, XRD, FTIR, DLS, Zeta potential	10, 20, 40, 60, 80, 100 µg mL⁻¹	*Staphylococcus aureus*,* Escherichia coli*,* Klebsiella pneumoniae*,* Pseudomonas aeruginosa*	MIC; Mueller-Hinton broth	24 h	Datkhile et al., [147]
Ground pine *(Ajuga bracteosa)*	5 g dry powder in 250 mL deionized water at 100 °C; 4 mL HAuCl₄ + 10 mL boiled plant extract and infusion separately; water bath 40 °C for 24 h	UV-Vis, SEM, FTIR, XRD, GC-MS	0.04 mg mL⁻¹	*Escherichia coli*,* Staphylococcus aureus*,* Pseudomonas aeruginosa*	Well diffusion; Mueller-Hinton agar	24 h	Raja et al., [148]
Boldo *(Coleus forskohlii)*	8 g leaves in 100 mL deionized water for 30 min; 0.4 mL root extract + 1 mL HAuCl₄	Extract: GC-MS; NPs: UV-Vis, HR-TEM, PSA, FTIR, XRD	250, 500, 750, 1000 µg mL⁻¹; tetracycline	*Proteus vulgaris*,* Micrococcus luteus*	Disc diffusion; Nutrient agar	24–48 h	Dhayalan et al., [55]
Wild mint *(Mentha longifolia)*	84.94 mg in 250 mL bidistilled water; 0.1 g methanolic extract dissolved in 100 mL HAuCl₄, diluted to 500 mL with water at 70 °C for 3 min	UV-Vis, FTIR, TEM, XRD	2 mg mL⁻¹ NPs; Streptomycin 2 mg mL⁻¹	*Klebsiella pneumoniae*,* Staphylococcus aureus*,* Bacillus subtilis*	Disc diffusion; Mueller-Hinton agar	24 h	Rauf et al., [149]
Lemon balm *(Melissa officinalis L.)*	5 g dried sample mixed with silica spheres in 34 mL ethanol; 10 mL extract added dropwise to 40 mL HAuCl₄ 1 mmol L⁻¹ under vigorous stirring at room temp until color change	Extract: GC-MS; NPs: UV-Vis, HR-TEM, PSA, FTIR, XRD	29.4 mg mL⁻¹	*Staphylococcus aureus*,* Bacillus cereus*,* Pseudomonas aeruginosa*	Disc diffusion; Mueller-Hinton agar; MIC; Mueller-Hinton broth	24 h	Fierascu et al., [150]
Eyebright *(Euphrasia officinalis)*	50 g leaf powder in 500 mL deionized water for 30 min; 5 mL + 25 mL sterile water + HAuCl₄ to 1 mmol L⁻¹ at 65 °C until color change	FE-TEM, SAED, EDS, XRD, Zeta potential, FTIR	1–10 µg mL⁻¹	*Pseudomonas aeruginosa*,* Escherichia coli*,* Staphylococcus aureus*,* Vibrio parahaemolyticus*	Disc diffusion; Mueller-Hinton agar	24 h	Singh et al., [53]
Tree bean *(Parkia roxburghii)*	1 g dried leaf powder + 100 mL 1 mmol L⁻¹ HAuCl₄ aqueous solution, stirred 12 h at room temp	UV-Vis, FTIR, XRD, TEM	10 mg mL⁻¹	*Staphylococcus aureus*,* Escherichia coli*	Disc diffusion; Mueller-Hinton agar	24 h	Paul et al., [151]
Plantain banana *(Musa balbisiana)*	20 g boiled at 75 °C in 200 mL of distilled water for 1.5 h and filtered after cooling. Then, a 1 mmol L⁻¹ aqueous gold solution was prepared by dissolving 39.38 mg. 1 mL of homogeneous HAuCl₄ solution (1 mmol L⁻¹) to 15 mL of heated extract, under stirring at 350 rpm, 25 °C, 45 min until color change.	UV-Vis, FTIR, XRD, TEM, FE-SEM, EDAX, DLS	6,7 mg mL⁻¹	*Escherichia coli*,* Staphylococcus aureus*	Well diffusion; Mueller-Hinton agar	24 h	Maji et al., [152]

*Thermogravimetric analysis (TGA), Gas chromatography-mass spectrometry (GC-MS/MS) and High performance liquid chromatography/ultraviolet-visible (HPLC/UV-VIS), Electron diffraction (SAED), X-ray diffraction (XRD), Dynamic light scattering (DLS), Energy dispersive spectroscopy (EDX), Energy dispersive spectroscopy (EDS), Fourier transform infrared spectroscopy (FTIR), Raman spectroscopy (Raman), Ultraviolet-visible spectroscopy (UV-Vis), Field emission splitting electron microscopy (FE-SEM), Transmission electron microscopy (TEM), High resolution transmission electron microscopy (HRTEM), Scanning electron microscopy (SEM), Zeta Potential (Zeta)

Balasubramani et al. [132] reported excellent efficacy of AuNPs synthesized from *Albizia amara* Roxb against *S. aureus*, achieving inhibition exceeding that of the antibiotic ciprofloxacin by 133%. Similarly, Asker et al. [112] found that AuNPs from geranium (*Pelargonium graveolens*) inhibited *Streptococcus mutans* at a rate surpassing the standard antibiotic chlorhexidine (102%). Singh et al. [153] observed 103% inhibition relative to ampicillin against *Streptococcus pyogenes* with AuNPs from *Vernonia cinerea* (syn. *Cyanthillium cinereum*), which also showed antifungal activity superior to fluconazole against *Aspergillus niger*,* Fusarium oxysporum*,* Rhizopus oryzae*, and *Penicillium expansum.*

Balasubramanian et al. [85] also demonstrated antimicrobial efficacy against *S. pyogenes* using AuNPs synthesized from *Cressa cretica* at 100 µg mL⁻¹. Geranium is rich in citronellol, a compound with antimicrobial and antioxidant properties; Guimarães et al. [154] reported that citronellol exhibited significant bactericidal effects against *S. aureus* strains. Certain compounds present in AuNPs derived from mallow-smelling (*Pelargonium graveolens*) likely exert their effects by interacting with the bacterial cell membrane.

In general, studies reporting successful inhibition of Gram-positive bacteria with AuNPs synthesized from leaf extracts demonstrated high inhibition percentages. For example, AuNPs from paradise tree (*Simarouba glauca*) inhibited *S. aureus* and *S. mutans* by 80% and *B. subtilis* by 50% [120]. Similarly, AuNPs from mast tree (*Polyalthia longifolia*) Mumtaz et al. [121] orchid tree (*Bauhinia purpurea*) [86], and chili pepper (*Capsicum annuum L*.) [144] inhibited *B. subtilis* by over 70%.

Pahal et al. [130] and Hariharan et al., [133] tested AuNPs from wheat (*Triticum aestivum*) (3.0 mg mL⁻¹) and *Azima tetracantha* Lam. LC, respectively, finding inhibition rates greater than 60% against *Micrococcus luteus*. Deivanathan et al. [155] and Baran et al. [144] reported similar inhibition rates against *S. aureus* using AuNPs from dancer plant (*Codariocalyx motorius*) and almond (*Amygdalus communis*), respectively; notably, the former also inhibited *Candida albicans* by 90%.

In another study, Layeghi-Ghalehsoukhteh et al. exposed *K. pneumoniae*,* Bacillus cereus*,* E. coli*, and *S. aureus* to AuNPs synthesized using an aqueous extract of dandelion (*Tragopogon dubius*). Only Gram-positive bacteria showed sensitivity, possibly due to mechanisms similar to those discussed by Rotimi et al. [62]. Conversely, *Citrobacter freundii* and *K. pneumoniae*, both Gram-negative bacteria, were resistant to the synthesized AuNPs [54, 156].

Gram-positive bacteria possess a thick peptidoglycan layer that can help maintain cellular integrity against bactericidal agents such as AuNPs [113]. However, since the peptidoglycan layer is external and therefore more exposed [157], the plant-derived nanomaterials may present reactive molecules including alkaloids, glycosides, saponins, tannins, quinones [111], coumarin, trans-cinnamic acid, and biochanin A [131]. These compounds facilitate strong binding of AuNPs to both peptidoglycans and lipopolysaccharides on the bacterial surface, leading to cell membrane disruption.

The study by Lestari et al. [157] is unique among leaf-based investigations for exploring antimicrobial properties against *Propionibacterium acnes*, a topical bacterium. Using AuNPs synthesized from Suren (*Toona sureni* (Blume) Merr.), they observed a low minimum inhibitory concentration (MIC) of 31.5 µg·mL⁻¹ against biofilms. Although the synthesized AuNPs did not demonstrate direct antimicrobial activity, these NPs showed effectiveness in inhibiting biofilm formation by *C. acnes* and disrupting mature biofilms [9].

Gao et al. [158] reported that AuNPs with cationic surface charges exhibit superior antimicrobial activity. Cationic liposomes stabilized with anionic gold nanoparticles, as in the study mentioned, fuse with bacterial membranes under acidic pH conditions, suggesting a promising topical delivery system for *C. acnes*. This highlights once again the critical role of nanoparticle surface charge in antimicrobial efficacy.

Paul et al. [151] synthesized AuNPs from tree bean (*Parkia roxburghii*) using a methodology distinct from others, where leaf powder was directly reacted with HAuCl₄. The resulting nanomaterial successfully inhibited *S. aureus* at a concentration of 10 g L⁻¹. Pathania et al. [145] synthesized AuNPs using acetone extracts of East Indian lemongrass (*Cymbopogon flexuosus*), as the raw material was the essential oil rather than leaf powder, and acetone was considered a better solvent for this purpose by the authors. The maximum inhibition zone observed was 36 mm against *S. aureus*. The tree bean AuNPs are easily absorbed by microbial cells due to their small size, ranging from 5 to 25 nm. On the other hand, lemongrass essential oil is rich in citral, a phytocatalytic and antioxidant compound known for its biocidal activity [145], and its apolar nature also facilitates AuNP absorption.

Six studies reported greater success inhibiting Gram-negative bacteria. AuNPs synthesized from gnidia (*Lasiosiphon eriocephalus*) [147], Madagascar bush (*Strobilanthes crispa*) [159], black nightshade (*Solanum nigrum*) [139], and parsley (*Petroselinum crispum*) [129], at concentrations of 100, 200, 50, and 25 µg·mL⁻¹ respectively, showed promising activity against *E. coli*. AuNPs from sweet violet (*Viola odorata*) were effective against *Salmonella typhimurium* (80.9% inhibition) and *E. coli* (89% inhibition) compared to the antibiotic streptomycin [113]. Shah et al. [40] tested AuNPs from bird plum (*Sageretia thea*) at 200 µg·mL⁻¹ and observed sensitivity in *K. pneumoniae* surpassing that of the standard antibiotic by 134%. Nagalingam et al. [107] synthesized AuNPs using leaf extract from the plant *Alternanthera bettzickiana* and tested them against Gram-positive strains (*B. subtilis*,* S. aureus*,* M. luteus*) and Gram-negative strains (*S. typhi*,* P. aeruginosa*,* Enterobacter aerogenes*). *M. luteus*,* P. aeruginosa*, and *E. aerogenes* showed greater sensitivity to ciprofloxacin, the standard antibiotic, while *S. aureus*,* S. typhi*, and *B. subtilis* were less sensitive, indicating that the synthesized AuNPs were more effective against Gram-negative bacteria.

**Fig. 5:**
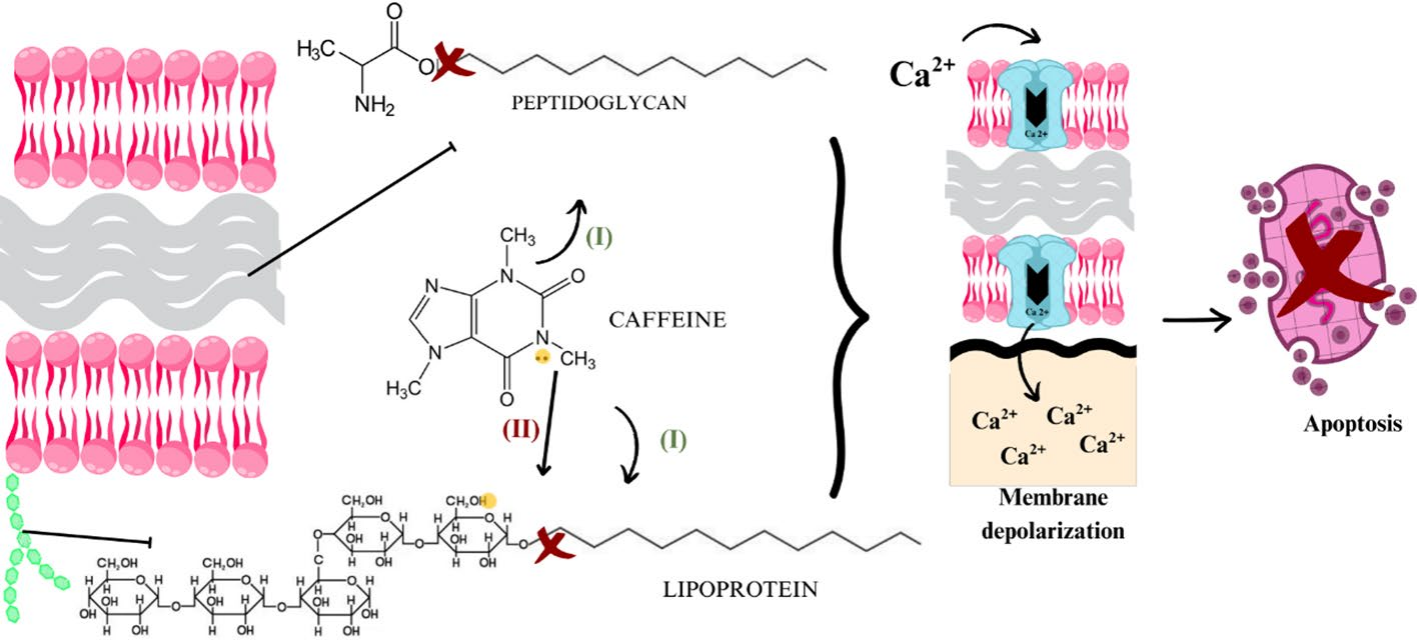
Reaction mechanism between caffeine and bacterial peptidoglycan (I) and lipoprotein (II) molecules causing membrane depolarization and consequent cell apoptosis, according to the retrieved studies on green plant-based metallic AuNPs and their antimicrobial effects

Several studies attributed these effects to the fact that Gram-negative bacteria possess a thinner cell wall compared to Gram-positive bacteria. Additionally, the synthesized AuNP leaf extracts contain flavonoids, terpenoids, caffeine (the most abundant in the vegetables mentioned) and protein compounds, as observed by Fourier-transform infrared (FTIR) analyses [54]. These compounds impart positive charges to the AuNPs, and the interaction between these positively charged compounds and the negatively charged lipopolysaccharides (LPS) of Gram-negative bacteria can decrease membrane permeability [15]. Consequently, AuNPs exhibit a sustained electrostatic attraction toward the negatively charged bilayer, facilitating their diffusion and inducing cell lysis by increasing cell wall permeability [85]. This process includes disruption of the membrane’s ion transport selectivity, leading to depolarization of the lipid membrane, increased influx of Ca²⁺ ions into the cytoplasm, and subsequent apoptosis [160] (Fig. 5, reactions I and II).

Eight studies reported promising results against both Gram-positive and Gram-negative bacteria. In one, AuNPs synthesized from Glory tree (*Clerodendrum trichotomum*) were effective in inhibiting *K. pneumoniae* and *S. aureus* [110]. AuNPs derived from Chinese bellflower (*Platycodon grandiflorus*) [118], tiger claw (*Martynia annua*) [111], Natal box (*Sarcococca saligna*) [137], and mountain violet (*Viola betonicifolia*) exhibited inhibition rates exceeding 60% against *E. coli* and *S. aureus*, over 50% against *Streptococcus spp*., and above 70% against *B. subtilis*. Notably, *Viola betonicifolia* AuNPs inhibited bacterial biofilms by more than 80%. AuNPs from Desert Rose (*Justicia glauca*) [1] showed increased sensitivity of *P. aeruginosa* at 50 µg mL⁻¹. Furthermore, Fanoro et al. [134] observed inhibition and bacterial death at the lowest tested concentration (62.5 µg mL⁻¹) with river willow (*Combretum erythrophyllum*) AuNPs, against *S. epidermidis*,* S. aureus*,* Mycobacterium smegmatis*,* Proteus mirabilis*,* E. coli*,* K. pneumoniae*, and *Klebsiella oxytoca*.

Regarding inhibition of both bacterial groups (gram positive and gram negative), some studies reported efficiencies surpassing standard antibiotics. Raja et al. [148] found that AuNPs from *Ajuga bracteosa* exhibited superior efficacy compared to ampicillin and gentamicin against *E. coli* (154%), *S. aureus* (123%), and *P. aeruginosa* (101%) at 0.04 mg mL⁻¹. Keskin et al. [122] demonstrated that AuNPs from gundelia (*Gundelia tournefortii*) achieved growth inhibition rates 8, 8, 4, 2, and 2 times greater than the antibiotics vancomycin (for Gram-positive bacteria) and colistin (for Gram-negative bacteria) against *S. aureus*,* B. subtilis*,* Candida albicans*,* E. coli*, and *P. aeruginosa*, respectively.

Finally, Do Dat et al. [138] was the only study to investigate the effect of AuNPs on *Salmonella enterica* using AuNPs from *Andrographis paniculata*, achieving efficiency for both *E. coli* and *S. aureus*. This indicates promising antimicrobial potential against both bacterial types.

The antibacterial inhibition mechanism of biosynthesized AuNPs may begin with the binding of polyphenols present in the extracts to bacterial proteins. This interaction alters membrane potential and subsequently reduces ATP synthase activity [134]. As a result, metabolic processes are diminished, leading to disruption of biological mechanisms by decreasing ribosomal subunit availability for tRNA binding [129]. Raja et al. [148] further explored the molecular basis of these effects, selecting two ligands with the highest affinity, Ergosterol and Decacetylajugrin IV, which exhibited strong hydrogen bond interactions with their target membrane proteins, the strongest among interaction types.

**Fig. 6:**
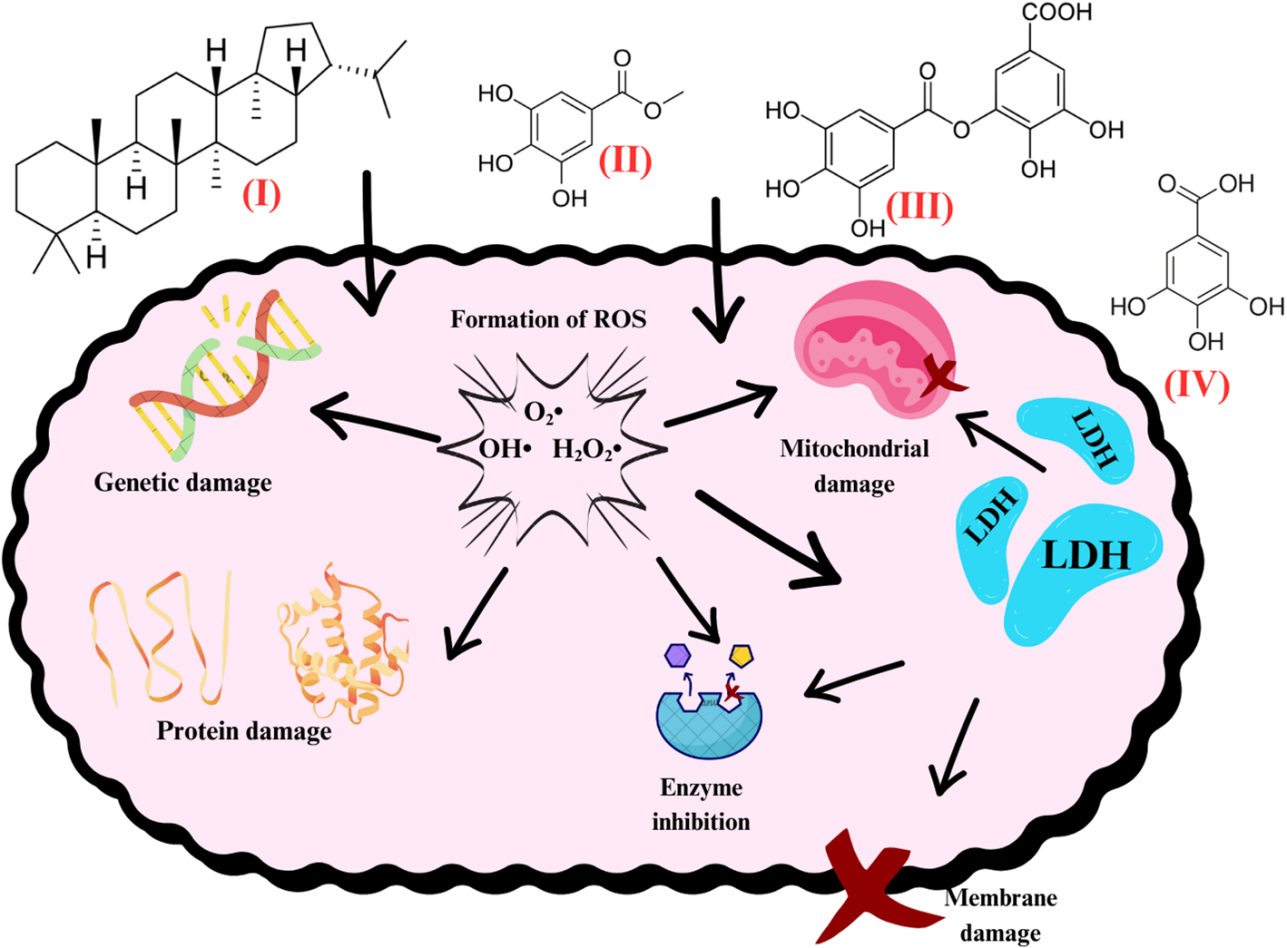
Illustrative diagram of triterpenoid (I), methyl gallate (II), digallic acid (III) and gallic acid (IV) known to generate ROS in bacteria which then lead to oxidative stress and deleterious bacterial effects through different mechanism of action, according to the retrieved studies on green plant-based metallic AuNPs and their antimicrobial effects

Many studies use extracts from commonly investigated species, allowing for interesting comparative analyses. Two studies, for example, synthesized AuNPs using aqueous extracts from king of bitters. Paramasivam et al. [128] reported efficacy against *S. aureus* (56%) and *Enterococcus faecalis* (70%) compared to standard antibiotics at a concentration of 100 µg mL⁻¹, while Chandran et al. [161] observed inhibition rates above 50% against *S. typhi*,* B. cereus*, and *P. aeruginosa* at 10 mg mL⁻¹. According to Chandran et al. [161] the inhibition was attributed to the production of reactive oxygen species (ROS), as the plant’s leaves contain terpenes and terpenoids. The ionic interaction between the AuNPs and bacterial cells can damage enzymes associated with the mitochondrial membrane that are active in the respiratory chain. This leads to an increase in hydroxyl radicals (OH·), which induce phospholipid peroxidation in membranes, ultimately causing cell death (Fig. 6).

Nanoparticles synthesized using boldo (*Peumus boldus*) extracts have also had their antimicrobial effects evaluated in two studies, with Naraginti et al. [162] using roots and Dhayalan et al. [55] using leaves. Naraginti et al. [162], at a nanoparticle concentration of 5 mg mL⁻¹, reported inhibition against *E. coli*,* P. aeruginosa*, and *S. aureus*. Meanwhile Dhayalan et al. [55] tested multiple concentrations (250 µg mL⁻¹, 500 µg mL⁻¹, 750 µg mL⁻¹, and 1000 µg mL⁻¹), finding dose-dependent inhibition ranging from 47% to 79% for *P. vulgaris* and 6% to 73% for *M. luteus*, compared to the standard. Although they used different parts of the plant and tested against different bacterial strains, both studies demonstrated that *Peumus boldus* AuNPs have potential as antimicrobial agents, regardless of the part of the plant used (similar to the case of ginseng AuNPs), opening avenues for future research on this species.

Regarding the mechanism of action, the authors agreed that inhibition zone diameters were larger for Gram-negative bacteria than for Gram-positive bacteria, attributing the latter’s resistance to the thick peptidoglycan layer. This layer consists of linear polysaccharide chains cross-linked by short peptides, rendering the cell wall rigid and hindering AuNP penetration [126].

In other assessments, Dorosti, et al. [123] and Chahardoli et al. [15] synthesized AuNPs using leaf extracts from sambacaetá (*Dracocephalum kotschyi*). Both studies tested the nanoparticles against *B. subtilis*,* S. aureus*, *E. coli*, and *P. aeruginosa*. Additionally, Dorosti, et al. [123] tested *B. cereus* and *P. vulgaris*, while Chahardoli et al. [15] tested *S. epidermidis* and *Serratia marcescens*. Despite using the same species and solvent for extraction, their results differed: Chahardoli et al.’s AuNPs were effective against all tested strains except *S. epidermidis* and *S. aureus* at 50 µg mL⁻¹, whereas Dorosti, et al. [123] AuNPs showed no antimicrobial efficacy. The proposed explanation for this discrepancy was the shorter synthesis time of two minutes in Dorosti, et al. [123] compared to the 15-minute synthesis used by Chahardoli et al. [15].

Many of the retrieved studies explored antimicrobial effects by synthesizing AuNPs from different species within the same genus, such as İpek et al. [163] and Shahriari et al. [124], who tested AuNPs synthesized using extracts from *Allium noeanum* Reut. and *Allium cepa* L., respectively. Even at low concentrations (8 mg mL⁻¹ and 16 µg mL⁻¹), the AuNPs from this genus showed activity against nine bacterial species: *B. subtilis*,* S. pneumoniae*,* S. aureus*, *Staphylococcus saprophyticus*,* S. typhimurium*,* P. aeruginosa*,* S. flexneri*, and *E. coli.* The genus Allium contains molecules such as allicin, diallyl disulfides, and ajoene, which are well-known for their efficacy against S. aureus, *E. coli*,* Salmonella* spp., and *Helicobacter pylori*, explaining the high antimicrobial efficiency observed with AuNPs in these experiments [163].

Allicin, a stable, natural compound found in garlic, is converted to ajoene when garlic cloves are crushed. This process also produces other unstable byproducts, such as 2-propenethial oxide (syn-propanethial-S-oxide) and thiacrolein, along with a conjugated thioketone intermediate [124]. The aqueous extract is prepared through maceration, resulting in the production of these compounds. They can then act as reducing agents in the synthesis of AuNPs and induce versatile antimicrobial effects due to their positively charged hydroxyl groups (as in 2-propenethial oxide) and negatively charged sulfur-derived groups.

Two mint species were used to synthesize AuNPs in the retrieved studies. The nanomaterial synthesized with spearmint (*Mentha spicata*) oil by Moosavy et al. [146] showed sensitivity against *E. coli* and *S. typhimurium* at the second-lowest tested concentration (25 µg mL⁻¹), while wild mint (*Mentha longifolia*) AuNPs [149] inhibited *S. aureus* by 50% at 2 mg mL⁻¹ relative to the standard antibiotic. The spearmint AuNPs were also tested against *S. aureus* but showed no inhibition, demonstrating that different species can contain distinct foliar molecules which may trigger additional antimicrobial effects, as was the case with wild mint. According to the authors, eighteen components were identified in the characterization, including carvone (78.76%) and limonene (11.50%), which may provoke reactions with Gram-positive bacteria similar to those reported by Rotimi et al. [62]

Two species belonging to the genus Nigella have also been explored for the synthesis of AuNPs and their antimicrobial applications: Black Cumin (*Nigella sativa*) Manju et al. [75] and Wild Fennel (*Nigella arvensis*) [109]. The antibacterial activity of Black Cumin AuNPs was greater against the Gram-positive *S. aureus* than against the Gram-negative *Vibrio harveyi* at a concentration of 10 µg mL⁻¹, as previously described in the seed extract chapter. The AuNPs of Wild Fennel were also effective against *S. aureus*, as well as *S. epidermidis*, *B. subtilis*,* E. coli*,* S. marcescens*, and *P. aeruginosa*. This demonstrates the opposite of the previous studies, demonstrating that species within the same genus can produce similar effects; in this case, both species are more efficient against Gram-positive bacteria than Gram-negative. The authors attribute this to the small size of the nanoparticles, which enhances their ability to penetrate the thick peptidoglycan layer of Gram-positive bacteria [75, 109].

Four species of jasmine have been used for AuNP synthesis and antimicrobial testing. For example, AuNPs derived from Wild Jasmine (*Clerodendrum inerme*) were the most efficient, inhibiting 99% of *B. subtilis*,* S. aureus*,* Klebsiella* spp., and *E. coli* at a concentration of 250 µg mL⁻¹, as well as inhibiting 90% of their biofilms [141]. AuNPs from Frangipani (*Plumeria alba*) inhibited *E. coli* at 400 µg mL⁻¹ [164], Jasmine (*Jasminum sambac*) inhibited *S. aureus* at 1 mg mL⁻¹ [125], and Caribbean Jasmine (*Plumeria pudica*) inhibited *B. subtilis* at 18.72 mmol L⁻¹ [116].

Khan et al. [141] demonstrated that the antimicrobial and antioxidant properties can be attributed to biologically active phytochemicals adsorbed onto the AuNPs, which are inherently bacteriostatic and fungicidal. These phytochemicals contain various molecular functional groups (-OH, -NO₂, -COOH, -SO₃H, -NH₂, -CONH₂, etc.) that play crucial roles in diverse biological activities against both Gram-positive and Gram-negative strains [164]. In the other studies, the concentration of compounds with these functional groups varied. For example, the AuNPs from Frangipani contain higher concentrations of compounds with -OH and -COOH groups, which have effects on Gram-negative bacteria as described in Fig. 4 [164]. Meanwhile, the AuNPs from Jasmine (*Jasminum sambac*) contain higher concentrations of molecules with -SO₃H, -NH₂, and -CONH₂ groups, which contribute to the mechanism described in Fig. 3. These groups can damage the very thick (~ 80 nm) peptidoglycan layer covalently linked with teichoic and teichuronic acids in Gram-positive bacteria [125].

Silva et al. [165], Sharma et al. [2]. and Onitsuka et al. [143] tested the antibacterial activity of AuNPs synthesized from leaf extracts of Punjab maple (*Acer pentapomicum*) [156] and two different Camellia species: *Camellia japonica* L [2]. and *Camellia sinensis* [143]. The assays were performed against *S. aureus*,* P. aeruginosa*,* K. pneumoniae*,* E. coli*,* Citrobacter freundii*, and *B. subtilis*.

All studies reported dose-dependent effects, with *K. pneumoniae* being the most sensitive to AuNPs from Punjab maple leaves, *B. subtilis* and *P. aeruginosa* most sensitive to AuNPs from *Camellia japonica* leaves, and *S. aureus* most sensitive to AuNPs from *Camellia sinensis*. This is another example showing that leaves from different species, yet belonging to the same genus, may contain common reactive molecules. For instance, Camellia species contain epigallocatechin as a major component, which can interact with Gram-positive bacteria due to its negative charge. The effects on *P. aeruginosa* are attributed to significant levels of vitamin E and phytosterols common to both Camellia species and *Acer pentapomicum*, which may share similar polarity with peptidoglycans, the main components of bacterial cell walls [166]. However, it is important to note that concentrations of these compounds may vary significantly between species, as they come from different regions with distinct climates: Punjab maple is native to Central Asia, while Camellia species originate from the eastern region near the Indian Ocean [104]. The reactive molecules involved in the AuNP synthesis process during Au reduction belong to the group of secondary metabolites and are responsible for plant defense and maintenance in environmental conditions [166]. Therefore, the predominance of different molecules in species according to their different regions becomes plausible.

Syntheses using ethanolic and methanolic leaf extracts have also demonstrated antimicrobial efficacy. For example, Ghramh et al. [26] applied AuNPs derived from an ethanolic extract of castor (*Ricinus communis*) leaves against *E. coli*,* Proteus mirabilis*,* Shigella flexneri*,* and S. aureus*, observing over 60% efficiency against all strains compared to controls treated with penicillin and streptomycin. Rahman et al. [80] used AuNPs from a methanolic extract of the same species against *E. coli*,* P. aeruginosa*,* K. pneumoniae*,* B. cereus*,* S. typhi*, and methicillin-resistant *S. aureus* (MRSA). Inhibition exceeded 90% for all strains, with *E. coli* inhibition 8% higher than the standard antibiotic ampicillin. Hutagalung et al. [167] and Fierascu et al. [150] also synthesized AuNPs with ethanolic extracts of sapodilla (*Manilkara zapota*) and lemon balm (*Melissa officinalis L*.), respectively. The former reported dose-dependent efficacy against E. coli and MRSA, whereas the latter found efficacy only against Gram-positive bacteria: common *S. aureus* and *B. cereus*.

Alcohol extractions can result in higher concentrations of antimicrobial compounds; alcohol is an amphiphilic molecule capable of extracting both hydrophilic and hydrophobic molecules. For example, Parmar and Sanyal [168] tested AuNPs synthesized with an aqueous extract of castor (*Ricinus communis*) and only achieved inhibitory success against *B. subtilis*. Amphiphilic molecules are capable of extracting palmitic acid, which is abundant in sapodilla (*Manilkara zapota*) leaves [150], 3-O-xylosides, -glycosides, and -rutinosides present in quercetin and kaempferol, as well as gallic and gentisic acids, in addition to phenylalanine, ricinine, and N-desmethyl and O-desmethyl analogues of ricin [26]. All these molecules have been described by the authors as anti-inflammatory. Moreover, AuNPs can carry higher concentrations of distinct reactive molecules on their surfaces, resulting in different antimicrobial effects, which may include damage to the peptidoglycan cell wall of Gram-negative bacteria, as well as membrane damage involving thiol groups in Gram-positive bacteria [167].

Folorunso et al. [54] synthesized AuNPs reduced using a leaf extract of soursop (*Annona muricata*) and tested them against Gram-positive bacteria *S. aureus*,* E. faecalis*, and *Clostridium sporogenes*, as well as the Gram-negative *K. pneumoniae*,* C. sporogenes* was the most sensitive to AuNP exposure, showing inhibition percentages above 50% compared to streptomycin. In contrast, *S. aureus* and *E. faecalis* were the least susceptible with approximately 40% inhibition zones, while *K. pneumoniae* was resistant. Both *S. aureus* and *E. faecalis* are facultative anaerobes, which may explain the reduced inhibitory effects of AuNPs, whereas *C. sporogenes* is an obligate anaerobe [137].

Some studies have also isolated specific compounds from plant leaves. For instance, El-Ghorab et al. [32] synthesized AuNPs using seven purified phenolic compounds (hesperetin, rosmarinic acid, caffeic acid, apigenin, quercetin, 7-methoxypyrrosmanol, and luteolin) extracted from oregano (*Origanum majoranum*) leaf powder and applied these against *P. vulgaris*,* S. typhimurium*,* E. coli*,* and S. aureus*. An inhibition rate of 63% was reported for the most concentrated AuNP solutions of each compound (AuNPs-OM1 through OM7). The authors noted that these AuNPs did not exhibit significant inhibitory properties against planktonic cells but were effective in controlling biofilm formation, with AuNP-OM6 (7-methoxyrosmanol) being the most efficient.

The increased cellular penetration and bacterial uptake after loading with 7-methoxyrosmanol is attributed to the apolar nature of this molecule, which facilitates the passage of functionalized AuNPs through bacterial cell membranes [82]. Once inside, interactions with mitochondria and bacterial DNA lead to negative bacterial effects and disruption of bacterial metabolism [169], resulting in biofilm production cessation [33].

**Fig. 7:**
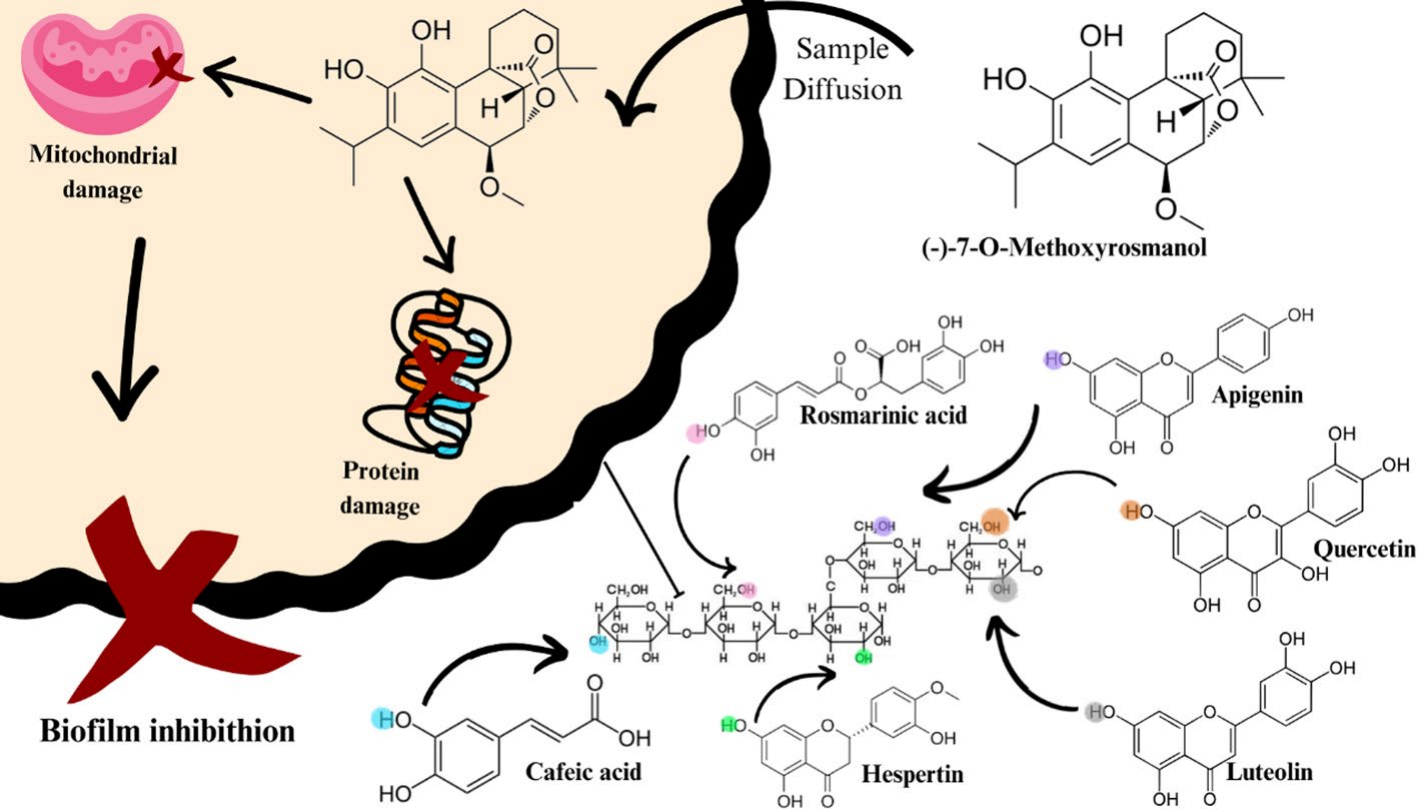
Reaction between 7-methoxypyrosmannol and bacteria mitochondrial membrane and proteins, resulting in biofilm inhibition, according to the retrieved studies on green plant-based metallic AuNPs and their antimicrobial effects. Possible reactions between biofilm and all molecules, each color represents each molecule, and each reaction leaves one water molecule

Biofilms are highly organized, structured, and coordinated communities of bacterial cells [170] that produce a thin, small barrier composed of sugars and proteins as a mechanism to increase stress tolerance and resistance to elimination [45]. Figure 7 illustrates the general mechanisms of biofilm inhibition caused by leaf-based plant AuNPs, similar to what occurs with root-based AuNPs illustrated in reaction II of Fig. 4. AuNPs reduced by other phenolic molecules also caused inhibitory effects, likely due to direct interaction with the bacterial biofilm itself. The five bacteria tested contain polysaccharides in the biofilm composition [171], and the interaction between the hydroxyl groups of these polysaccharides and those of hesperetin, rosmarinic acid, caffeic acid, apigenin, quercetin, 7-methoxypyrrosmanol and luteolin alters the polymeric structure, which in turn loses functionality and compromises the integrity of the biofilm [172].

The combination of diverse reactive molecules (polyphenols, flavonoids) present in plant extracts has contributed to the emergence of nanoparticle-based therapies as a powerful approach to combat bacterial resistance and improve antibiotic efficacy [173]. One of the main mechanisms underlying the inhibitory effect of metallic nanoparticles is their high affinity for sulfur and phosphorus atoms. The bacterial membrane is composed of numerous sulfur-containing proteins, and silver nanoparticles, for example, interact with these sulfur-rich proteins, affecting cell viability. Due to their similar polarity, nanoparticles penetrate bacterial envelopes via Van der Waals forces, receptor-ligand interactions, and hydrophobic interactions, damaging the structural integrity of the bacterial membrane [174]. Once inside bacterial cells, nanoparticles disrupt microbial pathways by affecting enzymatic proteins, DNA, ribosomes, and lysosomes, resulting in catastrophic effects for the bacteria [175]. Due to their ability to destroy DNA and disrupt transcription and translation mechanisms, nanoparticles are also believed to degrade genetic material responsible for antibiotic resistance expression in bacteria [83].

Gold nanoparticles synthesized by combining extracts from two different plant species have also been applied against bacteria. For example, those synthesized using aqueous leaf extracts of papaya (*Carica papaya*) and Madagascar periwinkle (*Catharanthus roseus*) [25]. The antimicrobial activity was tested against *S. aureus*,* E. coli*,* B. subtilis*, and *P. vulgaris*, showing greater effects against Gram-negative bacterial strains, approximately 75% higher. Different plant species contain different compounds that, when combined, may act synergistically to enhance antimicrobial activity. *Madagascar periwinkle* leaves contain α-tocopherol, ascorbic acid, and cystatin, which release H^+^ ions that can react with the negatively charged bacterial membranes, resulting in membrane rupture and cytoplasmic leakage [25]. Papaya leaves, on the other hand, contain papain, saponins, and chymopapain, proteolytic enzymes that may further contribute to accelerated bacterial degradation reactions [87] (Fig. 4, reaction I).

Rajendran [105], for example, tested AuNPs obtained from a leaf extract of earing princess (*Pergularia daemia*) against multidrug-resistant strains of *E. coli*,* P. aeruginosa*, and *B. subtilis*. Both Gram-positive and Gram-negative bacteria were inhibited, with the highest inhibition observed against *E. coli*. In another study, Diksha et al., [56] evaluated the antibacterial potential of AuNPs synthesized from a leaf extract of jamelão (*Syzygium cumini*) against resistant urinary tract pathogens (*E. coli*,* K. pneumoniae*,* P. vulgaris*,* Acinetobacter baumannii*,* S. aureus*, and *E. faecalis*). The synthesized AuNPs exhibited considerable antibacterial activity, with the second-lowest concentration tested (150 µg mL^− 1^) reducing bacterial growth by about 50% compared to the negative control.

Nanoparticles synthesized from the leaves of certain species showed no antimicrobial effects, such as AuNPs from eyeweed (*Euphrasia officinalis*) [53], Samar tree (*Haloxylon salicornicum*) [140], sugar apple (*Annona squamosa L.*) [115], the carnivorous plant *Nepenthes khasiana* [106], and Indian apple tree (*Ziziphus zizyphus*) [176]. However, the authors did not rule out the possibility that these synthesized AuNPs could be useful for other applications, especially considering their environmentally benign nature.

In other cases, low inhibition percentages were observed. For instance, AuNPs synthesized from Mozambique flower (*Elytraria acaulis*) [117] at 10 mg mL^− 1^ showed inhibition rates of only 20% and 25% against *E. coli and S. aureus*, respectively. Similarly, AuNPs from *Cotinus coggygria* Scop [135]. exhibited antimicrobial effects that were inferior to those of the pure plant extract.

### Gold nanoparticles derived from flowers extracts

Of the 13 studies found on AuNPs synthesized from flowers extracts, five reported greater efficiency against Gram-negative bacteria (Table 4). Gold nanoparticles from broccoli (*Brassica oleracea L*.) were effective against *S. typhi* [177]. at a concentration of 50 µg mL⁻¹. AuNPs from rosemary (*Rosmarinus officinalis*) and immortelle (*Helichrysum italicum*) [178] were more effective against *E. coli*, inhibiting 60% of its proliferation and 80% of its biofilm growth. Finally, AuNPs from tea tree (*Callistemon viminalis*) [179] and marigold (*Tagetes patula* L.) at respective concentrations of 1 mg mL⁻¹ and 300 µg mL⁻¹ were efficient against *P. aeruginosa*, with the marigold AuNPs also effective against *K. pneumoniae*. The marigold AuNPs also induced sensitivity in plant pathogenic bacteria, such as *Xanthomonas campestris*, *Ralstonia solanacearum*, and *Erwinia amylovora*.

**Table 4:** Studies on the synthesis of green gold nanoparticles from flower extract, including plant species, synthesis methods, characterization techniques, applied concentrations, tested bacterial strains, exposure time, and authors

Species	Synthesis Method	NP Characterization	NP concentrations	Description of Bacteria	Experimental Design and Culture Medium	Exposure Time	References
Jasmine Mango *(Plumeria alba)*	5 g of flower powder was separately mixed with 100 mL of deionized water and filtered; 10 mL of extract was separately added to 25 mL of HAuCl₄ (1 mmol L⁻¹) at room temperature, 2 min, centrifuged at 18,000 rpm and air⁻dried	UV-Vis, TEM, XRD, Zeta Potential, FT-IR, and EDAX	200, 300, and 400 µg mL⁻¹	*Escherichia coli*	Diffusion disk; Mueller-Hinton agar	24 h	Mata et al., [164]
Marigold *(Tagetes patula L.)*	Flowers were washed, crushed, and soaked in 100 mL of distilled water heated for 5 min; 5 mL of flower extract and 45 mL of HAuCl₄ 2 mmol L⁻¹ were mixed and stored in the dark for 24 h with constant agitation at 45 °C	UV-Vis, SEM, FTIR, and EDX	300 µg mL⁻¹	*Pseudomonas aeruginosa*,* Escherichia coli*,* Klebsiella pneumoniae*,* Staphylococcus aureus*,* Xanthomonas campestris*,* Ralstonia solanacearum*,* and Erwinia amylovora*	Well diffusion method; Mueller-Hinton agar	2 h	Hussain et al., [180]
Red Silk Cotton *(Bombax ceiba)*	2 g of dry mass in 80 mL of distilled water, vigorously stirred in a heater; 331 mL of 1 mmol L⁻¹ AuCl₃ aqueous solution was reduced using 1, 2, and 3 mL of extract for 30 min	UV-Vis and SEM	75.5, 151, and 226.6 µg mL⁻¹	*Salmonella typhi*,* Escherichia coli*,* Pseudomonas aeruginosa*,* Bacillus subtilis*,* and Staphylococcus aureus*	Diffusion disk; Mueller-Hinton agar	–	Aziz et al., 2025
Bottlebrush *(Callistemon viminalis)*	Different HAuCl₄ concentrations in aqueous extract: 1:1 to 1:8, constantly stirred at room temperature and then heated at 80 °C for 5–50 min	FE-SEM, UV-Vis, TEM, EDX, and FT-IR	1 mg mL⁻¹	*Escherichia coli*,* Staphylococcus aureus*,* Klebsiella pneumoniae*,* Pseudomonas aeruginosa*,* Candida albicans*,* C. krusei*,* Aspergillus sp.*,* and Trichoderma species*	Diffusion disk; Mueller-Hinton agar	24 h	Khan et al., [179]
Aconite *(Aconitum Laeve)*	1 mL of extract at 20 mg mL⁻¹ in 1 mL of HAuCl₄ (2 mmol L⁻¹)	UV-Vis, TEM, XRD, and FT-IR	10, 20, 40, and 80 µg mL⁻¹	*Escherichia coli and Staphylococcus aureus*	Diffusion disk; Luria-Bertani (LB) agar	24 h	Ahmad et al., [181]
Rosemary *(Rosmarinus officinalis)* and Immortelle *(Helichrysum italicum)*	25 g in 100 mL for 50 min at 80 °C; 4 mL of extracts in 1 mL of HAuCl₄·3 H₂O (1 mmol L⁻¹) for 10 min at room temperature; 18,000 rpm, washed with distilled water, air⁻dried	UV-Vis, DLS, Zeta Potential, FE-SEM-EDX, and FT-IR	1 g mL⁻¹	*Escherichia coli*,* Staphylococcus aureus*,* and Staphylococcus epidermidis*	Minimum Inhibitory Concentration (MIC); Mueller-Hinton broth	24 h	Ertas Onmaz et al., [178]
Mexican Poppy *(Argemona mexicana L)*	10 g of powder in 150 mL of 2⁻propanol; 3 mL of HAuCl₄·3 H₂O 1 mmol L⁻¹ in 3 mL of extract adjusted to 100 ppm at 80 °C for 30 min	UV-Vis	100 µg mL⁻¹	*P. aeruginosa resistant to imipenem and meropenem (IMP/MEM)*,* Klebsiella pneumoniae*,* and beta-lactamase producing Escherichia coli*	Diffusion disk; Mueller-Hinton agar	18 h	Téllez-de-Jesús et al., [186]
Carduus *(Carduus edelbergii Rech. f.)*	1 g of flower in 100 mL methanol, incubated 24 h at room temperature; 5 mL extract in 95 mL HAuCl₄·3 H₂O for 24 h at room temperature	UV-Vis, FT-IR, SEM, XRD, and EDX	10 mg mL⁻¹; standard antibiotic: amoxicillin	*Klebsiella aerogenes*,* Escherichia coli*,* Staphylococcus epidermidis*,* Staphylococcus aureus*,* and Acetobacter species*	Diffusion disk; Mueller-Hinton agar	24 h	Jamil et al., [182]
Broccoli *(Brassica oleracea L)*	1 g of fine powder in 200 mL of 90% ethanol at 37 °C for 12 h and filtered; different times (1, 2, 3, 6, 12, 24 h) and metal ion concentrations (10⁻² to 10⁻⁶ mmol L⁻¹) used in 1:1 volumes; 12,000 rpm for 20 min, dried at 45 °C	UV-Vis, FT-IR, SEM, XRD, and EDX	50 µg mL⁻¹	*Bacteria: Bacillus subtilis*,* Escherichia coli*,* Salmonella typhi*,* Pseudomonas aeruginosa; Fungi: Aspergillus sp.*,* Pneumocystis sp.*	Diffusion disk; Nutrient agar	24 h	Kuppusamy et al., [177]
African Helichrysum *(Helichrysum odoratissimum)*	1 mL of incense (18 mg mL⁻¹ stock) in 17 mL water at 45 °C for 15 min and filtered; 300 µL of 0.1 mol L⁻¹ HAuCl₄·3 H₂O added to 18 mL extract with 36 g gum arabic, reacted 15 min at room temperature	TEM, DLS, UV-Vis, TGA, XRD, and Zeta Potential	20–25 mg mL⁻¹	*Cutibacterium acnes*	Minimum Inhibitory Concentration (MIC); BHI broth	72 h	De Canha et al., [63]
Stinking Herb *(Paederia foetida Linn)*	20 g dried flowers crushed and boiled in 100 mL distilled water for 10 min; 10 mL extract added dropwise to 90 mL of 1 mmol L⁻¹ HAuCl₄ solution under constant stirring at room temperature for 8 h	UV-Vis, FTIR, XRD, and TEM	20 mg mL⁻¹	*Bacillus cereus*,* Escherichia coli*,* Staphylococcus aureus*,* and Aspergillus niger*	Diffusion disk; Nutrient agar	24 h	Bhuyan et al., [185]
Banana *(Musa acuminata colla)*	25 g dried flowers in Soxhlet using ethanol as organic solvent, then refluxed; 10 mL extract added to 90 mL HAuCl₄·3 H₂O (1.0 mmol L⁻¹) at room temperature until color change	UV-Vis, FTIR, XRD, TEM, and EDAX	125, 250, 500, 1000 µg mL⁻¹	*Staphylococcus aureus*,* Enterococcus faecalis*,* Escherichia coli*,* Salmonella typhi*,* Pseudomonas aeruginosa*,* Proteus mirabilis*,* and Klebsiella pneumoniae*	Diffusion disk; Nutrient agar	24 h	Valsalam et al. [218]

*Thermogravimetric analysis (TGA), Gas chromatography-mass spectrometry (GC-MS/MS) and High performance liquid chromatography/ultraviolet-visible (HPLC/UV-VIS), Electron diffraction (SAED), X-ray diffraction (XRD), Dynamic light scattering (DLS), Energy dispersive spectroscopy (EDX), Energy dispersive spectroscopy (EDS), Fourier transform infrared spectroscopy (FTIR), Raman spectroscopy (Raman), Ultraviolet-visible spectroscopy (UV-Vis), Field emission splitting electron microscopy (FE-SEM), Transmission electron microscopy (TEM), High resolution transmission electron microscopy (HRTEM), Scanning electron microscopy (SEM), Zeta Potential (Zeta)

Flowers can provide highly specific reactive compounds unique to each species, such as molecules from the amyrin and plumericin groups [164], eugenol, rosmarinic acid, and sulforaphane [177]. These molecules enhance the antibacterial potential due to their complex structures, which increase the surface area of the AuNPs available for interaction [164]. Possible mechanisms of AuNP interaction with the bacterial outer membrane include deposition on the cell wall, penetration through membrane porin channels, and disruption of membrane integrity [183]. After penetration through porin channels, AuNPs interact with DNA, inhibiting transcription, leading to reduced bacterial growth, cellular damage [33] and biofilm inhibition [9].

Biofilm inhibition can occur due to degradation of the exopolysaccharide (EPS) matrix, a molecular result of ecological stimuli [184] reduced bacterial adhesion, and repression of genes related to biofilm formation, according to [178] using AuNPs from flowers *Rosmarinus officinalis* and *Helichrysum italicum*. Biofilm development goes through four stages: initial adhesion, microcolony formation, maturation, and bacterial release [63]. Interfering with any of these stages increases the susceptibility of the pathogens present. In contrast, Aconite (*Aconitum laeve*) AuNPs demonstrated greater sensitization against Gram-positive bacteria [181]. At a concentration of 80 µg mL⁻¹, the nanomaterial inhibited *S. aureus* growth at the lowest tested concentration of 10 µg mL⁻¹. Fourier-transform infrared spectroscopy (FTIR) characterization revealed plant-derived molecules with amine and amide functional groups bound to the nanoparticle surface. These groups confer a negative charge to the nanomaterials, which are electrostatically attracted to the positively charged bacterial membrane of *S. aureus*, leading to membrane destabilization and rupture.

Jamil et al. [182] demonstrated promising results against both Gram-positive and Gram-negative bacteria, AuNPs synthesized with carduus (*Carduus edelbergii* Rech. f.) extract at a concentration of 10 mg mL⁻¹ showed inhibition rates of 80% for *K. pneumoniae*, 66.7% for *E. coli*, 56.25% for *S. epidermidis*, 85.7% for *S. aureus*, and 73% for *Acetobacter* species. This plant species contains phytochemical compounds derived from cholesterol, which can bind to the surface of the AuNPs, potentially enhancing their antimicrobial activity [53]. In addition to causing damage and altering membrane potential, leading to cytoplasmic leakage, AuNPs can bind to bacterial ribosomes or chromosomes, thereby inhibiting protein synthesis and inducing genotoxic effects [145].

Gold nanoparticles synthesized from skunk vine (*Paederia foetida* Linn) [185], Red Silk Cotton Tree (*Bombax ceiba*) [64] and *Argemone mexicana* L [186]. no antimicrobial activity. For the first two, the lack of activity was attributed to the relatively large size of the synthesized AuNPs, greater than 60 nm, which hinders their penetration into bacterial cells. In the third case, the NPs were tested against *P. aeruginosa* strains resistant to the antibiotics imipenem and meropenem, as well as *K. pneumoniae* and *E. coli* strains producing beta-lactamases. These antibiotic-resistant bacteria may possess more effective defense mechanisms against xenobiotic particles.

### Gold nanoparticles derived from fruit extracts

Promising antibacterial properties have been reported by 20 studies that used fruit extracts for the synthesis of AuNPs (Table 5). These extracts can be rich in proteolytic enzymes [187] as well as vitamins A and C, which are essential nutrients with anti-aging properties [29, 188] along with phenolic compounds and flavonoids [183]. The presence of proteolytic enzymes may explain the large number of studies involving fruit-derived AuNPs demonstrating efficacy against both Gram-positive and Gram-negative strains. For instance, Chokkalingam et al. [29] evaluated AuNPs synthesized from lychee (*Litchi chinensis*) fruit extract at 100 mg L⁻¹ against *E. coli* and *S. aureus*, reporting sensitivity in both species.

**Table 5:** Studies on the synthesis of green gold nanoparticles from fruit extract, including plant species, synthesis methods, characterization techniques, applied concentrations, tested bacterial strains, exposure time, and authors

Species	Synthesis Method	NP Characterization	NP concentrations	Description of Bacteria Used	Experimental Design and Culture Medium	Exposure Time	References
Pineapple *(Ananas Comosus (L.))*	20 g of fruit ground in 100 mL of water and centrifuged at 10,000 rpm for 15 min; jackfruit extract at 50% (v/v) added to a solution of AuCl₄ 2 mmol L⁻¹ in dark for 2 h at room temperature	UV-Vis, SEM, XRD, and FT-IR	10 µL (100 mg mL⁻¹)	*Strains: Escherichia coli and Streptobacillus sp.; Fungi: Aspergillus niger*,* Aspergillus flavus*	Disk diffusion; Muller Hinton Agar (MHA) (Hi-Media) and fungal species on Potato Dextrose Agar (PDA)	24 h (bacteria); 48 h (fungus)	Basavegowda et al., [195]
Jackfruit *(Artocarpus heterophyllus Lam.)*	20 g of fruit ground in 100 mL of water and centrifuged at 10,000 rpm for 15 min; jackfruit extract at 50% (v/v) added to a solution of AuCl₄ 2 mmol L⁻¹ in dark for 2 h at room temperature	UV-Vis, SEM, and FT-IR	1 mg mL⁻¹ of metanol	*Escherichia coli and Streptobacillus*	Disk diffusion; Muller Hinton Agar	24 h	Basavegowda et al., [187]
Oriental Melon *(Cucumis melo L)*	50 g in 250 mL of deionized water, boiled for 15 min; 10 mL extract and 100 mL HAuCl₄ at room temperature for 24 h	FE-SEM, TGA, UV-Vis, TEM, XRD, and FTIR	50 µL (16.7 mg mL⁻¹)	*Bacillus cereus*,* Listeria monocytogenes*,* Staphylococcus aureus*,* Escherichia coli*,* Salmonella typhimurium*	Disk diffusion; Mueller-Hinton Agar	48 h	Patra et al., [189]
Chinese Wolfberry *(Lycium chinense)*	10 g of fine powder obtained using a grinding machine and boiled for 1 h in 100 mL sterile water at 100 °C and filtered; 5 mL of filtered L. chinense stock mixed with 50 mL HAuCl₄ at 80 °C, 15,000 rpm for 20 min and air dried	UV-Vis, SEM, XRD, DLS, and FT-IR	100 mg L⁻¹	*Escherichia coli and Staphylococcus aureus*	Disk diffusion; Muller Hinton Agar	24 h	Chokkalingam et al., [29]
Chinese Hawthorn *(Crataegus pinnatifida)*	HAuCl₄ dissolved in 10% (v/v) fruit aqueous extract with a final concentration of 1 mmol L⁻¹ at 80 °C in oil bath; 16,000 rpm for 15 min and air dried	FE-SEM, UV-Vis, TEM, SAED, and DLS	1, 5, 10, 25, 50, 100 µg mL⁻¹ for MIC and 15, 30, 45 µg mL⁻¹ for Disk	*Escherichia coli and Staphylococcus aureus*	–	24 h	Kang et al., [183]
Pumpkin *(Cucurbita moschata)*	50 g of dried peel and 750 mL of distilled water (1:3 ratio); 250 mL of HAuCl₄·3 H₂O in 750 mL of plant extract (1:3 ratio); 15 min at 50 °C	EDX, UV-Vis, TEM, SEM, XRD, and FTIR	16.6 mg mL⁻¹	*Bacteria: Escherichia coli and Staphylococcus aureus; Fungus: Candida albicans*	Minimum inhibitory concentration; Mueller-Hinton broth	24 h	Kaval et al., [65]
Cranberry *(Vaccinium subg. Oxycoccus)*	10 g chopped fruits with 20 mL distilled water up to 100 mL; fruit extract (1 mL) combined with various amounts of HAuCl₄·4 H₂O (10 mmol L⁻¹) (1:0.25, 1:0.5, 1:1, 1:1.25, 1:2 mmol L⁻¹); temperatures: 20, 40, 60, 80, 100, 120 °C; time: 60–240 min	SEM, TEM, TGA, UV-Vis, XRD	25, 50, 75, and 100 µg mL⁻¹; standard antibiotic azithromycin 30 µg mL⁻¹	*Klebsiella pneumoniae*,* Pseudomonas aeruginosa*,* Bacillus subtilis*,* Enterococcus faecalis*	Disc diffusion; Mueller-Hinton agar	24 h	Queen et al., [66]
Bahera *(Terminalia bellerica)*	2 g fruit with 20 mL bidistilled water; 1:1 ratio of aqueous extract and HAuCl₄·3 H₂O (3 mmol L⁻¹) at 27 °C until color change	UV-Vis, FE-SEM, FT-IR, XRD	100 µg mL⁻¹	*Acinetobacter pneumoniae*,* Bacillus subtilis*,* Enterococcus faecalis*	Agar well diffusion; Mueller-Hinton	24 h	Chithambharan et al., [190]
Arjuna *(Terminalia arjuna)*	10 g fresh fruits in 100 mL bidistilled water, boiled at 50–60 °C for 5 min and filtered; 1 mL extract in 100 mL HAuCl₄ solution (1 mmol L⁻¹) at room temperature for 15 min	UV-Vis, TEM, XRD, FT-IR, Zeta Potential, DLS	500 µmol mL⁻¹ and 1000 µmol mL⁻¹ (100 µg mL⁻¹)	*Staphylococcus aureus*,* Klebsiella pneumoniae*,* Proteus vulgaris*	Disc diffusion; nutrient agar	24 h	Gopinath et al., [11]
Kiwi *(Actinidia deliciosa)*	100 g peeled and ground fruit in 100 mL water, filtered; 1 mL fruit extract added to 9 mL HAuCl₄·3 H₂O (1 mmol L⁻¹)	EDAX, EDX, XRD, EDAX, FT-IR, UV-Vis, TEM	100, 200, 300 µg mL⁻¹	*Pseudomonas aeruginosa*	Disc diffusion; Mueller-Hinton agar	24 h	Naraginti and Li, [162]
Elderberry *(Sambucus nigra L.)*	2.5% v/v in extract mixture; extract volume later doubled (5% v/v) and tripled (7.5% v/v); increasing volumes of HAuCl₄ solution (10 mmol L⁻¹) added again	UV-Vis, FTIR, DLS, TEM	—	*Staphylococcus aureus*,* Escherichia coli*	Minimum inhibitory concentration; Mueller-Hinton broth	24 h	Mariychuk et al., [191]
Privet *(Ligustrum vulgare)*	10 g fresh berries boiled with 90 mL distilled water; extract volume in HAuCl₄·3 H₂O to 1 mmol L⁻¹ at room temp until color change	UV-Vis, SEM, EDX, TEM, DLS, sp-ICP-MS, TGA, FT-IR, MALDI-TOF	16, 32, 50, 100, 150, 200 µg mL⁻¹	*Escherichia coli*,* Pseudomonas aeruginosa*	Disc diffusion; Mueller-Hinton agar	24 h	Singh and Mijakovic, [153]
Parijoto *(Medinilla speciosa)*	20 g fruit in 200 mL distilled water heated at 90 °C for 15 min; infusion with HAuCl₄·3 H₂O (1 mmol L⁻¹) at v/v ratios 1:4 (F₁), 1:6 (F₂), 1:8 (F₃)	UV-Vis, PSA, FTIR, TEM	Extract: 100 mg mL⁻¹; AuNPs: 400, 600, 800 mg mL⁻¹	*Pseudomonas aeruginosa*,* Staphylococcus aureus*	Agar well diffusion; Mueller-Hinton	18–24 h	Prihapsara et al., [192]
Ginseng-berry *(Panax ginseng)*	10 g powder in 150 mL sterile distilled water, autoclaved at 100 °C for 30 min; 100 mL extract to reach HAuCl₄ 1 mmol L⁻¹ at 23, 40, 60, 80, 90 °C	UV-Vis, FE-TEM, EDX, XRD, SAED, FT-IR	15, 30, 45 mg mL⁻¹	*Staphylococcus aureus*,* Escherichia coli*	Disc diffusion; Mueller-Hinton agar	24 h	Jiménez Pérez et al., [103]
Citrus fruit flavonoids Hesperidin (HDN) and Naringin (NRG)	4 mg mL⁻¹ and 8 mg mL⁻¹ respectively, stirred 24 h at room temp; 1 mL extract with 0.1 mL HAuCl₄ (5 mmol L⁻¹), magnetically stirred 4 h at 60 °C	UV-Vis, FT-IR, Zeta Potential, AFM	50 µg mL⁻¹; gentamicin 100 µg mL⁻¹	*Methicillin-resistant Staphylococcus aureus (MRSA)*,* Escherichia coli K1*	Disc diffusion; nutrient agar	24 h	Anwar et al., [169]
Mistletoe *(Viscum album)*	10 g fruit in 100 mL distilled water heated at 25 °C; infusion with HAuCl₄·3 H₂O (1 mmol L⁻¹)	UV-Vis, FE-TEM, EDX, XRD	50 and 100 µg L⁻¹	*Enterobacter*,* Salmonella typhi*,* Escherichia coli*,* Bacillus subtilis*	Minimum inhibitory concentration; Mueller-Hinton broth	24 h	Ishaq et al., [239]
Arak tree *(Salvadora persica)*	10 g dried fruit in 100 mL sterile water; 10 mL extract (10% w/v) in 40 mL HAuCl₄ (0.1 mmol L⁻¹), stirred in dark at room temp 24 h	Extract: GC-MS; NPs: UV-Vis, EDX, TEM, FTIR, Zeta Potential	100 µg mL⁻¹; standard antibiotic Gentamicin 4 µg mL⁻¹	*MRSA Staphylococcus aureus*,* Escherichia coli*,* Porphyromonas gingivalis*	Agar well diffusion; nutrient agar	24 h	Elhabal et al., [193]
Chinese Quince *(Chaenomeles sinensis)*	10 g fine powder of dried fruit in 100 mL distilled water; HAuCl₄·3 H₂O (1 mmol L⁻¹) added to diluted extract (70%) at room temp for 10 s	UV-Vis, FE-TEM, XRD	100 mg L⁻¹; neomycin	*Staphylococcus aureus*,* Escherichia coli*	Disc diffusion; Mueller-Hinton agar	24 h	Oh et al., [194]
Tamarind *(Tamarindus indica)*	5 g dry seeds in 100 mL double⁻deionized water, stirred up to 24 h at 80 °C; 5 mL HAuCl₄ (1 mmol L⁻¹) with 1 mL extract solution at 25 °C until color change	UV-Vis, FT-IR, AFM	1 mg mL⁻¹; standard antibiotic streptomycin 2 mg mL⁻¹	*Klebsiella pneumoniae*,* Bacillus subtilis*,* Staphylococcus epidermidis*	Disc diffusion; Mueller-Hinton agar	24 h	Ullah et al., [84]

*Thermogravimetric analysis (TGA), Gas chromatography-mass spectrometry (GC-MS/MS) and High performance liquid chromatography/ultraviolet-visible (HPLC/UV-VIS), Electron diffraction (SAED), X-ray diffraction (XRD), Dynamic light scattering (DLS), Energy dispersive spectroscopy (EDX), Energy dispersive spectroscopy (EDS), Fourier transform infrared spectroscopy (FTIR), Raman spectroscopy (Raman), Ultraviolet-visible spectroscopy (UV-Vis), Field emission splitting electron microscopy (FE-SEM), Transmission electron microscopy (TEM), High resolution transmission electron microscopy (HRTEM), Scanning electron microscopy (SEM), Zeta Potential (Zeta)

The effectiveness against Gram-positive strains such as *S. aureus*, observed in some studies, may be explained by the ability of AuNPs to disrupt the thick peptidoglycan layer. This has been demonstrated, for example, with AuNPs synthesized from pumpkin (*Cucurbita moschata*) at 16.6 mg mL⁻¹ [65] Parijoto (*Medinilla speciosa*) at 800 mg mL⁻¹ [192], Ginseng-berry (45 µg mL^− 1^) [103] Arak tree (*Salvadora persica*) (100 µg mL⁻¹) [193]. The latter also proved effective against *Porphyromonas gingivalis*, producing an inhibition zone 50% larger than that of the standard antibiotic. Regarding the detailed performance of the mentioned AuNPs against *S. aureus*, those synthesized from pumpkin (*Cucurbita moschata*) achieved a MIC₅₀ of 0.004 µg mL⁻¹, while the AuNPs from Parijoto (*Medinilla speciosa*) and the Arak tree (*Salvadora persica*) demonstrated inhibition rates exceeding 55%.

Fruit-derived AuNPs have also shown effectiveness in inhibiting the growth of Gram-negative bacteria such as *P. aeruginosa*. This includes AuNPs synthesized using kiwi (*Actinidia deliciosa*) [162], Parijoto [192] and Cramberry (*Vaccinium subg. Oxycoccus*) [66] at 300 µg mL⁻¹, 800 mg mL⁻¹, and 100 µg mL⁻¹, respectively. Among these, cranberry-derived AuNPs were the most effective against Gram-negative strains and also sensitized *K. pneumoniae*. This enhanced activity is attributed to cranberry’s richness in benzoic and hydroxycinnamic acids, as well as compounds such as proanthocyanidins (PACs), anthocyanins, catechins, and organic acids [66]. These molecules carry positive charges that interact with the negatively charged membranes of Gram-negative bacteria, triggering an inhibitory effect through membrane disruption [180].

Kaval et al. [65] reported that AuNPs derived from pumpkin induced the ROS generation in bacteria. Meanwhile, Prihapsara; Artanti; Ni’mah [192], noted that AuNPs from Parijoto contain flavonoids, saponins, and tannins. Flavonoids inhibit nucleic acid synthesis, disrupt cell membrane function, and impair energy metabolism [119]. Saponins alter the permeability of bacterial cell walls, bind to cell membranes, change cellular morphology, and cause protein lysis [111]. Tannins specifically target bacterial cell membranes, contributing to their antimicrobial effects [65].

Basavegowda et al. [187] synthesized AuNPs using pineapple (*Ananas comosus* L.) fruit pulp extract in combination with standard antibiotics ampicillin and penicillin to evaluate activity against *E. coli* and *Streptobacillus* spp. The antibiotics combined with pineapple-derived AuNPs showed inhibition zones 5 to 10% larger than those of the antibiotics alone. Similar results were reported by the same authors when testing AuNPs derived from jackfruit (*Artocarpus heterophyllus* Lam.) pulp against the same bacterial strains [195]. However, pineapple-derived AuNPs combined with penicillin were more effective against *E. coli*, while jackfruit-derived AuNPs paired with the same antibiotic showed greater efficacy against *Streptobacillus* spp. The authors hypothesize that bromelain, a proteolytic enzyme present in pineapple pulp, acted synergistically with penicillin. As an enzyme, bromelain may degrade biofilms, which *E. coli* uses as a defense mechanism [195]. On the other hand, jackfruit pulp is rich in morin-group compounds, anthocyanins, and steroidal molecules such as betulinic acid [187]. When combined with penicillin, these compounds may provide increased amounts of carboxyl and hydroxyl groups capable of releasing H⁺ ions, which can react strongly with the nitrogen-containing peptidoglycan components of the *Streptobacillus* spp. cell wall [33]. However, the lack of inhibition against *E. coli* may be due to these molecules not possessing the enzymatic potential observed in bromelain.

Four studies investigated the antimicrobial effects of AuNPs synthesized using extracts from different mulberry species. Kang et al. [183] Marija and Tasić et al. [114], Velmurugan et al. [136] and Abdalhamed et al. [119] biosynthesized nanoparticles mediated by aqueous extracts from Asian mulberry (*Crataegus pinnatifida*), Mediterranean blackberry (*Rubus* spp.), Chinese mulberry (*Cudrania tricuspidata*), and Egyptian mulberry (*Morus alba*), the latter combined with white wormwood (*Artemisia herba-alba*) extract. AuNPs from Chinese mulberry and Egyptian mulberry showed stronger inhibition against *E. coli*, a Gram-negative bacterium. Conversely, Mediterranean blackberry inhibited *Staphylococcus aureus* (Gram-positive) by 51%, while AuNPs from Chinese mulberry (*Cudrania tricuspidata*) showed no significant inhibition against the tested strains *B. subtilis* and *P. aeruginosa*.

**Fig. 8:**
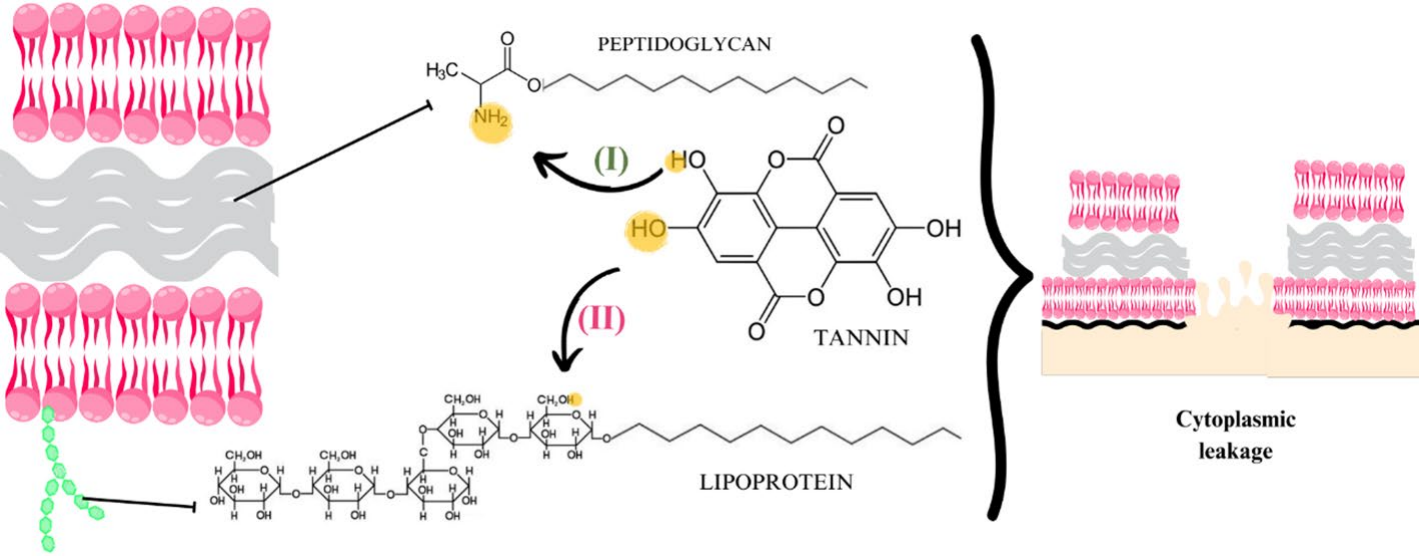
Potential reaction mechanisms between the tannin molecule, according to the retrieved studies on green plant-based metallic AuNPs and their antimicrobial effects: (I) with the hydroxyl group reacting with the H^+^ and the amino group (NH_2_^+^) of the peptidoglycan and (II) the hydroxyl group (OH) reacting with the glycoproteins, resulting in cytoplasmic leakage

Absorption bands attributed to protein amides and primary and secondary aromatic amines, such as tannins, terpenoids and saponins, were identified in the mulberry species reported in the previous paragraph [108]. Hydroxyl functional groups were also observed in alcohols and phenolic compounds [81]. Thus, the antimicrobial activity reported in these studies can be attributed to the functionalization of these compounds on the nanoparticle surfaces [196]. Similar to tannins, these compounds form hydrogen bonds between their hydroxyl groups and the peptidoglycan walls of bacterial strains, causing cellular damage through membrane rupture and cytoplasmic [67] (Fig. 8, reactions I and II).

Ullah et al. [84] synthesized AuNPs from Tamarind (*Tamarindus indica*) were tested against strains of *K. pneumoniae*,* B. subtilis*, and *S. epidermidis*. However, the inhibition percentages were below 40% compared to the standard antibiotic, which the authors suggest could be improved by increasing the concentration of AuNPs in the assays. Gold nanoparticles synthesized from Pseudocydonia (*Chaenomeles sinensis*) [194], privet (*Ligustrum vulgare*) [153], elderberry (*Sambucus nigra* L.) [191] and isolated flavonoids from citrus fruits (Hesperidin, HDN) and Naringenin, NRG) [169], showed no effectiveness against any of the tested strains. Flavonoids are widely recognized for their strong antimicrobial properties. Anwar et al., [169] tested these compounds against highly resistant strains such as MRSA *S. aureus* and neuropathogenic *E. coli* K1, which may explain their resistance even to these nanomaterials. The other studies attribute the lack of efficacy to the inert nature of gold; however, no studies have conclusively demonstrated this as the cause.

### Gold nanoparticles derived from stem extracts

A total of 11 studies assessed stem extracts in the synthesis of AuNPs, with mostly positive results against both gram-positive and gram-negative bacteria (Table 6). For example, AuNPs synthesized using Horsetail (*Equisetum diffusum*) were effective against *S. epidermidis* (145%), *L. monocytogenes* (154%), *S. aureus* (129%), *P. aeruginosa* (141%), *B. bronchiseptica* (137.5%), and *E. coli* (100%) at the highest tested concentration (40 µg mL⁻¹). The inhibition zones for the first three strains exceeded that of the control [197] AuNPs synthesized from Periploca (*Periploca hydaspidis*) inhibited *K. pneumoniae*, *E. coli*, and *X. campestris* by more than 60%, and also exhibited strong antifungal activity against *C. albicans* and *Penicillium chrysogenum* [84] Additionally, AuNPs from White Lozna (*Parthenium hysterophorus*) [198] and Trapoeraba (*Commelina nudiflora*) [199] were effective against *E. faecalis* at concentrations of 1 mg mL⁻¹ and 100 µg mL⁻¹, respectively.

**Table 6:** Studies on the synthesis of green gold nanoparticles from stem extract, including plant species, synthesis methods, characterization techniques, applied concentrations, tested bacterial strains, exposure time, and authors

Species	Synthesis Method	NP Characterization	NP concentrations	Description of Bacteria Used	Experimental Design and Culture Medium	Exposure Time	References
Dragon’s blood *(Croton draco)*	Aqueous stem extract (1:2) HAuCl₄ 1 mmol L⁻¹	UV-Vis, XRD, TEM, SEM, FTIR	1 mg mL⁻¹	*Staphylococcus aureus and Pseudomonas aeruginosa*	Agar well diffusion method on Mueller-Hinton	24 h	Elizalde-mata et al., [200]
Horsetail *(Equisetum diffusum)*	10 g of powdered material in 200 mL deionized water, stirred at room temperature for 5 h and filtered; 1 mmol of HAuCl₄ prepared by dissolving 33.97 mg in 100 mL DH₂O; extract: salt ratio (1:4) reacted for 10 min at 60 °C	UV-Vis, TEM, SEM, FTIR, and DLS	20, 30, and 40 µg mL⁻¹ and 30 µg mL⁻¹ ciprofloxacin; 80 for MIC µg mL⁻¹	*Staphylococcus epidermidis*,* Listeria monocytogenes*,* Staphylococcus aureus*,* Pseudomonas aeruginosa*,* Bordetella bronchiseptica*,* and Escherichia coli*	Agar well diffusion method on Mueller-Hinton; Minimum Inhibitory Concentration (MIC) in Mueller-Hinton broth	24 h	Assad et al., [197]
Azanza lampas *(Thespesia lampas)*	10 g of shade⁻dried stem powder extracted in 100 mL water; 10 mL of extract (5%, pH 6) with 90 mL HAuCl₄ 2 mmol L⁻¹ at 400 rpm for 24 h at room temperature	UV-Vis, XRD, SEM-EDS, HR-TEM, SAED, and FTIR	0.5 to 50 µg mL⁻¹	*Escherichia coli*,* Bacillus subtilis*,* Proteus vulgaris*,* and Salmonella typhi*	Agar well diffusion method on Mueller-Hinton	24 h	Nath et al., [201]
White parthenium *(Parthenium hysterophorus)*	10 g of powdered material with 100 mL bidistilled water; 10 mL extract with 90 mL salt at 70 °C for 60 min. Controlled: reaction time (15, 30, 45, 60, 100 min), metal ion concentration (0.5–5 mmol L⁻¹), pH (4, 6, 8, 10, 11), extract concentration (5–25%), reaction temperature (25–80 °C); optimized: 10% extract, pH 6, 1 mmol L, 40 °C, 60 min	UV-Vis, XRD, DLS, and Zeta Potential	1 mg mL⁻¹	*Enterococcus faecalis*,* Salmonella enterica*,* Escherichia coli*,* Staphylococcus aureus*	Agar well diffusion method on Mueller-Hinton	24 h	Leyu et al., [198]
Ababangai *(Oroxylum indicum)*	30 mg plant extract with 30 mL HAuCl₄ 1 mmol L⁻¹ in round⁻bottom flask in microwave oven (1,100 W) irradiated for 3 min at 800 W	UV-Vis, FTIR, X-ray, TG	0.25, 0.5, and 1 mg mL⁻¹	*Escherichia coli*,* Staphylococcus aureus*	Diffusion disk; Mueller-Hinton agar	-	Worakitjaroenphon et al., [202]
Trapoeraba *(Commelina nudiflora)*	10 g in 100 mL deionized water, heated at 60 °C for 15 min; 10 mL plant broth added with 190 mL aqueous HAuCl₄ (1 mmol L⁻¹), stirred at 37 °C, 150 rpm for 3 h	UV-Vis, FTIR, FE-SEM, XRD	100 µg mL⁻¹ and streptomycin 10 µg mL⁻¹	*Escherichia coli*,* Staphylococcus aureus*,* Salmonella typhi*,* Enterococcus faecalis*	-	-	Kuppusamy et al., [199]
Periploca *(Periploca hydaspidis)*	1:8 ratio of AuCl₃ 1 mmol L⁻¹ with boiled plant extract until color change	UV-Vis, FE-SEM, XRD, FTIR	125 µg mL⁻¹	*Escherichia coli*,* Klebsiella pneumoniae*,* Xanthomonas compestris*,* Candida albicans*,* Penicillium chrysogenum*	Diffusion disk; Mueller-Hinton agar	24 h	Shi et al., [237]
Eucalypto *(Eucalyptus sp.)*	2 g of powder in 100 mL of Millipore water under stirring until boiling for 10–15 min and filtered In. 30 mL of HAuCl₄ (1.0575 mmol L⁻¹) placed in 12 different flasks with **2** to 4 mL of extract, temperatures of 30, **40** and 50 °C.	UV-Vis, FE-SEM, XRD, FTIR, AFM	20, 40 e 60 µL (26, 52 e 78 mg mL⁻¹)	*Staphylococcus sp.*,* Pseudomonas sp.*,* Bacillus sp.*,* Escherichia coli*	Diffusion disk; Mueller-Hinton agar	24 h	Muthiah et al., [238]
Salicornia *(Salicornia brachiata)*	50 mg lyophilized aqueous extract in 50 mL Milli⁻Q water, filtered. 1 mL HAuCl₄ (1 mmol L⁻¹) added to 50 mL extract, stirred at 60 °C until color change	UV-Vis, FE-SEM, XRD, SEM, TEM	0.5 mg mL⁻¹	*Pseudomonas aeruginosa*,* Salmonella typhi*,* Escherichia coli*,* Staphylococcus aureus*	Diffusion disk; Mueller-Hinton agar	24 h	Ayaz Ahmed et al., [203]
Oal *(Amorphophallus paeoniifolius)*	0.1 g stem powder in 100 mL distilled water for 90 min to form extract. For AuNP synthesis, 100 µL 0.8 mmol L HAuCl₄ in 400 µL extract at room temperature until color change	UV-Vis, FE-SEM, XRD, SEM, FTIR, EDS	0.8 mg mL⁻¹	*Escherichia coli*,* Citrobacter freundii*,* Bacillus subtilis*,* Pseudomonas aeruginosa*,* Salmonella typhimurium*,* Staphylococcus aureus*	Diffusion disk; Mueller-Hinton agar	48 h	Nayem et al., [204]

* Thermogravimetric analysis (TGA), Gas chromatography-mass spectrometry (GC-MS/MS) and High performance liquid chromatography/ultraviolet-visible (HPLC/UV-VIS), Electron diffraction (SAED), X-ray diffraction (XRD), Dynamic light scattering (DLS), Energy dispersive spectroscopy (EDX), Energy dispersive spectroscopy (EDS), Fourier transform infrared spectroscopy (FTIR), Raman spectroscopy (Raman), Ultraviolet-visible spectroscopy (UV-Vis), Field emission splitting electron microscopy (FE-SEM), Transmission electron microscopy (TEM), High resolution transmission electron microscopy (HRTEM), Scanning electron microscopy (SEM), Zeta Potential (Zeta)

*Staphylococcus aureus* also showed sensitivity to AuNPs derived from White Lozna (*Parthenium hysterophorus*) [198] and traphoeba *Commelina nudiflora* [199] at respective concentrations of 1 mg mL⁻¹ and 100 µg mL⁻¹. Except for the Dragon’s Blood AuNPs [200], all studies tested antimicrobial activity against *E. coli*. Besides those mentioned earlier, AuNPs from White Lozna [198], Ababangai (*Oroxylum indicum*) [202], salicornia (*Salicornia brachiata*) [203],, and Trapoeraba (*Commelina nudiflora*) [199] were effective in inhibiting growth of *E. coli* at concentrations of 1 mg mL⁻¹, 10 µg mL⁻¹, 1 mg mL⁻¹, 1 mg mL⁻¹, 100 µg mL⁻¹, and 20 µL respectively, with inhibition percentages exceeding 50%. The study on *Salicornia brachiata* stands out for inhibiting only gram-negative bacteria, showing significant activity against *P. aeruginosa* (52%). The authors discuss this in relation to the thinner cell walls of gram-negative bacteria compared to gram-positive ones [203].

Regarding Gram-negative bacteria, metallic nanoparticles come into contact with the microbial cell membrane, and the positively charged metal ions interact with the negatively charged regions [198]. In the case of Gram-positive bacteria, the hypothesis is once again raised regarding the affinity of plant-derived molecules for the bacteria’s thick peptidoglycan cell wall [205]. They adhere firmly to the bacterial surface, resulting in significant cellular damage, complete destruction of flagella, accelerated biofilm formation, and biofilm aggregation [201].

According to the reviewed studies, all synthesized the AuNPs and their extracts at temperatures equal to or above 40 °C, suggesting that higher temperatures may enhance the availability of stem-derived molecules with stronger antimicrobial properties, as well as produce nanoparticles of smaller sizes (ranging from 3 to 30 nm). This hypothesis is supported by the fact that the AuNPs of Azanza lampas (*Thespesia lampas*) [201], from dragon’s blood (*Croton draco*) [200], pistache (*Pistacia integerrima*) [20] and oal (*Amorphophallus paeoniifolius*) [204] were extracted and synthesized at room temperature and did not demonstrate antimicrobial activity.

### Gold nanoparticles derived from aquatic plant extracts

Four studies investigated of plant-based AuNPs derived from aquatic plants (Table 7). This line of research is particularly important, given the abundance of pathogenic bacteria inhabiting marine environments that can contaminate economically important fish species [206].

**Table 7:** Studies on the synthesis of green gold nanoparticles from aquatic plants extract, including plant species, synthesis methods, characterization techniques, applied concentrations, tested bacterial strains, exposure time, and authors

Species	Synthesis Method	Methods Used for NP Characterization	Employed NP concentrations	Description of Bacteria Used	Experimental Design and Culture Medium	Exposure Time	References
Brown algae *(Sargassum plagiophyllum)*	10 g of dried powder with 100 mL of distilled water; 100–1000 µL of extract were added to 5 mL of HAuCl₄·xH₂O solution (1 mmol L⁻¹) at 60 °C until color change	FE-SEM, SEM, AFM, UV-Vis	10 µg mL⁻¹	*Escherichia coli and Salmonella typhi*	Agar well diffusion method; Mueller-Hinton agar	24 h	Dhas et al., [205]
Spiny algae *(Acanthophora spicifera)*	2.5 g of dried powder in 250 mL Milli⁻Q water at 60 °C for 20 min; 65 µL of HAuCl₄ 1 mol L⁻¹ solution gradually added to 250 mL aqueous extract under stirring at 60 °C for 4 h	UV-Vis, TEM, SEM, XRD, Zeta Potential, FTIR, and EDAX	25, 50, 75, and 100 µg mL⁻¹	*Vibrio harveyi and Staphylococcus aureus*	Agar well diffusion method; Mueller-Hinton agar	24 h	Babu et al., [207]
Sea grape *(Caulerpa racemosa)*	2 g of seeds boiled in 100 mL deionized water for 5 min; 1 mL extract added to 30 mL HAuCl₄ (2.5 × 10⁻⁴ mol L⁻¹) at boiling (373 K) for 2 min. Experiment repeated with 5, 10, 15, and 20 mL extract volumes to obtain colloids B₂, B₃, B₄, and B₅, respectively	UV-Vis, TEM, SEM, XRD, Zeta Potential, FTIR, and EDAX	25, 50, 75, and 100 µg mL⁻¹	*Aeromonas veronii and Streptococcus agalactiae*	Agar well diffusion method; Mueller-Hinton agar	24 h	Manikandakrishnan et al., [208]
Harvey *(Undaria pinnatifida)*	1 g mL⁻¹ extract with slow addition of 0.4 mmol L⁻¹ HAuCl₄ at room temperature for 24 h	UV-Vis, XRD, Zeta Potential, FTIR, and FESEM	0.54 and 11.81 µg mL⁻¹ for AuNPs; 5 and 60 µg mL⁻¹ for kanamycin and ampicillin	*Bacteria: Escherichia coli*,* Staphylococcus aureus*,* Pseudomonas aeruginosa; Fungi: Candida albicans and Candida auris*	Minimum Inhibitory Concentration (MIC); Mueller-Hinton broth	Overnight	González-ballesteros et al., [69]
Sargassum algae (*Sargassum incisifolium*)	2 mg of lyophilized extract dissolved in 10 mL distilled water and stirred for 10 min; 500 µL of HAuCl₄ (0.1 mol L⁻¹) added to 10 mL and reaction continued for 18 h at room temperature	UV-Vis, SEM, XRD, and Zeta Potential	0.4 mg mL⁻¹; 1 mg mL⁻¹ of vancomycin, ampicillin, and chloramphenicol	*Bacteria: Vancomycin-resistant Enterococcus faecalis*,* MRSA Staphylococcus aureus*,* Acinetobacter baumannii*,* and β-lactam-resistant Klebsiella pneumoniae; Fungus: Candida albicans*	Agar well diffusion method; Nutrient agar	24 h	Mmola et al., [206]

*Thermogravimetric analysis (TGA), Gas chromatography-mass spectrometry (GC-MS/MS) and High performance liquid chromatography/ultraviolet-visible (HPLC/UV-VIS), Electron diffraction (SAED), X-ray diffraction (XRD), Dynamic light scattering (DLS), Energy dispersive spectroscopy (EDX), Energy dispersive spectroscopy (EDS), Fourier transform infrared spectroscopy (FTIR), Raman spectroscopy (Raman), Ultraviolet-visible spectroscopy (UV-Vis), Field emission splitting electron microscopy (FE-SEM), Transmission electron microscopy (TEM), High resolution transmission electron microscopy (HRTEM), Scanning electron microscopy (SEM), Zeta Potential (Zeta)

Nanomaterials obtained from red algae (*Acanthophora spicifera*) [207], for example, was effective in inhibiting the strains *Vibrio harveyi* and *S. aureus* at 100 µg mL⁻¹, while AuNPs from harvey (*Undaria pinnatifida*) [69] at 11.81 µg mL^− 1^, demonstrated high effectiveness against biofilm-producing bacteria, such as *E. coli* and *P. aeruginosa*. Regarding marine bacteria, AuNPs from sea grapes (*Caulerpa racemosa*) effectively inhibited *Aeromonas veronii* and *Streptococcus agalactiae*, both commonly found in seafood and responsible for foodborne infections [208]. AuNPs derived from brown seaweed (*Sargassum plagiophyllum*) caused membrane damage in *S. typhi*, as observed by TEM [205]. The authors attributed the elimination of both Gram-positive and Gram-negative bacteria to the small size of the AuNPs, which ranged from 10 to 20 nm. These NPs more easily penetrate bacterial cells and entangle with organelles, causing damage.

Aquatic plants, however, remain underexplored, with only one study testing AuNPs against multidrug-resistant bacteria. Using an extract from sea holly (*S. incisifolium)* [206], the NPs showed no inhibitory effects against the Gram-positive strains *E. faecalis* resistant to vancomycin and *S. aureus* MRSA, nor against the Gram-negative multidrug-resistant *Acinetobacter baumannii* and β-lactamase-producing *K. pneumoniae*. Nonetheless, this represents a potential starting point for further research targeting such resistant bacterial strains.

### Gold nanoparticles derived from bark/peel extracts

A total assessed AuNPs derived from bark/peel extracts, with highly heterogeneous antimicrobial effects (Table 8), while showing promise in terms of reuse and sustainability. For example, AuNPs synthesized from Patala (*Stereospermum chelonoides*) affected only Gram-negative bacteria [209], inhibiting *E. coli* and *P. aeruginosa* at a concentration of 1 mg mL⁻¹. This plant species contains polyphenolic and alcoholic compounds with positive charges that exert the previously described mechanisms against the negatively charged bacterial cell surfaces, leading to antimicrobial effects.

**Table 8:** Studies on the synthesis of green gold nanoparticles from bark/peel extract, including plant species, synthesis methods, characterization techniques, applied concentrations, tested bacterial strains, exposure time, and authors

Species	Synthesis Method	NP Characterization	NP concentrations	Description of Bacteria Used	Experimental Design and Culture Medium	Exposure Time	References
Watermelon *(Citrullis lanatus var)*	100 g of each part (red and green) in 50 mL of Milli⁻Q^®^ water for 10 min, 45 s microwave heating (1100 W at 2450 Hz) and filtered; 1, 3, and 5 mL to 1 mL of AuCl₄⁻ (500 ppm) for 2 h at room temperature	UV-Vis, XRD, SEM, and EDX	50 µL (2 g mL⁻¹)	*Escherichia coli and Staphylococcus epidermidis*	Diffusion disk; Nutrient agar	48 h	Chumsa-ard et al., [52]
Pomegranate *(Punica granatum)*	Aqueous extract of peel (1:100); 5 min; 56 °C	UV-Vis, XRD, TEM, FT-IR, and Raman	1 mg of coconut fiber in 43, 128, and 256 µg mL⁻¹ of AuNPs	*Staphylococcus aureus*,* Enterococcus faecalis*,* Escherichia coli*,* and Pseudomonas aeruginosa*	Minimum Inhibitory Concentration (MIC); Nutrient broth	10 h under agitation at 120 rpm	Silva et al., [67]
Langsat *(Lansium domesticum)*	10 g of fruit peels in 100 mL bidistilled water at 80 °C for 12 h; 2 mL extract in 18 mL HAuCl₄ (0.5 mmol L⁻¹) at room temperature until color change	UV-Vis, XRD, SEM, DLS, and FT-IR	32 µg mL⁻¹ and 16 µg mL⁻¹	*Staphylococcus aureus and Escherichia coli*	Diffusion disk; Nutrient agar	24 h	Shankar et al., [176]
Macadamia *(Macadamia integrifolia)*	10 g in 100 mL deionized water for 2 h at 95 °C; aqueous peel extract (1.5, 10, 15, 20, 25 mL in 1 mL HAuCl₄ 1 mmol L⁻¹); 22 h	UV-Vis, XRD, SEM, and EDS	50 µL (1.0, 1.5, 2.0, 2,5 mg m L⁻¹)	*Escherichia coli and Staphylococcus epidermidis*	Diffusion disk; Mueller Hinton agar	24 h	Dang et al., [28]
Cannabis *(Cannabis sativa)*	150 g in 600 mL of 40% ethanol solution. Constant extract concentration of 10% and metal ion concentrations varying from 0.5 to 5.0 mmol L⁻¹ at room temperature until color change	UV-Vis, XRD, SEM, and FT-IR	10% v/v of Cannabis sativa extract with 2 mmol L⁻¹ tetrachloroauric acid solution (for AuNPs). 1.2, 2.4, 4.8, 7.2, 9.5, 11.9 mg L⁻¹	*Pseudomonas aeruginosa*	Luria broth (LB)	24 h	Michailidu et al., [210]
Cowpea *(Bauhinia variegata)*	0.1 g mL⁻¹ extract; 30 mL of 1 mmol L⁻¹ HAuCl₄ solution, 20 mL peel extract, stirred for 35 min on magnetic heating plate at 70 °C	UV-Vis, XRD, EDS, and FT-IR	50 µL (66 mg mL⁻¹)	*Bacillus subtilis and Escherichia coli*	Well diffusion method on Mueller-Hinton agar	24 h	Vaghela; Parmar; Mahyavanshi, [211]
Neem *(Nilavembu Choornam)*	7.5 g L⁻¹ filtered extract; 10 mL of extract with 40 mL HAuCl₄ solution (0.1 mmol L⁻¹) at 37 °C until color change; 10,000 × g for 10 min, air⁻dried	UV-Vis, XRD, EDS, SEM, and FT-IR	0.25; 0.5; 1 mg mL⁻¹	*Klebsiella pneumoniae*,* Staphylococcus aureus*,* Pseudomonas aeruginosa*,* and Enterococcus faecalis*	Well diffusion method on Mueller-Hinton agar	24 h	Almutleb et al., [212]
Yerba mansa *(Anemopsis californica)*	5 g biomass in 100 mL boiling water for 2 min; three extracts (water, methanol, isopropanol) in 2:5:3 volume ratio (extract : HAuCl₄ 1 mmol L⁻¹: deionized water) at room temperature until color change	UV-Vis and TEM	0.8; 1.6; 3.2; 6.4; 13; 26; 39 µg mL⁻¹	*Staphylococcus aureus and Escherichia coli*	Minimum Inhibitory Concentration (MIC); Nutrient broth	24 h	Martínez Avilés et al., [236]
Onion *(Allium cepa L.)*	100 g dried peel in 200 mL distilled water (1:2) at 85 °C for 10 min; 50 mL extract in 300 mL HAuCl₄·3 H₂O 75 mmol L⁻¹ (1:6) at room temp, 20 min (10,000 rpm), dried at 80 °C for 48 h	UV-Vis, TEM, XRD, FT-IR	16 µg mL⁻¹	*Bacteria: Staphylococcus aureus*,* Bacillus subtilis*,* Escherichia coli*,* Pseudomonas aeruginosa; Fungus: Candida albicans*	Minimum Inhibitory Concentration (MIC); Mueller-Hinton broth	24 h	İpek et al., [163]
Red sandalwood *(Pterocarpus santalinus L.)*	5 g peel in 100 mL water; 1 mL extract to 4 mL of different salt concentrations (0.3, 0.5, 1, 1.5, 2 mmol L⁻¹), temperatures (28, 35, 50, 75, 100 °C) under physiological conditions (pH 7.4)	UV-Vis, XRD, FTIR, and TEM	50 µg mL⁻¹; Chloramphenicol 1 mg mL⁻¹	*Staphylococcus aureus and Pseudomonas aeruginosa*	Diffusion disk; Mueller Hinton agar	24 h	Keshavamurthy; Srinath; Rai, [213]
Pomelo *(Citrus maxima)*	1 g peel in 100 mL deionized water, boiled 10 min. 100 µL chloroauric acid (100 mmol L⁻¹) in 10 mL extract, stirred at room temperature until color change	XRD, TEM, FT-IR	1.0, 1.5, 2.0 mmol L⁻¹ (1 g mL⁻¹)	*Staphylococcus aureus and Escherichia coli*	Minimum Inhibitory Concentration (MIC); Nutrient broth	24 h	Yuan et al., [214]
Curry *(Murraya koenigii)*	3 mL containing various doses of aqueous peel extract (0.4, 0.6, 0.8, 1 mL) and 1 mmol L⁻¹ HAuCl₄ in PBS (50 mmol L⁻¹, pH 7.4) at 40 °C for 24 h	FT-IR, TGA, UV-Vis, TEM, and zeta potential	5.6, 9.9, 3.4, 12.23 µg mL⁻¹	*Staphylococcus aureus*,* Pseudomonas aeruginosa*,* Klebsiella pneumoniae*,* and Escherichia coli*	Minimum Inhibitory Concentration (MIC); Nutrient broth	24 h	Mishra et al., [215]
Red Propolis *(Dalbergia ecastophyllum)*	HAuCl₄·3 H₂O (0.5 mmol L⁻¹) with extract solution (200 µg mL⁻¹) and its fractions (hexane, acetate, dichloromethane), then NaOH added to pH 7.0, stirred 1 h at room temperature	SAED, FTIR, TGA, UV-Vis, TEM, and EDX	200 µg mL⁻¹	*Staphylococcus aureus*,* Escherichia coli*,* Streptococcus mutans*,* and Candida albicans*	Minimum Inhibitory Concentration (MIC); Nutrient broth	24 h	Botteon et al., [216]
Patala *(Stereospermum chelonoides)*	5 g root peel in 100 mL bidistilled water at 60 °C for 20 min, filtered; 90 mL HAuCl₄·3 H₂O (1 mmol L⁻¹) in 10 mL extract in domestic microwave for 1 min	SAED, FTIR, TGA, UV-Vis, AFM	1 mg mL⁻¹	*Bacteria: Bacillus subtilis*,* Staphylococcus aureus*,* Escherichia coli*,* Pseudomonas aeruginosa; Fungi: Aspergillus nidulans*,* Aspergillus flavus*	Well diffusion method on Mueller-Hinton agar	considerable time	Francis; Koshy; Mathew, [209]
Banana *(Musa paradisiaca)*	1 g in 10 mL distilled water at 90 °C; 2 mL peel extract in 25 mL HAuCl₄ 1 mmol L⁻¹ at 353 K for 20 min	UV-Vis, XRD, FT-IR, TEM, EDX, and zeta potential	25, 50, 100 µg mL⁻¹	*Resistant Enterococcus faecalis*	Antibiofilm activity	considerable time	Manju et al., [75]

*Thermogravimetric analysis (TGA), Gas chromatography-mass spectrometry (GC-MS/MS) and High performance liquid chromatography/ultraviolet-visible (HPLC/UV-VIS), Electron diffraction (SAED), X-ray diffraction (XRD), Dynamic light scattering (DLS), Energy dispersive spectroscopy (EDX), Energy dispersive spectroscopy (EDS), Fourier transform infrared spectroscopy (FTIR), Raman spectroscopy (Raman), Ultraviolet-visible spectroscopy (UV-Vis), Field emission splitting electron microscopy (FE-SEM), Transmission electron microscopy (TEM), High resolution transmission electron microscopy (HRTEM), Scanning electron microscopy (SEM), Zeta Potential (Zeta)

Regarding *E. coli*, five studies reported antimicrobial efficacy against this species, including AuNPs synthesized from Pata-de-vaca [211], Natal Orange (*Citrus maxima*) [214, 217], Curry (*Murraya koenigii*) [215] and Red Propolis [216] in relation to *Pseudomonas aeruginosa*, some studies demonstrated inhibition rates above 70%, such as with AuNPs from Curry [215], Hemp (*Cannabis sativa*) [210] and Red Sandalwood (*Pterocarpus santalinus L.*) [213] at 12.23 µg mL^− 1^, 11.9 mg L^− 1^ and 50 µg mL^− 1^, respectively. Red Sandalwood AuNPs also showed high inhibition (> 85%) against *K. pneumoniae*. *S. aureus* showed sensitivity to AuNPs derived from Onion [163], de Red Sandalwood [213], curry [215] and Natal Orange [214] he latter demonstrating inhibition above 80%. Finally, *E. faecalis* was sensitive to AuNPs from neem (*Nilavembu Choornam*), with approximately 55% inhibition [212].

Manju et al. [75] investigated AuNPs synthesized from *Musa paradisíaca* (banana) against the multidrug-resistant pathogenic *E. faecalis*. The nanomaterial demonstrated a 66.7% inhibition of resistant bacterial biofilm at 100 µg mL⁻¹. Other studies have also explored bananas but using different species and plant parts. For example, Valsalam et al. [218] tested AuNPs from *Musa acuminata* leaves at 1000 µg mL⁻¹, Maji et al. [152] used AuNPs from earth banana (*Musa balbisiana)* fruit at 100 µg mL⁻¹, and Shankar et al. [176] synthesized AuNPs from banana (*Lansium domesticum)* peels at 32 µg mL⁻¹. These nanoparticles showed efficacy against *E. coli* and *S. aureus* [152, 176] *P. mirabilis* and *K. pneumoniae* [218] achieving inhibition rates exceeding 70%.

The AuNPs synthesized from aqueous banana peel extract (*Lansium domesticum*) contained various triterpenoids, such as lansiolic acid, 3β-hydroxynocera-8,14-dien-21-one, and 21α-hydroxynocera-8,14-dien-3-one, as well as triterpenoid glycosides and other organic compounds like lanic acid and methyl ester [176]. Triterpenoids are strong reducing and stabilizing agents that can induce excessive generation of ROS via metabolic oxygen pathways different from cellular respiration, including superoxide radicals (O_2_•⁻), hydroxyl radicals (OH•), and hydrogen peroxide (H_2_O_2_) [219]. The antibacterial effects of these ROS mainly involve the intracellular release of lactate dehydrogenase (LDH) through vacuole formation [176], causing damage to the cell membrane, DNA, and proteins, ultimately leading to bacterial cell death [85] (Fig. 3, reaction II). Additionally, the synthesized nanoparticles are small (please specify size), allowing better penetration into bacterial cells and triggering the mechanisms previously described in the leaves chapter.

An interesting alternative is the use of commonly discarded plant parts, such as macadamia shells, watermelon peels [28], oriental melon, and peach skins [189] as NPs sources. Dang et al. [28] for example, tested these AuNPs against *E. coli* and *S. epidermidis*. The AuNPs showed activity against both strains, with larger inhibition zones observed at interfering concentrations, indicating that AuNP effects are not always dose-dependent, as higher inhibition rates were not consistently linked to the highest AuNP concentration (125 µg µL⁻¹).

In another study, extracts from two different watermelon parts (*Citrullus lanatus* var.) were processed by infusion and filtration, then applied against *E. coli* and *S. epidermidis* [52]. Different extract proportions were tested, showing dose-dependent increases in inhibition zones with higher extract concentrations, although no specific mechanism of action was proposed [39] The variation in nanoparticle shapes, both spherical and triangular, observed in both studies may explain the differing results reported by the authors [87].

In another investigation, Baek and Patra [189] evaluated AuNPs synthesized from aqueous extracts of oriental melon (*Cucumis melo* L.) and peach skin (*Prunus persica*), combined with two standard proteins, rifampicin and kanamycin, testing their activity against *B. cereus*, *L. monocytogenes*, *S. aureus*, *E. coli*, and *S. typhimurium*. The AuNPs from oriental melon combined with kanamycin exhibited activity against all tested strains, with protein-AuNP-antibiotic combinations resulting in larger inhibition zones, particularly against *E. coli*, where inhibition nearly doubled compared to AuNPs alone. In contrast, both AuNP types combined with rifampicin showed effects only against *B. cereus*, *E. coli*, and *S. aureus*. The antibacterial effects of both standard antibiotics were enhanced when combined with the two plant-derived AuNPs.

This potentiation is attributed to the high vitamin C content in the fruit peels, as well as the presence of key metabolites such as coumaric acid and cyanidin-3-rutinoside, principal compounds in peach and oriental melon peels, respectively. Both these metabolites and the antibiotics possess multiple reactive hydroxyl and amino groups, which contribute to the bactericidal effects discussed in this article. When combined, these groups may amplify these antibacterial actions. The relatively lower antibacterial effect observed with rifampicin is likely due to fewer reactive groups in its molecular structure compared to kanamycin.

Silva et al. [67] investigated the antibacterial activity of cellulose nanofibers derived from coconut fiber (*Cocos nucifera*) and impregnated with AuNPs functionalized with pomegranate peel extract (*Punica granatum*) against gram-positive strains *S. aureus* and *E. faecalis*, as well as gram-negative strains *E. coli* and *P. aeruginosa*. The results demonstrated a reduction of over 60% in colony counts of *S. aureus*, *E. coli*,* and P. aeruginosa*. Additionally, free AuNPs synthesized from a mixture of the same plant extract combined with extracts from jaboticaba leaves and fruits also showed promising results in the study by Franzolin et al. [220] found an average inhibition of 98% against the strains *S. aureus*,* B. subtilis*,* E. faecalis*,* K. pneumoniae*,* S. Typhimurium*, and *P. aeruginosa*. Furthermore, the AuNPs were also effective against antibiotic-resistant bacteria, such as MRSA *S. aureus* and clinical isolates of *E. coli*. This effect appears to result from a higher concentration of molecular compounds on the surface of the nanomaterials, originating not only from two different plant species but also from different plant parts. Regarding pomegranate, the presence of tannins in the peel is likely responsible for the observed antibacterial activity, given that their chemical structure, like that of macromolecular polyphenols, contains numerous hydroxyl groups [170]. These functional groups can form strong hydrogen bonds with carbonylamide groups in peptide chains, such as peptidoglycans, within bacterial cell membranes [71].

### Gold nanoparticles derived from Sap extracts

Plant sap (also termed resin), founded in two studies, can also be used to synthesize AuNPs (Table 9), as it may contain several plant metabolites, both primary and secondary, that can act as reducing and capping agents [221]. In this sense, Marques et al. [68], Ferreira [222], and Milaneze et al. [223] synthesized AuNPs employing a bicuíba (*Virola oleifera*) resin extract and applied them to gram-negative *E. coli* and *P. aeruginosa* and gram-positive *S. aureus* and *E. faecalis*. All three studies reported positive results for *S. aureus*, with inhibitions of over 80%.

**Table 9:** Studies on the synthesis of green gold nanoparticles from Sap extract, including plant species, synthesis methods, characterization techniques, applied concentrations, tested bacterial strains, exposure time, and authors. Source: the author (2025)

Species	Synthesis Method	NP Characterization	NP concentrations	Description of Bacteria Used	Experimental Design and Culture Medium	Exposure Time	References
Bicuíba *(Virola oleifera)*	Aqueous extract of the sap 1 mg mL⁻¹, volumes: 1 mL, 2 mL, and 3 mL in 10 mL of acid	Factorial design with variables: extract solution volume, stirring, and time. UV-Vis, TEM, XRD, Zeta Potential, FT-IR, and Raman	1 mg mL⁻¹ of extract: 1 mL, 2 mL, and 3 mL in 10 mL of acid	*Staphylococcus aureus and Escherichia coli*	Minimum Inhibitory Concentration (MIC); Mueller Hinton broth	24 h, 48 h, and 72 h	Milaneze et al., [223]
Bicuíba *(Virola oleifera)*	1 mg of dried sap in 1 mL distilled water at room temperature until dissolved and then filtered; Aqueous sap extract and HAuCl₄ (2.5 × 10⁻⁴ mol L⁻¹) in a 3:7 ratio at 400 rpm, 6 min, 25 °C, pH 8.3	Factorial design with variables: extract solution volume, stirring, and time. UV-Vis, TEM, XRD, Zeta Potential, FT-IR, and Raman	0.019 mg mL⁻¹ or 9.9912 × 10⁻⁵ mmol mL⁻¹	*Staphylococcus aureus and Pseudomonas aeruginosa*	Minimum Inhibitory Concentration (MIC); Mueller Hinton broth	24 h, 48 h, and 72 h	Marques et al., [30]

*Thermogravimetric analysis (TGA), Gas chromatography-mass spectrometry (GC-MS/MS) and High performance liquid chromatography/ultraviolet-visible (HPLC/UV-VIS), Electron diffraction (SAED), X-ray diffraction (XRD), Dynamic light scattering (DLS), Energy dispersive spectroscopy (EDX), Energy dispersive spectroscopy (EDS), Fourier transform infrared spectroscopy (FTIR), Raman spectroscopy (Raman), Ultraviolet-visible spectroscopy (UV-Vis), Field emission splitting electron microscopy (FE-SEM), Transmission electron microscopy (TEM), High resolution transmission electron microscopy (HRTEM), Scanning electron microscopy (SEM), Zeta Potential (Zeta)

Ferreira [222] and Marques et al. [68] also reported significant inhibitions against *P. aeruginosa* of over 70%. The antimicrobial effects observed concerning AuNPs reduced with bicuíba (*V. oleifera*) extract are due to resin polyphenols, flavonoids, terpenes and tannins, comprising secondary metabolites [224].

Alcohols and phenols contain reactive hydroxyl groups that, in addition to promoting the reduction of gold ions, make AuNPs capable of interacting with the bacterial cell wall, altering its structure and, eventually, destroying it [90]. This group is also known to form chelate bonds with metals, trapping metals and inactivating certain cellular respiration enzymes (hexoknase, phosphofructokinase and pyruvate kinase), as noted for flavonoids [166]. All these mechanisms corroborate with the AuNPs synthesized employing the aforementions aqueous bicuíba sap extract, which caused cell wall damage in *S. aureus* and cytoplasmic leakage in *P. aeruginosa* [68].

### Other extracts

A total of 13 articles explored the synthesis of AuNPs using extracts from different plant species or various plant parts, reporting strong bactericidal effects (Table 10). Faiz et al. [225] observed inhibition rates up to 5% higher than standard antibiotics against strains such as *E. faecalis* when assessing AuNPs obtained from Eucalyptus and Jaborandi (*Eucalyptus globulus* and *Piper longum*), similar to Nagaraj et al. [226] with AuNPs from *Physalis minima* [225] in *S. aureus*,* P. aeruginosa*,* Streptococcus pneumoniae* and *E. coli* [226]. Khan et al. [227] synthesizing AuNPs using β-caryophyllene isolated from various plant species, achieved inhibition rates above 90%, as did Jha et al. [217] with AuNPs derived from fruits, leaves, and peel of pomelo (*Citrus maxima*) targeting resistant *P. aeruginosa*, and Botteon et al. [216] with AuNPs from jabuticaba and pomegranate as discussed in the peel section.

**Table 10:** Studies on the synthesis of green gold nanoparticles from various extracts, including plant species, synthesis methods, characterization techniques, applied concentrations, tested bacterial strains, exposure time, and authors

Species	Synthesis Method	NP Characterization	NP concentrations	Bacteria Described	Experimental Design and Culture Medium Used	Exposure Time	References
Marjoram (*Origanum majorana*)	1 kg of powder macerated with 80% MeOH at room temperature and then concentrated under reduced pressure using a rotary evaporator to a syrupy consistency. The concentrated methanolic extract yielded 60 g, and the dried extract was stored at 4 °C for in vitro and metabolomic studies.	UV-Vis, TEM, SEM, FT-IR	250 µg mL⁻¹	*Strains: Bacillus subtilis*,* Bacillus megaterium*,* Escherichia coli*,* and Proteus vulgaris; Fungi: Aspergillus niger*,* Fusarium solani*,* Candida albicans*,* and Aspergillus parasiticus*	Luria-Bertani broth (LB broth)	24 h	El-Ghorab et al., [32]
Olive (*Olea europaea*) and Egyptian Acacia (*Acacia nilotica*)	10 g of fruit in 100 mL distilled water; mixture of olive fruit extract and acacia bark extract in a 3:5 ratio; 5 mL of extract to 50 mL of 1 mmol mL⁻¹ HAuCl₄	UV-Vis, TEM, SEM, FT-IR	10 mg mL⁻¹	*Escherichia coli*,* Pseudomonas aeruginosa*,* and Klebsiella pneumoniae*	Diffusion disc; Mueller-Hinton Agar	24 h	Awad et al., [16]
Eucalyptus and Jaborandi (*Eucalyptus sp. and Piper Longum*)	10 g of bark from each plant in 1 L water. 1:16 (v/v) extract : HAuCl₄ 1 mol L⁻¹ at room temperature until color change	Not reported	0.625 mg mL⁻¹	*Bacteria: Staphylococcus aureus*,* Streptococcus mutans*,* E. faecalis; Fungus: Candida albicans*	Diffusion disc; Mueller-Hinton Agar	24 h	Faiz; Sivaswamy; Rohinikumar, [225]
Pomelo (*Citrus maxima*)	10 g of fruits, leaves, and dried peels crushed in 100 mL Milli⁻Q water, heated in an oil bath at 80 °C for 2 h in round⁻bottom flasks and filtered. 20 mL of extracts dropwise into 200 mL of 1 mmol L⁻¹ HAuCl₄ under constant stirring at room temperature until color change	UV-Vis, TEM, SEM, DLS	9.5 µg mL⁻¹, MIC Gentamicin (0.01 mg mL⁻¹)	*Resistant Pseudomonas aeruginosa*	Well diffusion method; Mueller-Hinton Agar; Minimum Inhibitory Concentration (MIC); Mueller Hinton Broth	24 h	Jha et al., [217]
Physalis (*Physalis minima*)	20 g of whole plant extract in 100 mL water; three different concentrations of 0.1 mmol L⁻¹ gold chloride solution and aqueous extract: 1:10, 1:5, and 1:3 at room temperature until color change	UV-Vis, TEM, SEM, FT-IR, XRD, EDX, and DLS	200 mg mL⁻¹	*Staphylococcus aureus*,* Pseudomonas aeruginosa*,* Streptococcus pneumoniae*,* and Escherichia coli*	Well diffusion method; Mueller-Hinton Agar; amoxicillin	24 h	Nagaraj et al., [226]
Rooibos (*Aspalathus linearis (Burm.f.)*)	1 mL (18 mg mL⁻¹) of extract prepared in ethanol added to 17 mL of salt and heated to 45 °C until color change	UV-Vis, XRD, FT-IR, TGA	2 mg mL⁻¹; standard antibiotic tetracycline 0.2 mg mL⁻¹	*Cutibacterium acnes*	Luria-Bertani broth (LB broth)	72 h	Staden et al., [228]
Flavonoids: chrysin, kaempferol, and quercetin	5 mL of aqueous solution of tetrachloroauric acid (0.025 mol L⁻¹) in 50 mL of 0.019 mol L⁻¹ aqueous solution of reduced L⁻glutathione (GSH), vigorously stirred for 30 min. Then conjugated with chrysin, kaempferol, and quercetin	UV-Vis, TEM, FT-IR, XRD, EDX, DLS	Final mixture concentrations: 960, 480, 240, 120, 60, 30, 15, 7.5, 3.25, 1.62, and 0.81 µg mL⁻¹	*Pseudomonas aeruginosa*,* Escherichia coli*,* Proteus vulgaris*,* and Klebsiella pneumoniae*	Luria-Bertani broth (LB broth)	24 h	Alhadrami et al., [229]
White poplar (*Populus alba*), Lantana (*Lantana camara*), and Hibiscus (*Hibiscus arboreus*)	Extracts prepared in 5, 10, and 15% (v/v), boiled and filtered. HAuCl₄0.3 H₂O (1 mmol L⁻¹) and plant extracts in 9:1 (v/v) ratio, constant stirring in the dark for 7 min at room temperature	UV-Vis, TEM, SEM, DLS, FT-IR	0, 10, 20, 30, 40, 50, 100 µg mL⁻¹	*Staphylococcus aureus and Escherichia coli*	Diffusion disc; Mueller-Hinton Agar	24 h	Acharya et al., [230]
African Geranium (*Galenia africana*) and African Potato (*Hypoxis hemerocallidea*)	50 mL boiled distilled water added to 5 g dried plant powder, centrifuged 2 h at 3750 rpm; 250 µL HAuCl₄0.2 H₂O 1.0 mmol L⁻¹ in 50 µL plant extract in 96⁻well plate (8.0 to 0.125 mg 300 µL⁻¹). Plate incubated 1 h at 70 °C, 40 rpm	UV-Vis, TEM, SEM, DLS, FTIR, HRTEM, TGA	8.0 to 0.125 mg/300 µL; ampicillin as standard	*Pseudomonas aeruginosa*,* Staphylococcus aureus*,* Staphylococcus epidermidis*,* and Escherichia coli*	Nutrient broth with Alamar Blue	24 h	Elbagory et al., [235]
Jabuticaba (*Plinia cauliflora*) and Pomegranate (*Punica granatum*)	2.2 g leaves and fruits boiled at 80 °C in 40 mL bidistilled water. Solutions exposed to 300 W Cermax Xenon lamp for 1 min for photoreduction and pH adjusted to neutral. Then 25 mmol L HAuCl₄ added until color change	UV-Vis, FTIR, SEM, Zeta Potential	55 mg mL⁻¹	*Bacteria: Staphylococcus aureus*,* Bacillus subtilis*,* MRSA*,* Enterococcus faecalis*,* Escherichia coli (clinical isolate)*,* Klebsiella pneumoniae*,* Salmonella Thiphymurium*,* Pseudomonas aeruginosa; Fungus: Candida albicans*	Minimum inhibitory concentration; Mueller-Hinton Broth	20 h	Franzolin et al., [220]
Isolated β-caryophyllene	200 mL deionized water, dissolving 1 mmol L⁻¹ HAuCl₄0.3 H₂O (pH 9.0), stirred at 60 °C. Dropwise solution of β-caryophyllene (1 mmol L⁻¹) added and stirred continuously at 60 °C	UV-Vis, FTIR, SEM, FETEM, XRD, EDX, Zeta Potential	128–2048 µg mL⁻¹(MIC); 64–256 µg mL⁻¹(antibiofilm); standard antibiotics and antifungals tetracycline and fluconazole	*Fungus: Candida albicans; Bacteria: Staphylococcus aureus*	Minimum inhibitory concentration; Mueller-Hinton Broth	24 h	Khan et al., [227]
Guduchi (*Tinospora cordifolia*)	100 µL HAuCl₄ solutions to 100 mg isolated plant extracts at room temperature until color change	SEM, EDX	1 g mL⁻¹	*Pseudomonas aeruginosa*	Well diffusion method; Mueller-Hinton Agar	24 h	Nath et al., [231]

*Thermogravimetric analysis (TGA), Gas chromatography-mass spectrometry (GC-MS/MS) and High performance liquid chromatography/ultraviolet-visible (HPLC/UV-VIS), Electron diffraction (SAED), X-ray diffraction (XRD), Dynamic light scattering (DLS), Energy dispersive spectroscopy (EDX), Energy dispersive spectroscopy (EDS), Fourier transform infrared spectroscopy (FTIR), Raman spectroscopy (Raman), Ultraviolet-visible spectroscopy (UV-Vis), Field emission splitting electron microscopy (FE-SEM), Transmission electron microscopy (TEM), High resolution transmission electron microscopy (HRTEM), Scanning electron microscopy (SEM), Zeta Potential (Zeta)

Faiz et al. [225] offers an interesting comparison with Babu et al. [207], who synthesized AuNPs from eucalyptus using only shoots. While Faiz et al. [225] achieved inhibition against all tested bacterial species, Babu et al. [207] inhibited only two out of four tested strains. This supports the notion that using multiple plant parts or multiple plant species provides a greater variety of antioxidant molecules on the AuNPs’ surface, enhancing their reactivity against bacteria. Additionally, none of the 13 studies employing the synthesis of mixed plant parts reported microbial resistance or low inhibition percentages.

Other examples include Alhadrami et al. [229] who synthesized AuNPs employing a mixture of chrysin, kaempferol, and quercetin of mixed parts of plants; Blom Van Staden et al. [228], who used whole Rooibos (*Aspalathus linearis*); and Guliani, Kumari and Acharya [230] synthesizing AuNPs from leaf extracts white poplar (*Populus alba*), Camará (*Lantana camara*) and Hibiscus (*Hibiscus arboreus*). All showed significant inhibition at the lowest tested concentrations against *S. aureus*,* E. coli* [230], *P. vulgaris*, *K. pneumoniae* [229], and *C. acnes* [228]. Lastly, Nath et al. [231] found that AuNPs from Guduchi inhibited *P. aeruginosa* with rates exceeding 60%. Benedec et al. [70] synthesized AuNPs from both oregano (*Origanum vulgare*) leaves and flowers and tested against *S. aureus*, thus using different parts of the same plant. Only the AuNPs synthesized with 1 mL of flower extract (250 µg mL^− 1^) exhibited antibacterial activity, while the extract alone showed no effect against the tested microbial strains. AuNPs synthesized with leaf extract, on the other hand, showed no activity against any tested bacteria. The authors suggest that different parts of the same plant can yield AuNPs with distinct characteristics and, consequently, varied effects. The compounds present in the oregano flower extract led to the synthesis of AuNPs more effective at inhibiting a broader range of bacteria compared to those synthesized from leaf extract, likely due to higher concentrations of antioxidant compounds, such as 13% eudesmol, 11.8% caryophyllene, and 10% acetate, along with trace amounts of terpinen-4-ol in the flowers. Leaves, in contrast, contain nearly 50% eucalyptol and 8% terpinen-4-ol [70].

Another mixture was proposed by Awad et al. [16], who synthesized AuNPs using a combination of olive fruit extract (*Olea europaea*) and gum arabic extract from acacia (*Acacia nilotica*), observing greater activity against gram-negative bacteria (*E. coli*,* K. pneumoniae*,* and Pseudomonas spp.*). This suggests that the molecules from the extracts adsorbed on the nanoparticle surfaces carry a positive charge [16]. Olives are rich in vitamin C, which may produce effects similar to those reported by Sellami et al. [127] with AuNPs from olive leaves against *K. pneumoniae*, by Al Shayeb et al. [142] with AuNPs from Acacia bark against *S. mutans* and *E. coli*, and by Vinay et al. [73] in gram-negative bacteria, as illustrated by the reactions presented in Fig. 3, reaction I and III.

### 12 antifungal and viral AuNP activities

It is important to note that some studies did not achieve success in eliminating bacterial pathogens. However, this does not rule out the possibility that the synthesized AuNPs could be useful for other applications, especially since they are environmentally friendly.

In this context, Islam et al. [20] used pistachio stem (*Pistacia integerrima*) combined with varying volumes of saline solution or gold salt solution (50–200 µL) and reported no antimicrobial activity against *K. pneumoniae*, *B. subtilis*, and *S. aureus* across all syntheses. However, antifungal activity was observed against three fungal species (*Alternaria solani*, *Aspergillus niger*, and *Aspergillus flavus*). Pistachio contains a high concentration of γ-tocopherol, which, although more reactive than α-tocopherol (commonly found in oilseeds), has lower antioxidant potential and consequently less interaction with bacterial system molecules. The reactivity mechanism of γ-tocopherol in some contexts involves neutralizing certain free radicals and reactive nitrogen species, such as peroxynitrite (ONOO⁻) and even amide groups, the latter present in chitin molecules. Since chitin is the primary component of fungal cell walls, this mechanism explains why pistachio-derived AuNPs showed greater efficacy against the fungi tested in the experiment.

Gopinath et al. and Dudhane et al. [11] tested AuNPs reduced with extracts of star fruit and leaves (*Terminalia arjuna*), aiming to improve the germination of onion (*Gloriosa superba*) seeds that were impaired by the manifestation of bacteria in their crops (*Proteus vulgaris* and *Klebsiella pneumoniae*), as well as Chithambharan et al. [190] tested antimicrobial potential of AuNPs obtained from Bahera (*Terminalia bellerica*). Neither of these trials demonstrated antimicrobial efficacy, suggesting that the genus Terminalia may not have sufficient reactive molecules to produce antimicrobial effects, regardless of the part of the employed plant part.

## Conclusion

The field of plant-based AuNP synthesis is relatively young, with most publications appearing after 2020. Consequently, many aspects remain unexplored. Most plant extracts used in the synthesis of AuNPs contain molecules from phenolic, flavonoid, and terpene groups. These molecules, rich in hydroxyl groups, exhibit a strong affinity for thiol groups in bacterial membranes, which likely explains the membrane damage and subsequent cytoplasmic leakage, comprising key bacterial cell death mechanisms reported across studies. *Escherichia coli* was consistently found to be more sensitive to the tested AuNPs than *S. aureus* and most other gram-negative bacteria. This difference is largely attributed to the peptidoglycan layer thickness: gram-negative bacteria have thinner walls, facilitating easier penetration of the nanoparticles and offering less resistance to degradation. Furthermore, many plant extracts contain polyphenolic compounds and carboxylic acid groups with alcohol and carboxyl moieties that interact with the negatively charged surfaces of gram-negative bacteria, enhancing the inhibitory effect.

Although gram-positive bacteria like *S. aureus* possess a thicker outer peptidoglycan layer, this layer is external and therefore more directly exposed to AuNPs and their surface molecules. Many extracts also contain compounds derived from quercetins and alkaloids, featuring amine and amide groups that exert deleterious effects on gram-positive bacteria through high reactivity and charge interactions. Studies that carefully controlled synthesis conditions, such as pH, reaction time, and agitation, produced more homogeneous NPs, resulting in more consistent antimicrobial efficacy and more reliable interpretation of results. This highlights the importance of optimizing synthesis parameters before applying AuNPs as antimicrobial agents. Investigations into synthesis methods employing amphiphilic solvents, such as alcohols and ketones, are warranted. These solvents tend to extract higher concentrations of reactive molecules, yielding nanoparticles with enhanced antimicrobial properties. A broad range of plant parts have been utilized for AuNP synthesis, leading to diverse NPs with varying antimicrobial effects. Additionally, NPs synthesized from the same species can exhibit different antimicrobial profiles depending on the concentration, the specific plant part employed, or when extracts from multiple species are combined. Roots, in particular, warrant further study due to their demonstrated effects against multidrug-resistant bacteria. However, research on AuNPs derived from root extracts remains scarce, indicating a clear need for expanded investigations in this area.

Several studies have reported promising antimicrobial activity against antibiotic-resistant and clinical bacterial strains, positioning plant-based AuNPs as an effective and sustainable alternative for combating resistant microorganisms. This emerging evidence opens significant avenues for future research and development. Finally, AuNPs exhibit great potential for drug development targeting both human health and environmental applications. This potential could usher in new strategies for addressing persistent and dangerous diseases. Furthermore, although some plant-based AuNPs lack direct antimicrobial effects, they may still hold promises for other biomedical applications, such as anticancer, antifungal, or antiprotozoal therapies.

## Supplementary Information

Below is the link to the electronic supplementary material.

Supplementary Material 1

## Data Availability

No datasets were generated or analysed during the current study.
