# Supplementary material for: A review on gold nanoparticles derived from plants and their antimicrobial applications
Source: Discov Nano. 2026 Feb 23;21(1):51. doi: 10.1186/s11671-026-04465-1 (PMC12929747; doi:10.1186/s11671-026-04465-1)
Supplement: Supplementary file 1 — Supplementary Material 1 [file 11671_2026_4465_MOESM1_ESM.docx]

Supplementary Material

Table 1. Studies on the synthesis of green gold nanoparticles from seed extract, including plant species, synthesis methods, characterization techniques, applied concentrations, bacterial strains tested, exposure time, and authors.

| **Species** | **Synthesis Method** | **Methods Used for NP Characterization** | **Employed NP concentrations** | **Description of Bacteria Used** | **Experimental Design and Culture Medium** | **Exposure Time** | **References** | **Country** |
| --- | --- | --- | --- | --- | --- | --- | --- | --- |
| Bottlebrush *(Callistemon citrinus)* | 30 g of crushed sample in 250 mL distilled water, stirred at 200 rpm for 24 h and filtered; 12.5 mL of plant extract added to 90 mL HAuCl₄ (1 mmol L⁻¹) solution, incubated for 6 h with continuous stirring | UV-Vis, TEM, XRD, SEM, FT-IR, and DLS | 20 mg mL⁻¹, 50 μg mL⁻¹ (antimalarial and antiprotozoal), 15.625–62.5 mg mL⁻¹ (antimicrobial) | *Strains: Escherichia coli (0157:H7:ATCC 35150), Vibrio alginolyticus (DSM 2171), Salmonella typhi (ACC), Staphylococcal enteritis (ACC), Staphylococcus aureus (ACC), Listeria ivanovii (X81135), Mycobacterium smegmatis (AJ131761); Protozoa: Plasmodium falciparum (NC_004318.3), Trypanosoma brucei brucei* | Minimum Inhibitory Concentration (MIC); Mueller-Hinton broth | 24 h | Rotimi et al., 2018 | South Africa |
| Mango *(Mangifera indica)* | 10 g seed powder in 100 mL distilled water for 5 h under constant stirring at room temperature, filtered; 60 mL extract added to 40 mL HAuCl₄ (1 mmol L⁻¹) at room temperature until color change | UV-Vis, TEM, XRD, Zeta Potential, FT-IR, Raman | 10–100 μg mL⁻¹ | *Staphylococcus aureus (ATCC) and Escherichia coli (ATCC)* | Minimum Inhibitory Concentration (MIC); Luria-Bertani (LB) broth | 4 h | Vimalraj et al., 2018 | India |
| Durian *(Durio zibethinus)* | 60 mg seeds in 90 mL HAuCl₄ (5 mmol L⁻¹) at room temperature for 1 h, refluxed with vigorous stirring at 97 °C for 5–6 h | UV-Vis, TEM, SEM, XRD, Zeta Potential, FTIR, EDAX | 1000 and 1500 mg mL⁻¹ | *Pseudomonas desmolyticum and Staphylococcus aureus* | Disc diffusion; Mueller-Hinton agar | 24 h | Vinay et al., 2019 | India |
| True cardamom *(Elettaria cardamomum)* | 2 g seeds boiled in 100 mL deionized water for 5 min and filtered; 1 mL extract added to 30 mL HAuCl₄ (2.5×10⁻⁴ mol L⁻¹) at boiling (373 K) for 2 min; repeated with 5, 10, 15, 20 mL extract to obtain colloids B₂–B₅ | UV-Vis, TEM, SEM, XRD, FTIR | 165, 330, 495, 660 μg mL⁻¹; antibiotic gentamicina | *Escherichia coli, Staphylococcus aureus, Pseudomonas aeruginosa* | Disc diffusion; Mueller-Hinton agar | 24 h | Rajan et al., 2017 | India |
| Wild rue *(Peganum harmala L)* | 5 g dried seeds in 250 mL distilled water at 80 °C for 90 min, filtered; 4 mL HAuCl₄ added to 100 mL extract (final 0.558 mmol L⁻¹ saline solution) at room temperature | UV-Vis, TEM, SEM, XRD, FTIR, EDX, FESEM | 100, 150, 200 μg mL⁻¹ | *Escherichia coli and Staphylococcus aureus* | Minimum inhibitory concentration; Luria-Bertani (LB) broth | 24 h | Moustafa et al., 2019 | Saudi Arabia |
| Barley *(Hordeum vulgare)* | 10 g grains with 90 mL distilled water at 100 °C for 20 min; HAuCl₄ 0.5–2.5 mmol L⁻¹; 60–90 °C, 1–30 min | TEM, SEM, EDS, AFM, DLS, FT-IR, MALDI-TOF, ICP-MS | 1–8 μg mL⁻¹ | *Escherichia coli and Pseudomonas aeruginosa* | Minimum Inhibitory Concentration (MIC); Mueller-Hinton broth | 24 h | Singh et al., 2024 | – |
| White acacia *(Moringa oleifera)* | Powder mixed with methanol 1:10 (m/v); aqueous solutions with 2.5, 5, 10 mg mL⁻¹ extract, 1:1 (v/v) with Au (III) chloride, stirred 24 h at room temperature, centrifuged at 2500 rpm for 20 min | UV-Vis, TEM, FTIR | 200, 400, 800, 1600 mg mL⁻¹ | *Escherichia coli and Staphylococcus aureus* | Minimum Inhibitory Concentration (MIC); Mueller-Hinton broth | 24 h | Figueroa et al., 2024 | Mexico |
| Mahua *(Madhuca longifolia)* | 5 g dried seeds powdered, boiled under reflux for 60 min, filtered; 0.4 mL extract per 1 mL HAuCl₄, pH 7.0 | UV-Vis, TEM, FTIR | 25, 50, 75, 100 μg mL⁻¹ | *Micrococcus luteus and Proteus vulgaris* | Minimum Inhibitory Concentration (MIC); Lysogeny broth (LB) | – | Dhayalan et al., 2024 | India |
| Black ebony *(Diospyros celebica)* | Extract: salt (2:1) at 60 °C for 2 h | UV-Vis, Zeta potential, TEM | 2.3 μg mL⁻¹ to 0.575 μg mL⁻¹ | *Bacillus subtilis, Staphylococcus aureus, Escherichia coli, Pseudomonas aeruginosa* | Minimum Inhibitory Concentration (MIC); Mueller-Hinton broth | 24 h | Ariani et al., 2025 | Indonesia |
| Quinoa *(Chenopodium quinoa W.)* | 4 g starch in 100 g total solution gelatinized at 82 °C for 30 min; five types of biofilms: control film, biofilms with 5 % and 2.5 % AuNPs, and two control biofilms (5 % and 2.5 % blank solution), incubated 35°C for 16 h | TEM, SEM, TGA | 0.1 and 0.2 mg mL⁻¹ | *Escherichia col (ATCC 25972)i and Staphylococcus aureus (ATCC 1901)* | 500 µL on 20 mm² biofilms; 100 µL aliquots transferred to saline, then 10 µL plated on TSA agar | 10 h | Pagno et al., 2015 | Brazil |
| Caraway *(Trachyspermum ammi)* | 1 g extract in 100 mL methanol and/or water; 5 mL aliquots added to 5, 10, 15, 20 mL HAuCl₄, stirred on heated shaker at 40–60 °C for 60 min | UV-Vis, FT-IR | 2.5, 3.33, 5, 10 μg mL⁻¹ | *Staphylococcus aureus, Klebsiella pneumoniae, Bacillus subtilis* | Disc diffusion; Mueller-Hinton agar | 24 h | Bawazeer et al., 2021 | Saudi Arabia |
| Mango *(Mangifera indica)* | 1 g dried powder extracted with 100 mL bidistilled water, boiled 5 min; 6 mL extract added to 40 mL HAuCl₄ 1 mmol L⁻¹ at 25 °C in dark for 24 h | UV-Vis, XRD, TEM, FT-IR, SAED, Zeta Potential | 10, 20, 30, 40, 50 mg mL⁻¹; DMSO | *Bacteria: Bacillus cereus, Escherichia coli, Staphylococcus aureus, Klebsiella pneumoniae, Salmonella typhimurium; Fungi: Cryptococcus neoformans, Candida albicans, Candida glabrata* | Agar well diffusion; Mueller-Hinton agar | 24 h | Donga et al., 2020 | India |
| Vidanga *(Embelia ribes)* | 1.25 g dried powder in 50 mL deionized water at 60 °C for 1 h; 1 mL extract added to HAuCl₄ (0.1 mmol L⁻¹) in 0.1–1 mL, final volume adjusted to 5 mL, reaction at room temperature until color change | UV-Vis, DLS, HR-TEM, FT-IR, XRD | 250, 500, 750, 1000 μg mL⁻¹ | *Escherichia coli and Staphylococcus aureus* | Agar well diffusion; Mueller-Hinton agar | 24–48 h | Dhayalan et al., 2017 | India |
| Mint flower *(Lallemantia royleana)* | HAuCl₄·3H₂O (1 mmol L⁻¹) prepared as solution A; 0.2 % (m/v) extract solution as B; 2–20 mL B added to 20 mL A under vigorous stirring at 25–80 °C, pH 3.5–12 for 24 h | UV-Vis, EDX, TEM, XRD, AFM, DLS | 180 μg mL⁻¹ (disc); roxithromycin 100 ppm, 10 µL; 180, 90, 45, 22.5, 11.25 μg mL⁻¹ MIC | *Agrobacterium tumefaciens, Bacillus subtilis, Escherichia coli, Staphylococcus aureus, Pseudomonas aeruginosa* | Agar well diffusion; Mueller-Hinton agar and MIC; Mueller-Hinton broth | 24 h | Iram et al., 2021 | Pakistan |
| Castor *(Ricinus communis)* | 100 mg seeds dissolved in 100 mL methanol; 100 mL extract reacted with 1 mmol L⁻¹ HAuCl₄ in 1:1–1:5 (extract:salt), 30–80 °C, 1–24 h | UV-Vis, FT-IR, DLS, TEM | 1 mg mL⁻¹; ampicillin | *Bacillus cereus, Salmonella typhi, MRSA, Escherichia coli, Klebsiella pneumoniae* | Agar well diffusion; Mueller-Hinton agar | 24 h | Rahman et al., 2021 | Pakistan |
| Caraway *(Trachyspermum ammi)* | 20 g seed powder mixed in 500 mL bidistilled water for 24 h; 6 mL extract added to 2 mL HAuCl₄ (10 mmol L⁻¹) on magnetic stirrer 30 min, then microwave 2 min (2.45 GHz, 300 W) | UV-Vis, XRD, TEM, DLS | 30 mg mL⁻¹ | *Listeria monocytogenes, Serratia marcescens* | Minimum Inhibitory Concentration (MIC); Lysogeny broth (LB) | 24 h | Perveen et al., 2021 | Saudi Arabia |
| Black cumin *(Nigella sativa)* | 200 g air⁻dried seeds in 200 mL water for 12 h; 2 mL extract added to 30 mL HAuCl₄ (1 mmol L⁻¹) at 100 °C, stirred 1 min, repeated with 5–8 mL | UV-Vis, XRD, TEM | 3, 5, 10 μg mL⁻¹ (disc); 2–10 μg mL⁻¹ (MIC); 20–80 μg mL⁻¹ (anti-biofilm) | *Staphylococcus aureus, Vibrio harveyi* | Disc diffusion; Mueller-Hinton agar | 24 h | Vaseeharan et al., 2016 | India |

*Thermogravimetric analysis (TGA), Gas chromatography-mass spectrometry (GC-MS/MS) and High performance liquid chromatography/ultraviolet-visible (HPLC/UV-VIS), Electron diffraction (SAED), X-ray diffraction (XRD), Dynamic light scattering (DLS), Energy dispersive spectroscopy (EDX), Energy dispersive spectroscopy (EDS), Fourier transform infrared spectroscopy (FTIR), Raman spectroscopy (Raman), Ultraviolet-visible spectroscopy (UV-Vis), Field emission splitting electron microscopy (FE-SEM), Transmission electron microscopy (TEM), High resolution transmission electron microscopy (HRTEM), Scanning electron microscopy (SEM), Zeta Potential (Zeta).

Table 2. Studies on the synthesis of green gold nanoparticles from root extract, including plant species, synthesis methods, characterization techniques, applied concentrations, tested bacterial strains, exposure time, and authors.

| **Species** | **Synthesis Method** | **Methods Used for NP Characterization** | **Employed NP concentrations** | **Bacteria Described** | **Experimental Design and Culture Medium Used** | **Exposure Time** | **References** | **Country** |
| --- | --- | --- | --- | --- | --- | --- | --- | --- |
| Sweet Flag *(Acorus calamus)* | For extraction at 60 °C and 100 °C, 30 g of washed rhizome was ground with 90 mL of water and heated for 15 min using a Soxhlet apparatus at 60 °C and 100 °C separately and filtered; 2.5 mL of appropriate extract, 2.5 mL of chloroauric acid 0.001 mmol L⁻¹ and 1 mL of buffer solution at appropriate pH (4, 7, and 9.2) were added. The mixture was stirred at 240 rpm using a magnetic stirrer until color change | UV-Vis, TEM, XRD, FT-IR | 1 g cotton with 750 mg mL⁻¹ AuNPs | *Staphylococcus aureus and Escherichia coli* | Cotton coating using pad-dry-cure method | 24 h and 48 h | Ganesan et al., 2015 | India |
| Apricot *(Mammea suriga)* | 50 g of root bark powder in 200 mL of water with continuous stirring for 5 h and filtered; 5 and 15 mL of aqueous root bark extract with 10 mL of HAuCl₄ for 24 h at 80 °C; centrifuged at 10,000 rpm for 20 min and dried at 80 °C in an oven | UV-Vis, SEM, EDX | 1 and 3 mmol L⁻¹ | *Bacillus subtilis, Staphylococcus aureus, Pseudomonas aeruginosa* | Disk diffusion; Mueller-Hinton Agar | 18 h | Poojary, 2016 | Italy |
| Ginger *(Zingiber officinale*) and Curcumin *(Turmeric rhizome)* | 20 g of ground ginger in 250 mL deionized water boiled for 20 min; 1 mL of 1 mmol HAuCl₄ diluted with deionized water to a final volume of 10 mL and boiled. Then, 1 mL of ginger extract was added to the boiling solution and stirred at 600 rpm until solution turned purple. 3.68 mg of curcumin rhizome (95 % purity) dissolved in 2 mL of 10 mmol L⁻¹ NaOH solution, volume adjusted to 10 mL with deionized water. 1 mL of 1 mmol L⁻¹ HAuCl₄ added to 8 mL of water. Then, 1 mL of freshly prepared curcumin solution added dropwise under stirring at 600 rpm for 20 min | UV-Vis, TEM, Zeta Potential, DLS, XRD, FT-IR | 80 mg mL⁻¹ (ginger); 1.84 mg mL⁻¹ (curcumin) | *Pseudomonas aeruginosa, Staphylococcus aureus, Escherichia coli* | Minimum Inhibitory Concentration (MIC); Mueller-Hinton Broth | 24 h | Kalantari et al., 2024 | Canada |
| Curcumin *(Turmeric rhizome)* | 10 g of turmeric immersed in 150 mL deionized water; 8 mL of 1 mmol L⁻¹ gold salt solution combined with 2 mL of turmeric extract for 5 min | FT-IR, Zeta Potential, DLS, XRD, TEM | 14 mg mL⁻¹ | *Staphylococcus aureus, Escherichia coli* | Nutrient agar | 18 h | Mohammad et al., 2025 | Iran |
| Neem *(Azadirachta indica)* and Ginger *(Zingiber officinale)* | 5 g of each compound in 100 mL water separately. 10 mL of neem and ginger extracts added to 100 mL of aqueous gold chloride 1 mmol L⁻¹ at room temperature until color change | EDS, SEM | 5 mg mL⁻¹ | *Bacteria: Streptococcus mutans, Staphylococcus aureus, E. faecalis; Fungus: Candida albicans* | Well diffusion; Mueller-Hinton Agar | 24 h | Kasabwala et al., 2021 | India |
| Stinking Benjamin *(Trillium govanianum)* | 20 g of dried and powdered rhizome in 50 mL distilled water; addition of 10 mg mL⁻¹ to 1 mmol L HAuCl₄ solution in three ratios: 1:1, 1:5, 1:10 (salt:extract) at 25 °C with constant stirring for 24 h | UV-Vis, FE-SEM, SEM, FT-IR, XRD | 40 mg mL⁻¹ (1:10) Azithromycin (50 μg, 6 μL–1) Gram-positive, Ciprofloxacin (30 μg, 6 μL–1) Gram-negative, Clotrimazole (50 μg, 6 μL–1) fungi. AuNPs 6–18 μL–1 | *Bacteria: Staphylococcus aureus, Pseudomonas aeruginosa, Escherichia coli, Bacillus subtilis, Klebsiella pneumoniae, Xanthomonas campestris; Fungi: C. albicans, Curvularia sp., R. oryzae, A. niger, A. alternaria,* *Paecilomyces sp.* | Disk diffusion; Mueller-Hinton Agar | nd | Zaman et al., 2022 | Pakistan |
| Licorice *(Glycyrrhiza uralensis)* | 10 g of root powder extracted for 1 h in 100 mL distilled water at 100 °C; HAuCl₄ (1 mmol L⁻¹) at 80 °C until color change | FE-TEM, XRD | 500, 1000, 1500 μg mL⁻¹ | *Escherichia coli, Staphylococcus aureus, Pseudomonas aeruginosa,* *Salmonella enterica* | Disk diffusion; Mueller-Hinton Agar | 24 h | Huo et al., 2018 | South Korea |
| Ginger *(Zingiber officinale)* | 45 mL Milli⁻Q water and 5 mL root extract; reaction parameters optimized for pH 4–10, reaction temperature 20–70 °C, time 0–210 min, metal ion concentration 0.25–4 mmol L⁻¹ | UV-Vis, FE-SEM, FT-IR, XRD | 0.1 mg mL⁻¹ each | *Staphylococcus spp.,* *Listeria spp., Bacillus spp.* | Disk diffusion; Mueller-Hinton Agar | 24 h | Velmurugan et al., 2014 | South Korea |
| Leadwort *(Plumbago zeylanica roots)* | 1:1 ratio, 24 h at 70 °C | UV-Vis, FTIR, XRD, TEM, DLS | 25 μg mL⁻¹ | *Escherichia coli, Staphylococcus aureus, Acinetobacter baumannii* | Disk diffusion; Mueller-Hinton Agar | 18 h | Salunke et al., 2014 | India |
| Black Gold *(Curculigo orchioides)* | 25 g of root boiled for 30 min in 100 mL sterile water. 1 mL of extract added to 5 mL HAuCl₄·3H₂O (1 mmol L⁻¹) at 80 °C for 2 h | UV–Vis, HR-TEM, XRD, EDX, FT-IR | 50 and 100 μg mL⁻¹ | *Escherichia coli, Klebsiella pneumoniae,* *Proteus vulgaris, Staphylococcus aureus, Serratia marcescens* | MIC; Mueller-Hinton Broth | 24 h | Thamilchelvan et al., 2023 | India |
| African Asparagus *(Asparagus racemosus)* | 5 g root powder in 50 mL distilled water; 1:1 (v/v) extract and HAuCl₄ (1 mmol L⁻¹) at 28 °C until deep wine red color, ensuring AuNP formation | FT-IR, SAED, SEM, TEM, FE-SEM, EDX, Zeta Potential, DLS | 20, 40, 80 μg mL⁻¹ (Streptomycin 25 μg and Gentamicin 25 μg) | *Escherichia coli, Bacillus subtilis, Klebsiella pneumoniae (urine), Pseudomonas aeruginosa, Staphylococcus aureus* | Disk diffusion; Luria-Bertani (LB) Agar | 24 h | Amina et al., 2020 | Saudi Arabia |
| Ginger *(Zingiber officinale)* | 50 g extracted with ethyl acetate in microwave at 630 W for 15 min obtaining 8.7 g extract; 5 mL extract added individually to reaction vessels containing 50 mL HAuCl₄ (1 mmol L⁻¹) at room temperature until color change | Extract: GC–MS; NPs: UV-Vis, TEM, FT-IR, Zeta Potential, AAS, DLS | 17.95, 8.97, 4.48, 2.24, 1.12, 0.56, 0.28, 0.14 μg mL⁻¹ (Streptomycin 30 μg mL⁻¹) | *Staphylococcus aureus, Escherichia coli* | Disk diffusion; Mueller-Hinton Agar | 24 h | Yadi et al., 2022 | Iran |
| Burdock *(Arctium lappa)* | 2 g powder with 80 mL ethanol at 40 °C for 24 h; 1 mL extract added to boiling 50 mL HAuCl₄·3H₂O (1 mmol L⁻¹) for 5 min | UV-Vis, XRD, TEM, XRD, TGA | 0.1, 0.2, 0.3, 0.5, 1, 2, 3, 4 μg mL⁻¹; Standard antibiotic ampicillin (0.01 mg mL⁻¹) | *Escherichia coli,* *Agrobacterium tumefaciens, Lactobacillus acidophilus, Staphylococcus aureus; Fungus: Trichoderma harzianum* | Disk diffusion; Luria-Bertani (LB) Agar | 12 h | Nguyen et al., 2018 | Vietnam |
| Cynodon *(Cynodon dactylon)* | 10 g powder boiled with 100 mL distilled water for 1 h; extract and HAuCl₄·3H₂O (1 mmol L⁻¹) ratios: 2:1, 5:1, 10:1, 20:1, 30:1, 2 h, 1200 rpm, 90 °C | UV-Vis, FT-IR, XRD, SEM, TEM | 100, 75, 50, 25 μg mL⁻¹; Ciprofloxacin (100 μg mL⁻¹) | *Enterobacter cloacae, Staphylococcus haemolyticus, Staphylococcus petrasii subsp. pragensis, Bacillus cereus* | Disk diffusion; Mueller-Hinton Agar | 24 h | Vinayagam et al., 2021 | South Korea |
| Black ginseng *(Panax Ginseng)* | 5 g crushed roots in 50 mL H₂O, boiled at 60 °C for 20 min and cooled; extract and HAuCl₄·3H₂O (1 mmol L⁻¹) ratios: 2:8, 5:5, 7:3, 8:2, incubated in sunlight for 2 h | UV-Vis, FE-TEM, EDX, XRD, FTIR | 15, 30, 55 μg mL⁻¹ | *Escherichia coli, Staphylococcus aureus* | Disk diffusion; Mueller-Hinton Agar | 24 h | Wang et al., 2016 | South Korea |
| Gold Root *(Rhodiola rosea)* | 5 mL ginseng in 25 mL sterile distilled water; 1:1 volumetric ratio (extract: HAuCl₄·3H₂O 1 mmol L⁻¹), monitored at room temperature, 16,000 rpm for 15 min and air⁻dried | AFM, UV-Vis, FTIR, Zeta Potential, TEM, MALDI-TOF | 6.25–400 μg mL⁻¹ | *Pseudomonas aeruginosa and Escherichia coli biofilms* | MIC; Mueller-Hinton Broth | 24 h | Singh et al., 2018 | Denmark |
| Shishiudo *(Angelica pubescens)* | 10 g rhizome ground and boiled 30 min with 100 mL sterile water; 1:1 volumetric ratio (extract: HAuCl₄·3H₂O 1 mmol L⁻¹), monitored at room temperature | UV-Vis, FE-TEM, EDX, XRD, SAED, DLS, FTIR | 500, 1000, 1500 μg mL⁻¹; Neomycin 30 μg | *Escherichia coli, Staphylococcus aureus, Pseudomonas* | Disk diffusion; Mueller-Hinton Agar | 24 h | Markus et al., 2017 | South Korea |
| Black ginseng *(Panax Ginseng)* | 5 g root powder in 100 mL distilled water and autoclaved 30 min at 100 °C to obtain aqueous root extract. HAuCl₄·3H₂O (1 mmol L⁻¹) at 1, 3, 5, 7, 9 mmol L⁻¹; extract solutions at 10, 30, 50, 70, 90 % (v/v); temperatures 40–100 °C, pHs 2, 4, 6, 8, 12 | UV-Vis, FE-TEM, EDX, XRD | 100 μg mL⁻¹; novobiocin and lincomycin | *Bacillus anthracis, Vibrio parahaemolyticus, Bacillus cereus* | Disk diffusion; Mueller-Hinton Agar | 24 h | Singh et al., 2016 | South Korea |

* Thermogravimetric analysis (TGA), Gas chromatography-mass spectrometry (GC-MS/MS) and High performance liquid chromatography/ultraviolet-visible (HPLC/UV-VIS), Electron diffraction (SAED), X-ray diffraction (XRD), Dynamic light scattering (DLS), Energy dispersive spectroscopy (EDX), Energy dispersive spectroscopy (EDS), Fourier transform infrared spectroscopy (FTIR), Raman spectroscopy (Raman), Ultraviolet-visible spectroscopy (UV-Vis), Field emission splitting electron microscopy (FE-SEM), Transmission electron microscopy (TEM), High resolution transmission electron microscopy (HRTEM), Scanning electron microscopy (SEM), Zeta Potential (Zeta).

Table 3. Studies on the synthesis of green gold nanoparticles from leaf extract, including plant species, synthesis methods, characterization techniques, applied concentrations, bacterial strains tested, exposure time, and authors.

| **Species** | **Synthesis Method** | **Methods Used for NP Characterization** | **NP Concentrations Used** | **Description of Bacteria** | **Experimental Design and Culture Medium** | **Exposure Time** | **References** | **Country** |
| --- | --- | --- | --- | --- | --- | --- | --- | --- |
| Jamelão *(Syzygium cumini)* | 10 g of leaves in distilled water and filtered; salt and plant extract concentration, temperature, reaction time, and pH | UV-Vis, TEM, XRD, and FT-IR | 100, 150, 250, 350, 450, and 500 μg mL⁻¹ | *Staphylococcus aureus,* *Acinetobacter baumannii, Escherichia coli, Pseudomonas aeruginosa, Enterococcus faecalis, Klebsiella pneumoniae, and Proteus vulgaris* | Disk diffusion; Mueller-Hinton Agar. Minimum Inhibitory Concentration (MIC); Luria-Bertani (LB) broth | 24 h | Diksha et al., 2023 | India |
| Graviola *(Annona muricata)* | 20 g of leaves in 150 mL deionized water, boiled for 20 min, and filtered; 1 mL of extract added to 10 mL HAuCl₄ 1 mmol L⁻¹, stirred at room temperature until color change | UV-Vis, TEM, and FT-IR | 2 and 4 mg L⁻¹ | *Staphylococcus aureus, Enterococcus faecalis, Klebsiella pneumoniae,* *Clostridium sporogenes* | Disk diffusion; Nutrient Agar | 28 h | Folorunso et al., 2019 | Nigeria |
| Maple *(Acer pentapomicum)* | 15–20 g of dried leaf powder in 150 mL deionized water, boiled 20 min, filtered; 1 mL of aqueous leaf extract with varying proportions of 1 mmol L⁻¹ gold chloride solution for 24 h | UV-Vis, TEM, XRD, and SEM | 1 and 2 mg μL⁻¹ | *Staphylococcus aureus, Pseudomonas aeruginosa, Klebsiella pneumoniae, Xanthomonas, Escherichia coli, Citrobacter freundi, Bacillus subtilis* | Disk diffusion; Nutrient Agar | 24 h (bacteria); 48–96 h (fungi) | Khan et al., 2018 | Pakistan |
| Papaya *(Carica papaya)* | 20 g extracted using Soxhlet with 150 mL sterile distilled water; 1 mmol L⁻¹ HAuCl₄ with varying extract concentrations (CP, CR, CPCRM) 5–200 μL at 60 °C for 5 min | HRTEM and FTIR | 10 mg mL⁻¹ of each solution | *Staphylococcus aureus, Escherichia coli, Bacillus subtilis, Proteus vulgaris* | Disk diffusion; Mueller-Hinton Agar. MIC; Mueller-Hinton broth | 24 h | Muthukumar et al., 2016 | India |
| Castor *(Ricinus communis)* | 1 g dry substrate in 100 mL 70 % acetone (stock solution 1%); 1 mL HAuCl₄·3H₂O 1 mmol L⁻¹ with 99 mL stock solution of 1 % extract | Extract: GC-MS/MS chromatography, HPLC/UV-Vis; RcExt-AuNPs: UV-Vis, SEM, and FT-IR | 100 μg mL⁻¹ | *Strains: Escherichia coli,* *Proteus mirabilis, Shigella flexneri, Staphylococcus aureus; Fungus: Candida albicans* | Disk diffusion; Nutrient Agar | 24 h | Ghramh et al., 2019 | Saudi Arabia |
| Midnapore Creeper *(Rivea hypocrateriformis)* | 10 g plant material in 200 mL distilled water, microwave irradiated (700 W, 2.45 GHz); 20 mL extract added to 50 mL 1 mmol L⁻¹ HAuCl₄ solution and similarly irradiated | UV-Vis, TEM, XRD, FT-IR, and EDAX | 25, 50, 75, 100 μg mL⁻¹ | *Strains: Klebsiella pneumoniae, Staphylococcus aureus, Bacillus subtilis, Pseudomonas aeruginosa, Escherichia coli; Fungi: Candida albicans,* *Trichophyton rubrum, Chrysosporium indicum* | Disk diffusion; Mueller-Hinton Agar | 24 h | Godipurge et al., 2016 | India |
| Indian jujube *(Ziziphus zizyphus)* | 10 g leaves in 100 mL bidistilled water, boiled 5 min; 5 mL extract added to 45 mL HAuCl₄ 1 mmol L⁻¹ until color change | TEM, SEM, AFM, XRD, UV-Vis, EDX, and TGA | 10, 25, 50, 100, 250, 500, 1000 μg mL⁻¹ | *Klebsiella pneumoniae, Staphylococcus aureus, Bacillus subtilis, Pseudomonas aeruginosa, Escherichia coli, and Xanthomonas campestres* | Disk diffusion; Mueller-Hinton Agar | 24 h | Aljabali et al., 2018 | Jordan |
| Princess ear *(Pergularia daemia)* | 10 g dried leaves in 100 mL deionized water; leaf extracts obtained at 0, 30, 60, 90, 120 min, treated with 100 mL 1 mmol L⁻¹ HAuCl₄ solution | HRTEM, FESEM, XRD, UV-Vis | 100, 200, 300 μL (1 mg mL⁻¹) | *Escherichia coli, Pseudomonas aeruginosa, Bacillus subtilis* | Disk diffusion; Mueller-Hinton Agar | 48 h | Rajendran et al., 2017 | India |
| Starfruit *(Terminalia arjuna)* | 10 g fruit in 100 mL bidistilled water, boiled 50–60 °C for 5 min; 1 mL extract added to 100 mL 1 mmol L⁻¹ HAuCl₄ at room temperature for 15 min | UV-Vis, TEM, EDX | 100 μL (20 mg mL⁻¹) | *Staphylococcus aureus, Pseudomonas aeruginosa,* *Salmonella typhimurium* | Disk diffusion; Nutrient Agar | 24 h | Dudhane et al., 2019 | India |
| Carnivorous plant *(Nepenthes khasiana)* | 20 g in 200 mL autoclaved bidistilled water, boiled 30 min, filtered; 25 mL extract added to 225 mL 1 mmol L⁻¹ gold chloride solution for 3 h under gentle stirring | UV-Vis, TEM, SEM, XRD, FT-IR | 5 mg mL⁻¹ | *Strains: Escherichia coli,* *Bacillus sp.; Fungi: Aspergillus niger, Candida albicans* | Disk diffusion; Nutrient Agar | 24 h (bacteria); 48 h (fungi) | Bhau et al., 2015 | India |
| Desert rose *(Justicia glauca)* | 1 g fresh leaves boiled 15 min in 300 mL sterile distilled water; 1 mmol L⁻¹ HAuCl₄·3H₂O mixed 1:1 with extract at room temperature for 10 min | UV-Vis, TEM, SEM, XRD, FT-IR, EDAX | 50, 25, 12.5, 6.25, 3.125, 1.56, 0.78 μg mL⁻¹; standard antibiotics azithromycin and clarithromycin 100 μg mL⁻¹ | *Bacteria:* *Micrococcus luteus, Bacillus subtilis, Staphylococcus aureus, Streptococcus mutans, Lactobacillus acidophilus, Escherichia coli, Pseudomonas aeruginosa; Fungi: Saccharomyces cerevisiae, Candida albicans* | Minimum inhibitory concentration; Nutrient broth | 24 h | Prakash et al., 2017 | India |
| Camellia *(Camellia japonica L.)* | 5 g finely cut dried leaves with 200 mL water for 10 min, filtered; 10 mL extract with 50 mL 0.5 mmol L⁻¹ gold solution, stirred at room temperature 40 min | UV-Vis, TEM, SEM, XRD, FT-IR | 100, 150, 200 μg mL⁻¹ | *Strains: Bacillus subtilis, Staphylococcus aureus, Streptococcus faecalis, Klebsiella pneumoniae, Pseudomonas aeruginosa, Escherichia coli; Fungus: Candida albicans* | Disk diffusion; Nutrient Agar | 24 h | Sharma et al., 2019 | Taiwan |
| Chita plant *(Alternanthera bettzickiana)* | 10 g fine powder in 100 mL bidistilled water at 80 °C for 10 min in water bath; 5–20 mL of aqueous gold solution heated to 80 °C for 10 min | UV-Vis, TEM, SEM, XRD, FT-IR, EDX | 10, 20, 30, 40 μL (20 mg mL⁻¹) | *Bacillus subtilis, Staphylococcus aureus**, Salmonella typhi, Pseudomonas aeruginosa, Micrococcus luteus, Enterobacter aerogenes* | Disk diffusion; Nutrient Agar | 24 h | Nagalingam et al., 2018 | India |
| Dandelion *(Tragopogon dubius)* | 10 g in 100 mL distilled water, boiled 5 min; 1 mL leaf extract added to 10 mL 1 mmol L⁻¹ HAuCl₄ solution at room temperature for 24 h | UV-Vis, TEM, SEM, XRD, FT-IR | 2.5, 5, 7.5, 10 mg mL⁻¹ | *Klebsiella pneumoniae, Bacillus cereus, Escherichia coli, Staphylococcus aureus* | Disk diffusion; Nutrient Agar | 24 h | Layeghi-ghalehsoukhteh et al., 2018 | Iran |
| Oregano *(Origanum vulgare)* | 2 g with 20 mL water for 30 min at 60 °C; 20 mL HAuCl₄ (1 mmol L⁻¹) with 0.25–2 mL plant extract at room temperature until color change | UV-Vis, TEM, FT-IR, Raman | 1.23, 2.4, 4.7, 6.9, 9 mg mL⁻¹ | *Staphylococcus aureus,* *Listeria monocytogenes, Escherichia coli, Salmonella typhimurium* | Disk diffusion; Mueller-Hinton Agar | Overnight | Benedec et al., 2018 | Romania |
| Wild fennel *(Nigella arvensis)* | 10 g powder in 200 mL boiling water for 15 min, filtered; 10 mL extract with 90 mL 1 mmol L⁻¹ HAuCl₄ solution until color change | UV-Vis, TEM, XRD, FT-IR | 100 μL (disk), 50 μL (MIC) (5 mg mL⁻¹) | *Staphylococcus epidermidis, Bacillus subtilis, Staphylococcus aureus, Escherichia coli,* *Serratia marcescens, Pseudomonas aeruginosa* | Disk diffusion; Mueller-Hinton Agar; MIC (Mueller-Hinton broth) | 24 h | Chahardoli et al., 2018 | Iran |
| Sambacaetá *(Dracocephalum kotschyi)* | 10 g fine powder in 200 mL water, boiled 15 min; 10 mL extract with 90 mL 1 mmol L⁻¹ HAuCl₄ solution for 10 min at room temperature | UV-Vis, TEM, FESEM, XRD, FT-IR, EDX | 50 μL (5 mg mL⁻¹) | *Staphylococcus epidermidis, Bacillus subtilis, Staphylococcus aureus, Escherichia coli, Serratia marcescens, Pseudomonas aeruginosa* | MIC; Nutrient broth | 24 h | Chahardoli et al., 2018 | Iran |
| Starfruit *(Terminalia arjuna)* | 10 g leaves in 100 mL bidistilled water, boiled 50–60 °C for 5 min, filtered; 1 mL extract with 100 mL 1 mmol L⁻¹ HAuCl₄ at room temperature for 15 min | UV-Vis, TEM, XRD, FT-IR, DLS, Zeta Potential | 500 μmol mL⁻¹, 1000 μmol mL⁻¹ (1 mg mL⁻¹) | *Staphylococcus aureus, Klebsiella pneumoniae, Proteus vulgaris* | Disk diffusion; Nutrient Agar | 24 h | Gopinath et al., 2014 | India |
| Glory tree *(Clerodendrum trichotomum)* | 15 g powdered leaves combined with 100 mL water at 50–60 °C for 15 min; 10–20 g HAuCl₄ in 100 mL extract for green synthesis of AuNPs (Au³⁺ concentration 300 mmol L⁻¹), stirred 80 min at 65 °C | TEM, XRD, EDX | 666 μg mL⁻¹ | *Klebsiella pneumoniae, Staphylococcus aureus* | MIC; Mueller-Hinton broth | — | Shakoor et al., 2025 | Saudi Arabia |
| Wild Rue *(Peganum harmala L.)* | 4 g of powder in 400 mL distilled water for 2 weeks. Volumetric ratios of 2:1, 3:1, 4:1, 6:1, 7:1 (HAuCl₄ 1 mmol L⁻¹ : extract) at room temperature until color change | UV-Vis, TEM, XRD, FT-IR | 5; 3.3; 2.5; 1.6; 1.43 μg mL⁻¹ | *Bacteria: Bacillus subtilis, Staphylococcus aureus, Escherichia coli, Pseudomonas aeruginosa, Salmonella typhi; Fungi: Candida albicans, Aspergillus niger, Penicillium notatum* | Disk diffusion; Nutrient agar | 24 h | Ullah et al., 2024 | Pakistan |
| Tiger Claw *(Martynia annua)* | 1 g leaves in 100 mL water; 5 mL extract with 5 mL of 1 mmol L⁻¹ HAuCl₄ in dark until color change | UV-Vis, SEM, XRD, DLS, Zeta Potential | 6.25, 12.5, 25, 50, 100 μg mL⁻¹ | *Escherichia coli, Staphylococcus aureus, Streptococcus sp, Bacillus subtilis, Enterococcus faecalis* | Minimum Inhibitory Concentration (MIC); Mueller-Hinton broth | 20 h | Palaniswamy et al., 2024 | India |
| Geranium *(Pelargonium graveolens)* | Volume of HAuCl₄ added to 50 mL extract to reach 250 μg mL | UV-Vis, TEM, FESEM, XRD, FT-IR, EDS | 0.2 mL of agent in concentrations 0.06, 0.12, 0.25, 0.5 mg mL⁻¹ | *Bacteria: Streptococcus mutans; Fungi: Candida albicans* | Agar well diffusion; Mueller-Hinton | 24 h | Asker et al., 2024 | Iraq |
| Sapodilla *(Manilkara zapota)* | 6 mL de HAuCl₄·3H₂O em 1 mL de extrato aquoso agitada no escuro por 10 minutos à temperatura ambiente. A cor da solução mudou imediatamente para púrpura. | UV-Vis | 50, 33.3, 1.67 mg mL⁻¹ | *Escherichia coli, Salmonella typhi, Staphylococcus aureus, MRSA* | Agar well diffusion; Mueller-Hinton | 24 h | Hutagalung et al., 2024 | Indonesia |
| Sweet Violet *(Viola odorata)* | 1:1 to 1:50 (salt: extract 0.3 g mL), 1 h at 100 °C | UV-Vis, SEM | 300 mg mL⁻¹ | *Bacteria: Staphylococcus aureus, Salmonella typhimurium, Pseudomonas aeruginosa, Escherichia coli; Fungi: Candida albicans, Aspergillus niger* | Agar well diffusion; Nutrient agar | 24 h | Ridwansyah et al., 2024 | Pakistan |
| Blackberry *(Rubus spp.)* | 460 g mol L⁻¹ extract with HAuCl₄·3H₂O 1 mmol L⁻¹, 1:1 molar ratio at room temperature until color change | UV-Vis, SEM, XRD, EDS, DLS, Zeta Potential | Same as synthesis | *Bacillus subtilis, Staphylococcus aureus, Escherichia coli, Proteus vulgaris, Pseudomonas aeruginosa, Klebsiella pneumoniae, Listeria monocytogenes; Candida albicans; Amoxicillin + Clavulanic acid 20/10 μg* | Disk diffusion; Mueller-Hinton agar | 24 h | Tasić et al., 2024 | Serbia |
| Suren *(Toona sureni (Blume) Merr.)* | 10 mg mL⁻¹ extract in HAuCl₄ 0.1 mmol L⁻¹, ratio 1:19 (v/v); 900 W microwave irradiation for heating | UV-Vis, TEM, FESEM, XRD, FT-IR, EDS, DLS, Zeta Potential | 100 μL (15.6–62.5 μg mL⁻¹) | *Staphylococcus epidermidis, Propionibacterium acnes, Staphylococcus aureus, Escherichia coli, Pseudomonas aeruginosa MRSA* | MIC; Brain Heart Infusion (BHI) | 24 h | Lestari et al., 2024 | Indonesia |
| Castor *(Ricinus communis)* | 20 g leaves boiled with 200 mL distilled water at 70 °C for 1 h; 2 mL extract to 18 mL of 1 mmol L⁻¹ HAuCl₄·3H₂O for 15 min | UV-Vis, TEM | 11 mg mL⁻¹ | *Bacteria: Bacillus subtilis, Pseudomonas aeruginosa; Fungi: Candida albicans*,* *Aspergillus fumigatus* | MIC; Mueller-Hinton broth | 24 h | Parmar et al., 2024 | India |
| Dancing Plant *(Codariocalyx motorius)* | 2 g leaves in 100 mL water, heated and filtered; 20 mL of 1 mmol L⁻¹ HAuCl₄ solution in 2:8 ratio (v/v) with extract; dried at 100 °C for 10 min | UV-Vis, TEM, FESEM, XRD, FT-IR, EDS, DLS, Zeta Potential | 10 μL (125 μg mL⁻¹) | *Bacteria: Escherichia coli, Staphylococcus aureus; Fungi: Aspergillus flavus, Candida albicans* | Disk diffusion; Mueller-Hinton agar | 24 h | Deivanathan et al., 2024 | India |
| Sugar Apple *(Annona squamosa L.)* | 5 g leaves in 100 mL deionized water at 60 °C for 10 min, filtered; 5 mL to 25 mL of 1 mmol L⁻¹ HAuCl₄ at room temperature, 350 rpm, 20 h | TEM, UV-Vis, XRD, FT-IR, DLS | 1.953, 3.906, 7.8125, 15.625, 31.25, 62.5, 125, 250, 500, 1000, 2000 μg mL⁻¹ | *Escherichia coli, Staphylococcus aureus* | MIC; Mueller-Hinton broth | overnight | Jibrin et al., 2024 | Nigeria |
| Caribbean Jasmine *(Plumeria pudica)* | 20 g chopped leaves with 200 mL deionized water, heated 105°C for 2 h; different volumes of 0.5–2.5 mL HAuCl₄ solution in 5 mL extract; 50°C, 20 min | UV-Vis, TEM, XRD, FT-IR, EDS, PL, Zeta Potential | 0.1; 0.2; 0.3; 0.4; 0.5 mg mL⁻¹ | *Bacillus subtilis, Escherichia coli, Pseudomonas aeruginosa* | Disk diffusion; Mueller-Hinton agar | overnight | Anamika et al., 2025 | India |
| Mozambique Flower *(Elytraria acaulis)* | 5 g leaf in 250 mL deionized water at 60 °C for 20 min; 34 mL of 1 mmol L⁻¹HAuCl₄ aqueous solution with 3 mL extract incubated overnight | UV-Vis, TEM, XRD, FT-IR, FESEM, PL | 10 mg mL⁻¹ | *Escherichia coli, Staphylococcus aureus* | Disk diffusion; Mueller-Hinton agar | 18 h | Sathiyaraj et al., 2025 | India |
| King of Bitters *(Andrographis paniculata)* | 10 g powdered leaves in 100 mL water; 10 mL extract added to 90 mL HAuCl₄; pH 7.0, 70 °C until color change | TEM, UV-Vis, XRD, FT-IR | 10 mg mL⁻¹ | *Salmonella typhi, Bacillus cereus, Pseudomonas aeruginosa, Escherichia coli* | Disk diffusion; Mueller-Hinton agar | overnight | Chandran et al., 2025 | India |
| Showy Violet *(Viola betonicifolia)* | 20 g leaves in 150 mL water; 1 mmol L⁻¹ HAuCl₄·3H₂O added to 25 mL leaf extract at 40 °C for 60 min | TEM, UV-Vis, XRD, FT-IR, EDS, DLS | 250 μg mL⁻¹ | *Bacillus subtilis, Staphylococcus aureus, Escherichia coli, Pseudomonas aeruginosa* | Disk diffusion; TSB & RPMI agar | 24 h | Wang et al., 2021 | China |
| Chinese Bellflower *(Platycodon grandiflorus)* | 10 g dried leaves in 100 mL distilled water; 30 mL of 1 mmol L⁻¹ HAuCl₄·3H₂O with 10 mL extract, incubated at different temperatures (20, 37, 50 °C); optimized with 2.5, 5, 10 mL extract at 50 °C | TEM, UV-Vis, XRD, FT-IR, EDS, DLS | 5, 10, 15, 20 μg mL⁻¹ | *Escherichia coli, Bacillus subtilis* | Disk diffusion; LB agar | 24 h | Anbu et al., 2020 | South Korea |
| White Wormwood and Mulberry *(Artemisia herba-alba and Morus alba)* | 10 g powder in 100 mL; 10 μL extract with 90 μL of 1 mmol L⁻¹ HAuCl₄ at 25 °C for 20 min | UV-Vis, SEM, TEM | 10, 20, 40, 60, 80 μg mL⁻¹ | *Escherichia coli, Salmonella typhi* | Agar well diffusion; Mueller-Hinton | 24 h | Abdalhamed et al., 2021 | Egypt |
| Paradise Tree *(Simarouba glauca)* | 1 g powdered leaves in 50 mL distilled water, stirred 1 h; extract: salt ratios 0.3:27, 0.6:24, 0.9:21, 1.2:1.8 at room temperature until color change | TEM, UV-Vis, XRD, FT-IR, EDS | 2, 4, 6, 8 mg mL⁻¹ | *Staphylococcus aureus, Streptococcus mutans, Bacillus subtilis, Escherichia coli, Proteus vulgaris, Klebsiella pneumoniae* | Disk diffusion; Mueller-Hinton agar | 24 h | Thangamani et al., 2019 | India |
| Mast Tree *(Polyalthia longifolia)* | 10 g dried green leaves in 100 mL boiling water; 10 mL of extract added to 100 mL aqueous solution of 1 mmol L⁻¹ HAuCl₄ ×H₂O at room temperature, stirred for 20 min | TEM, SEM, UV-Vis, XRD, FT-IR, EDS | 100 μg mL⁻¹ each | *Escherichia coli, Bacillus subtilis* | Disk diffusion; Mueller-Hinton agar | 24 h | Mumtaz et al., 2022 | Saudi Arabia |
| Gundelia *(Gundelia tournefortii L.)* | 10 g dried green leaves in 100 mL boiling water; 50 mL extract + 75 mL HAuCl₄·3H₂O 3 mmol L⁻¹ at 45 °C until color change | FT-IR, FESEM, EDS, UV-Vis | AuNPs 40 mg mL⁻¹; fluconazole (fungus), vancomycin (Gram+), colistin (Gram-) 128 mg mL⁻¹ | *Bacteria: Escherichia coli, Pseudomonas aeruginosa, Bacillus subtilis, Staphylococcus aureus; Fungi: Candida albicans* | MIC; BHI | 24 h | Keskin et al., 2022 | China |
| Sambacaetá *(Dracocephalum kotschyi)* | 5 g leaves boiled with 75 mL deionized water; 10 mL HAuCl₄ 1 mmol L⁻¹ added to 2 mL extract, stirred vigorously for 2 min | EM-SEAD, SEM-EDAX, XRD, Zeta Potential, DLS, FT-IR | 11 mg mL⁻¹ | *Bacillus subtilis, Staphylococcus aureus, Bacillus cereus, Escherichia coli, Pseudomonas aeruginosa, Proteus vulgaris* | Agar well diffusion; Mueller-Hinton | 24 h | Dorosti; Jamshidi, 2016 | Iran |
| Garlic *(Allium noeanum)* | 40 g in distilled water for 48 h at 30 °C; 10 mL of extract added to 100 mL aqueous solution of 1 mmol L⁻¹ HAuCl₄ × H₂O at room temperature, stirred for 30 min; dried in oven at 50 °C | FT-IR, UV, XRD, EDS, FE-SEM, TEM | 2, 4, and 8 mg mL⁻¹ | *Streptococcus pneumoniae, Bacillus subtilis, Staphylococcus aureus,* *Staphylococcus saprophyticus, Salmonella typhimurium, Pseudomonas aeruginosa, Shigella flexneri, Escherichia coli* | Disk diffusion; Mueller-Hinton agar | 24 h | Shahriari et al., 2019 | Iran |
| Arabian Jasmine *(Jasminum sambac)* | 10 g leaves in 200 mL distilled water; 10 mL extract to 50 mL of 1 mmol L⁻¹ aqueous HAuCl₄; microwave irradiation at maximum power (700 W, 2.45 GHz); vacuum oven at 70–80 °C for ~12 h to dry | XRD, EDX, FESEM, FT-IR | 1 mg mL⁻¹ | *Staphylococcus aureus, Salmonella typhimurium, Pseudomonas aeruginosa, Escherichia coli* | Disk diffusion; Mueller-Hinton agar | 24 h | Yallappa et al., 2015 | India |
| Qinghaosu *(Artemisia annua)* | 4 g leaves in 200 mL deionized water, boiled for 5 min; 5 mL extract added to 50 mL saline solutions (5 mmol L⁻¹), stirred 10 min at room temperature; dried at 40 °C for 2 h | UV-Vis, XRD, TEM, EDX, FT-IR, TGA | 1000, 100, 10, 1 μg mL⁻¹ | *Escherichia coli, Pseudomonas aeruginosa**, Enterobacter aerogenes, Bacillus cereus, Staphylococcus aureus* | Disk diffusion; Mueller-Hinton agar | 24 h | Basavegowda et al., 2014 | South Korea |
| Boldo *(Coleus forskohlii)* | 100 mg in 100 mL Milli-Q water, filtered; 1–5 mL extract added to 10 mL 1 mmol L⁻¹ HAuCl₄ at 80 °C for 15 min | UV-Vis, XRD, TEM, FT-IR | 15, 25, 35 μL (0.5 mg mL⁻¹) | *Escherichia coli, Pseudomonas aeruginosa, Staphylococcus aureus* | Disk diffusion; Mueller-Hinton agar | 24 h | Naraginti et al., 2014 | India |
| Olive *(Olea europaea)* | 10 g powdered leaves in 100 mL distilled water, boiled 5 min, filtered; 25 mL extract added to 1 mmol L⁻¹ HAuCl₄·3H₂O, heated at 80 °C for 65 min; centrifuged 15 min at 15,000 rpm, washed thrice with deionized water, dried at 70 °C | UV-Vis, XRD, TEM, EDX | 500 μg/mL; ciprofloxacin | *Staphylococcus aureus, Klebsiella pneumoniae* | Agar well diffusion; Mueller-Hinton agar | 24 h | Sellami et al., 2022 | Tunisia |
| King of bitters *(Andrographis paniculata)* | 50 mL 1 mmol L⁻¹ HAuCl₄ solution heated at 80 °C for 10 min, stirred 15 min at 90 °C, 5 mL leaf extract (10 % w/v) added dropwise; dried at 50 °C | UV-Vis, SEM, TEM, FT-IR | 50, 100 μg mL⁻¹ | *Escherichia coli, Staphylococcus aureus, Enterococcus faecalis; Fungi: Aspergillus niger, Candida albicans* | Agar well diffusion; Mueller-Hinton agar | 24 h | Paramasivam et al., 2023 | India |
| Bird Plum *(Sageretia thea)* | 500 g of leaf powder was in 1500 mL of ethanol for 2 weeks. 1 mL of leaf extract solution with 1 mmol L⁻¹ of HAuCl₄ in different proportions (1:1 to 1:10) in volume and stirred for 30 minutes at room temperature until color change | UV-Vis, XRD, TEM, SEM | 100, 200 μg mL⁻¹ | *Klebsiella pneumoniae, Staphylococcus aureus, Bacillus subtilis* | Agar well diffusion; Mueller-Hinton agar | 24 h | Shah et al., 2021 | Pakistan |
| Parsley *(Petroselinum crispum)* | 20 g dried leaves in 200 mL deionized water at 90 °C for 20 min; 2.5, 5, 10, 20 mL extract (AuNPs A–D), keeping HAuCl₄·H₂O molarity 1 mmol L⁻¹ until color change | UV-Vis, XRD, TEM, EDX | 200 μg mL⁻¹ | *Bacillus subtilis, Enterococcus faecalis, Escherichia coli,* *Enterobacter ludwigii* | Agar well diffusion; Mueller-Hinton agar | 48 h | El-borady et al., 2020 | Egypt |
| Wheat *(Triticum aestivum)* | 20 g chopped leaves in 100 mL HPLC purified water; 10 mL hot extract added to 90 mL 1 mmol L⁻¹ HAuCl₄.XH₂O; boiled 10 min at 90 °C | UV-Vis, FT-IR, DLS, XRD, HRTEM | 0.5, 1.0, 2.0, 3.0 mg mL⁻¹ | *Klebsiella pneumoniae, Salmonella typhimurium, Enterobacter aerogenes, Escherichia coli, Micrococcus luteus, Staphylococcus aureus, Staphylococcus epidermidis, Streptococcus mutans* | Agar well diffusion; Mueller-Hinton agar | 24 h | Pahal et al., 2022 | India |
| Almonds *(Amygdalus communis)* | \| 10 g in 100 mL water; 30 mL extract added to 120 mL HAuCl₄ (20 mmol L⁻¹) at 1:4 ratio; 10, 15, 30, 45, 60 min at room temperature \| \| --- \| | Extract: LC-ESI-MS/MS; NPs: XRD, TEM, FT-IR, FESEM, UV-Vis | 5–0.156 mg mL⁻¹ | *Staphylococcus aureus, Bacillus subtilis, Escherichia coli, Pseudomonas aeruginosa; Fungi: Candida albicans* | MIC in BHI | 24 h | Baran et al., 2023 | Turkey |
| Imperial cassia *(Cassia fistula)* | 100 mL of deionized water was poured over 15 g of cut leaves and boiled at 90 °C for 45 min and filtered. 10 mL of HAuCl₄.3H₂O aqueous solution (1 mmol L⁻¹) was mixed dropwise with 10 mL of extract and shaken vigorously at 450 rpm for 6 min until color change. | UV-Vis, TEM, EDX, FT-IR, DLS and Zeta Potential | 0,15 mg mL⁻¹ | *Escherichia coli* | MIC; Mueller-Hinton broth | 24 h | Beg et al., 2020 | India |
| Albizia *(Albizia amara Roxb.)* | 1 g leaf powder in 100 mL deionized water, boiled 30 min, filtered; 8 mL aqueous extract to 60 mL HAuCl₄ 1 mmol L⁻¹, orbital shaker at room temperature; overnight drying | FESEM, EDX, XRD, FT-IR, UV-Vis | 20, 40, 60, 80, 100 μg mL⁻¹ | *Staphylococcus aureus* | Agar well diffusion; Mueller-Hinton agar | 24 h | Balasubramani et al., 2017 | India |
| Bee Sting Bush *(Azima tetracantha Lam.)* | 20 g fine powder in 200 mL Milli-Q water, boiled 10 min at 60 °C; 10 mL extract with 90 mL HAuCl₄ (1 mmol L⁻¹) at 60 °C for 10 min | DLS, SEM, UV-Vis, FT-IR, XRD | 15, 30 μL (10 mg mL⁻¹) | *Aeromonas liquefaciens, Enterococcus faecalis, Micrococcus luteus, Salmonella typhimurium; Fungi: Candida albicans,* *Cryptococcus sp., Microsporum canis, Trichophyton rubrum* | Agar well diffusion; Mueller-Hinton agar | 24 h | Hariharan et al., 2016 | India |
| Pecah beling *(Strobilanthes crispa)* | 2 g leaf powder in 100 mL deionized water, boiled 15 min at 100 °C; 10 mL HAuCl₄ (1 mmol L⁻¹) with 5 mL extract at room temperature for 1 h | UV-Vis, FT-IR, HRTEM, XRD | 6.6 g mL⁻¹ | *Escherichia coli, Staphylococcus aureus* | Agar well diffusion; Mueller-Hinton agar | 24 h | Samsulkahar et al., 2023 | Malaysia |
| River Willow *(Combretum erythrophyllum)* | 5 g dried leaves in 100 mL deionized water at 90 °C for 1 h; different extract volumes (0.5, 1.0, 1.5, 2.0 mL) to 25 mL HAuCl₄ 1 mmol L⁻¹ at room temperature until color change | UV-Vis, FT-IR, HRTEM, XRD | 2000, 1000, 500, 250, 125, 62.5 μg mL⁻¹ | *Staphylococcus epidermidis, Staphylococcus aureus,* *Mycobacterium smegmatis, Proteus mirabilis, Escherichia coli, Klebsiella pneumoniae,* *Klebsiella oxytoca* | MIC; Mueller-Hinton broth | 24 h | Fanoro et al., 2021 | South Africa |
| Smoke Tree *(Cotinus coggygria Scop.)* | 20 g chopped plant material extracted with 350–400 mL ethanol for 3 h using magnetic stirrer at room temperature; different volumes (0.1, 0.2, 0.5 mL) of aqueous extract added to chloroauric acid solution, final volume 25 mL, stirred at 250 rpm, 25 °C until color change | UV-Vis, FT-IR, Fluorescence, TEM | 30 μL (0.2 mg mL⁻¹) | *Escherichia coli, Staphylococcus aureus* | Agar well diffusion; Mueller-Hinton agar | 24 h | Ozgur et al., 2021 | Turkey |
| Chinese Mulberry *(Cudrania tricuspidata)* | 30 g leaves in 100 mL deionized water, boiled 30 min, cooled; 100 mL extract added to 1 L (1 mmol L⁻¹) HAuCl₄ at room temperature until color change | UV-Vis, FT-IR, HRTEM, SAED, EDS, XRD | 50, 100, 150, 200 mg mL⁻¹ | *Bacillus subtilis, Pseudomonas aeruginosa* | Agar well diffusion; Mueller-Hinton agar | 24 h | Velmurugan et al., 2022 | India |
| Christmas Box *(Sarcococca saligna)* | 10 g fine powder in 50 mL distilled water for ~6 h at 40 °C; 5 mL extract to 95 mL HAuCl₄ 1 mmol L⁻¹ | UV-Vis, TEM | 10 mg mL⁻¹ | *Candida albicans; Staphylococcus aureus, Escherichia coli (comparable to amoxicillin and fluconazole)* | Agar well diffusion; Nutrient agar | 24 h | Rehman et al., 2023 | Pakistan |
| Saltwort *(Cressa cretica)* | 10 g leaf powder in 100 mL at 80 °C for 20 min, filtered; 10 mL extract with 90 mL HAuCl₄·3H₂O (1 mmol L⁻¹) at room temperature for 2 h | UV-Vis, FT-IR, TEM, SEM, XRD, EDX | 1, 2, 3 mg mL⁻¹; Chloramphenicol 10 µg | *Streptococcus pyogenes, Staphylococcus aureus, Escherichia coli, Klebsiella pneumoniae* | Agar well diffusion; Mueller-Hinton agar | 24 h | Balasubramanian et al., 2019 | South Africa |
| Kalmegh *(Andrographis paniculata)* | 4 g of leaves in 250 mL of water at 50 °C, filtered; 1.5 mL of extract in 0.5 mmol L⁻¹ HAuCl₄, 60 °C for 30 min, pH 6 | UV-Vis, FTIR, TEM, XRD, EDX | 0.75 mg/mL | *Escherichia coli, Salmonella enterica,* *Bacillus subtilis subsp. spizizenii, Staphylococcus aureus* | Disc diffusion; Mueller-Hinton agar | 24 h | Do Dat et al., 2023 | Vietnam |
| *Black nightshade (Solanum nigrum)* | 10 g in 100 mL distilled water at 100 °C for 30 min; HAuCl₄ (12 mL, 0.3 mmol L⁻¹) to 8 mL extract with constant stirring for 10 min until purple | UV-Vis, FTIR, TEM, XRD, EDAX | 50 μg mL⁻¹ | *Escherichia coli* | Disc diffusion; Mueller-Hinton agar | 24 h | Vijilvani et al., 2020 | India |
| Samar tree *(Haloxylon salicornicum)* | 2 g powder in 100 mL distilled water, boiled at 80 °C in a water bath for 15 min; 1 mL plant extract with 9 mL 1 mmol L⁻¹ HAuCl₄·3H₂O at 80 °C for 30 min until reddish-pink color stabilized | UV-Vis, FTIR, XRD, Zeta potential | 2 mg mL⁻¹ | *Staphylococcus aureus, Bacillus cereus, Escherichia coli, Klebsiella pneumoniae* | Minimum Inhibitory Concentration (MIC); Mueller-Hinton broth | 24 h | Hamida et al., 2023 | Egypt |
| Wild jasmine *(Clerodendrum inerme)* | 10 g leaf powder in 100 mL deionized water; 25 mL extract with 1 mmol L⁻¹ HAuCl₄·3H₂O at 80 °C for 65 min, dried at 70 °C after 15,000 rpm for 15 min | UV-Vis, FTIR, TEM, XRD, DLS | 250 μg mL⁻¹ (well); 40–0.781 μg mL⁻¹ (broth) | *Bacillus subtilis, Staphylococcus aureus, Klebsiella, Escherichia coli; Fungi: Aspergillus niger, Trichoderma harzianum, Aspergillus flavus* | Well diffusion; Mueller-Hinton agar; MIC; Mueller-Hinton broth | 24 h | Khan et al., 2020 | Hong Kong |
| Orchid tree *(Bauhinia purpurea)* | 25 g boiled for 20 min with 100 mL bidistilled water; extract and HAuCl₄ (1 mmol L⁻¹) in 1:10 ratio exposed to microwave radiation 800 W, 2450 MHz | UV-Vis, FTIR, TEM, XRD, EDX | 2.5 mg mL⁻¹ | *Staphylococcus aureus, Bacillus subtilis, Escherichia coli, Pseudomonas aeruginosa; Fungi: Aspergillus flavus, Aspergillus nidulans* | Well diffusion; Mueller-Hinton agar | 24–48 h | Vijayan et al., 2019 | India |
| Acacia *(Acacia baileyana)* | 10 g powder in 100 mL bidistilled water for 10 min at 80 °C, filtered; 10 mL AuCl₂, 10 mL aqueous Acacia extract, 80 mL distilled water for 1, 12, 18, 24, 48 h at room temperature | UV-Vis, TEM | 30, 60, 100 mg mL⁻¹; ampicillin | *Streptococcus mutans, Escherichia coli* | Well diffusion; Mueller-Hinton agar | — | Al Shayeb et al., 2023 | UAE |
| Indian tea *(Camellia sinensis)* | 50 g leaves in 500 mL water, infused for 24 h at 10 °C; 900 μL tea with 100 μL HAuCl₄ (10 mmol L⁻¹) at room temperature for 24 h, then air dried | UV-Vis, TEM | Read carefully | *Staphylococcus aureus, Klebsiella pneumoniae* | Antimicrobial tests on dyed cotton fabrics; bacteria grown in nutrient broth | 18 h | Onitsuka et al., 2019 | Japan |
| Pepper *(Capsicum annuum L.)* | 10 g in 250 mL water, filtered; 125 mL extract + 500 mL 5 mmol L⁻¹ HAuCl₄·3H₂O at 25 °C until color change | UV-Vis, FTIR, SEM, XRD | 25 μg mL⁻¹ | *Escherichia coli, Staphylococcus aureus, Bacillus subtilis; Fungi: Candida albicans* | MIC; Mueller-Hinton broth | Overnight | Baran et al., 2020 | Turkey |
| Eastern Indian lemongrass *(Cymbopogon flexuosus)* | 2 mL essential oil in 10 mL acetone; 7 mL oil added to 0.1 mol L⁻¹ HAuCl₄ at 40–60 °C for 2 h; 1.5 mL NaOH 0.5 mol L⁻¹ added to maintain pH | UV-Vis, FTIR, SEM, XRD | 140 mg mL⁻¹ | *Staphylococcus aureus, Escherichia coli; Fungi:* *Fusarium oxysporum* | Well diffusion; Mueller-Hinton agar | Overnight | Pathania et al., 2022 | India |
| Vernonia Cinerea *(Cyanthillium cinereum)* | 10 g crushed leaves in 100 mL bidistilled water; 5 mL aqueous extract to 45 mL HAuCl₄ (1 mmol L⁻¹) at room temp for 5 min | UV-Vis, EDX, SEM, XRD | 1, 2 mg mL⁻¹; Ampicillin, Chloramphenicol, Fluconazole 0.1 0.04 mg mL⁻¹ | *Bacillus subtilis,* *Chromobacterium violaceum, Escherichia coli, Pseudomonas aeruginosa, Staphylococcus aureus, Streptococcus pyogenes; Fungi: Aspergillus niger, Fusarium oxysporum,* *Rhizopus oryzae, Penicillium expansum* | Disc diffusion; Czapek-dox and potato dextrose agar | Overnight | Singh et al., 2021 | India |
| *S*pearmint *(Mentha spicata)* | 0.5 mL commercial essential oil + 19 mL HAuCl₄ (10 mmol mL⁻¹) at room temp for 24 h, then oven dried | Extract: GC-MS; NPs: UV-Vis, TEM, DLS, XRD, FTIR | 100, 50, 25, 12.5 μg mL⁻¹ | *Listeria monocytogenes, Salmonella Typhimurium, Staphylococcus aureus, Escherichia coli, Bacillus cereus* | Well diffusion; Mueller-Hinton agar | 24 h | Moosavy et al., 2023 | Iran |
| Gnidia *(Lasiosiphon eriocephalus)* | 10 g leaf powder in 250 mL water; 100 mL extract + 900 mL HAuCl₄·3H₂O (1 mmol L⁻¹) at 80 °C in dark until color change | UV-Vis, SEM, TEM, XRD, FTIR, DLS, Zeta potential | 10, 20, 40, 60, 80, 100 μg mL⁻¹ | *Staphylococcus aureus, Escherichia coli, Klebsiella pneumoniae, Pseudomonas aeruginosa* | MIC; Mueller-Hinton broth | 24 h | Datkhile et al., 2023 | India |
| Ground pine *(Ajuga bracteosa)* | 5 g dry powder in 250 mL deionized water at 100 °C; 4 mL HAuCl₄ + 10 mL boiled plant extract and infusion separately; water bath 40 °C for 24 h | UV-Vis, SEM, FTIR, XRD, GC-MS | 0.04 mg mL⁻¹ | *Escherichia coli, Staphylococcus aureus, Pseudomonas aeruginosa* | Well diffusion; Mueller-Hinton agar | 24 h | Raja et al., 2023 | Pakistan |
| Boldo *(Coleus forskohlii)* | 8 g leaves in 100 mL deionized water for 30 min; 0.4 mL root extract + 1 mL HAuCl₄ | Extract: GC-MS; NPs: UV-Vis, HR-TEM, PSA, FTIR, XRD | 250, 500, 750, 1000 μg mL⁻¹; tetracycline | *Proteus vulgaris, Micrococcus luteus* | Disc diffusion; Nutrient agar | 24–48 h | Dhayalan et al., 2023 | India |
| Wild mint *(Mentha longifolia)* | 84.94 mg in 250 mL bidistilled water; 0.1 g methanolic extract dissolved in 100 mL HAuCl₄, diluted to 500 mL with water at 70 °C for 3 min | UV-Vis, FTIR, TEM, XRD | 2 mg mL⁻¹ NPs; Streptomycin 2 mg mL⁻¹ | *Klebsiella pneumoniae, Staphylococcus aureus, Bacillus subtilis* | Disc diffusion; Mueller-Hinton agar | 24 h | Rauf et al., 2021 | Pakistan |
| Lemon balm *(Melissa officinalis L.)* | 5 g dried sample mixed with silica spheres in 34 mL ethanol; 10 mL extract added dropwise to 40 mL HAuCl₄ 1 mmol L⁻¹ under vigorous stirring at room temp until color change | Extract: GC-MS; NPs: UV-Vis, HR-TEM, PSA, FTIR, XRD | 29.4 mg mL⁻¹ | *Staphylococcus aureus, Bacillus cereus, Pseudomonas aeruginosa* | Disc diffusion; Mueller-Hinton agar; MIC; Mueller-Hinton broth | 24 h | Fierascu et al., 2017 | Romania |
| Eyebright *(Euphrasia officinalis)* | 50 g leaf powder in 500 mL deionized water for 30 min; 5 mL + 25 mL sterile water + HAuCl₄ to 1 mmol L⁻¹ at 65 °C until color change | FE-TEM, SAED, EDS, XRD, Zeta potential, FTIR | 1–10 μg mL⁻¹ | *Pseudomonas aeruginosa, Escherichia coli, Staphylococcus aureus**, Vibrio parahaemolyticus* | Disc diffusion; Mueller-Hinton agar | 24 h | Singh et al., 2018 | South Korea |
| Tree bean *(Parkia roxburghii)* | 1 g dried leaf powder + 100 mL 1 mmol L⁻¹ HAuCl₄ aqueous solution, stirred 12 h at room temp | UV-Vis, FTIR, XRD, TEM | 10 mg mL⁻¹ | *Staphylococcus aureus, Escherichia coli* | Disc diffusion; Mueller-Hinton agar | 24 h | Paul et al., 2016 | India |
| Plantain banana *(Musa balbisiana)* | 20 g boiled at 75 °C in 200 mL of distilled water for 1.5 h and filtered after cooling. Then, a 1 mmol L⁻¹ aqueous gold solution was prepared by dissolving 39.38 mg. 1 mL of homogeneous HAuCl₄ solution (1 mmol L⁻¹) to 15 mL of heated extract, under stirring at 350 rpm, 25 °C, 45 min until color change. | UV-Vis, FTIR, XRD, TEM, FE-SEM, EDAX, DLS | 6,7 mg mL⁻¹ | *Escherichia coli, Staphylococcus aureus* | Well diffusion; Mueller-Hinton agar | 24 h | Maji et al., 2019 | India |

*Thermogravimetric analysis (TGA), Gas chromatography-mass spectrometry (GC-MS/MS) and High performance liquid chromatography/ultraviolet-visible (HPLC/UV-VIS), Electron diffraction (SAED), X-ray diffraction (XRD), Dynamic light scattering (DLS), Energy dispersive spectroscopy (EDX), Energy dispersive spectroscopy (EDS), Fourier transform infrared spectroscopy (FTIR), Raman spectroscopy (Raman), Ultraviolet-visible spectroscopy (UV-Vis), Field emission splitting electron microscopy (FE-SEM), Transmission electron microscopy (TEM), High resolution transmission electron microscopy (HRTEM), Scanning electron microscopy (SEM), Zeta Potential (Zeta).

Table 4. Studies on the synthesis of green gold nanoparticles from flower extract, including plant species, synthesis methods, characterization techniques, applied concentrations, tested bacterial strains, exposure time, and authors.

| **Species** | **Synthesis Method** | **Methods Used for NP Characterization** | **Employed NP concentrations** | **Description of Bacteria** | **Experimental Design and Culture Medium** | **Exposure Time** | **References** | **Country** |
| --- | --- | --- | --- | --- | --- | --- | --- | --- |
| Jasmine Mango *(Plumeria alba)* | 5 g of flower powder was separately mixed with 100 mL of deionized water and filtered; 10 mL of extract was separately added to 25 mL of HAuCl₄ (1 mmol L⁻¹) at room temperature, 2 min, centrifuged at 18,000 rpm and air⁻dried | UV-Vis, TEM, XRD, Zeta Potential, FT-IR, and EDAX | 200, 300, and 400 μg mL⁻¹ | *Escherichia coli* | Diffusion disk; Mueller-Hinton agar | 24 h | Mata et al., 2016 | India |
| Marigold *(Tagetes patula L.)* | Flowers were washed, crushed, and soaked in 100 mL of distilled water heated for 5 min; 5 mL of flower extract and 45 mL of HAuCl₄ 2 mmol L⁻¹ were mixed and stored in the dark for 24 h with constant agitation at 45 °C | UV-Vis, SEM, FTIR, and EDX | 300 μg mL⁻¹ | *Pseudomonas aeruginosa, Escherichia coli, Klebsiella pneumoniae, Staphylococcus aureus,* *Xanthomonas campestris, Ralstonia solanacearum, and Erwinia amylovora* | Well diffusion method; Mueller-Hinton agar | 2 h | Hussain et al., 2025 | Pakistan |
| Red Silk Cotton *(Bombax ceiba)* | 2 g of dry mass in 80 mL of distilled water, vigorously stirred in a heater; 331 mL of 1 mmol L⁻¹ AuCl₃ aqueous solution was reduced using 1, 2, and 3 mL of extract for 30 min | UV-Vis and SEM | 75.5, 151, and 226.6 μg mL⁻¹ | *Salmonella typhi, Escherichia coli, Pseudomonas aeruginosa, Bacillus subtilis, and Staphylococcus aureus* | Diffusion disk; Mueller-Hinton agar | – | Aziz et al., 2025 | Saudi Arabia |
| Bottlebrush *(Callistemon viminalis)* | Different HAuCl₄ concentrations in aqueous extract: 1:1 to 1:8, constantly stirred at room temperature and then heated at 80 °C for 5–50 min | FE-SEM, UV-Vis, TEM, EDX, and FT-IR | 1 mg mL⁻¹ | *Escherichia coli, Staphylococcus aureus, Klebsiella pneumoniae, Pseudomonas aeruginosa, Candida albicans,* *Candida krusei, Aspergillus sp., and Trichoderma spp.* | Diffusion disk; Mueller-Hinton agar | 24 h | Khan et al., 2024 | Pakistan |
| Aconite *(Aconitum Laeve)* | 1 mL of extract at 20 mg mL⁻¹ in 1 mL of HAuCl₄ (2 mmol L⁻¹) | UV-Vis, TEM, XRD, and FT-IR | 10, 20, 40, and 80 μg mL⁻¹ | *Escherichia coli and Staphylococcus aureus* | Diffusion disk; Luria-Bertani (LB) agar | 24 h | Ahmad et al., 2024 | China |
| Rosemary *(Rosmarinus officinalis)* and Immortelle *(Helichrysum italicum)* | 25 g in 100 mL for 50 min at 80 °C; 4 mL of extracts in 1 mL of HAuCl₄·3H₂O (1 mmol L⁻¹) for 10 min at room temperature; 18,000 rpm, washed with distilled water, air⁻dried | UV-Vis, DLS, Zeta Potential, FE-SEM-EDX, and FT-IR | 1 g mL⁻¹ | *Escherichia coli, Staphylococcus aureus, and Staphylococcus epidermidis* | Minimum Inhibitory Concentration (MIC); Mueller-Hinton broth | 24 h | Onmaz et al., 2022 | Turkey |
| Mexican Poppy *(Argemona mexicana L)* | 10 g of powder in 150 mL of 2⁻propanol; 3 mL of HAuCl₄·3H₂O 1 mmol L⁻¹ in 3 mL of extract adjusted to 100 ppm at 80 °C for 30 min | UV-Vis | 100 μg mL⁻¹ | *P. aeruginosa resistant to imipenem and meropenem (IMP/MEM), Klebsiella pneumoniae, and beta-lactamase producing Escherichia coli* | Diffusion disk; Mueller-Hinton agar | 18 h | Hernández-martínez et al., 2021 | Mexico |
| Carduus *(Carduus edelbergii Rech. f.)* | 1 g of flower in 100 mL methanol, incubated 24 h at room temperature; 5 mL extract in 95 mL HAuCl₄·3H₂O for 24 h at room temperature | UV-Vis, FT-IR, SEM, XRD, and EDX | 10 mg mL⁻¹; standard antibiotic: amoxicillin | *Klebsiella aerogenes, Escherichia coli, Staphylococcus epidermidis, Staphylococcus aureus, and* *Acetobacter sp.* | Diffusion disk; Mueller-Hinton agar | 24 h | Jamil et al., 2022 | Pakistan |
| Broccoli *(Brassica oleracea L)* | 1 g of fine powder in 200 mL of 90 % ethanol at 37 °C for 12 h and filtered; different times (1, 2, 3, 6, 12, 24 h) and metal ion concentrations (10⁻² to 10⁻⁶ mmol L⁻¹) used in 1:1 volumes; 12,000 rpm for 20 min, dried at 45 °C | UV-Vis, FT-IR, SEM, XRD, and EDX | 50 μg mL⁻¹ | *Bacteria: Bacillus subtilis, Escherichia coli, Salmonella typhi, Pseudomonas aeruginosa; Fungi: Aspergillus sp.,* *Pneumocystis sp.* | Diffusion disk; Nutrient agar | 24 h | Kuppusamy et al., 2015 | Malaysia |
| African Helichrysum *(Helichrysum odoratissimum)* | 1 mL of incense (18 mg mL⁻¹ stock) in 17 mL water at 45 °C for 15 min and filtered; 300 μL of 0.1 mol L⁻¹ HAuCl₄·3H₂O added to 18 mL extract with 36 g gum arabic, reacted 15 min at room temperature | TEM, DLS, UV-Vis, TGA, XRD, and Zeta Potential | 20–25 mg mL⁻¹ | *Cutibacterium acnes* | Minimum Inhibitory Concentration (MIC); BHI broth | 72 h | De Canha et al., 2021 | South Africa |
| Stinking Herb *(Paederia foetida Linn)* | 20 g dried flowers crushed and boiled in 100 mL distilled water for 10 min; 10 mL extract added dropwise to 90 mL of 1 mmol L⁻¹ HAuCl₄ solution under constant stirring at room temperature for 8 h | UV-Vis, FTIR, XRD, and TEM | 20 mg mL⁻¹ | *Bacillus cereus, Escherichia coli, Staphylococcus aureus, and Aspergillus niger* | Diffusion disk; Nutrient agar | 24 h | Sankar Dhar et al., 2017 | India |
| Banana *(Musa acuminata colla)* | 25 g dried flowers in Soxhlet using ethanol as organic solvent, then refluxed; 10 mL extract added to 90 mL HAuCl₄·3H₂O (1.0 mmol L⁻¹) at room temperature until color change | UV-Vis, FTIR, XRD, TEM, and EDAX | 125, 250, 500, 1000 μg mL⁻¹ | *Staphylococcus aureus, Enterococcus faecalis, Escherichia coli, Salmonella typhi, Pseudomonas aeruginosa,* *Proteus mirabilis, and Klebsiella pneumoniae* | Diffusion disk; Nutrient agar | 24 h | Agastian et al., 2019 | India |

*Thermogravimetric analysis (TGA), Gas chromatography-mass spectrometry (GC-MS/MS) and High performance liquid chromatography/ultraviolet-visible (HPLC/UV-VIS), Electron diffraction (SAED), X-ray diffraction (XRD), Dynamic light scattering (DLS), Energy dispersive spectroscopy (EDX), Energy dispersive spectroscopy (EDS), Fourier transform infrared spectroscopy (FTIR), Raman spectroscopy (Raman), Ultraviolet-visible spectroscopy (UV-Vis), Field emission splitting electron microscopy (FE-SEM), Transmission electron microscopy (TEM), High resolution transmission electron microscopy (HRTEM), Scanning electron microscopy (SEM), Zeta Potential (Zeta).

Table 5. Studies on the synthesis of green gold nanoparticles from fruit extract, including plant species, synthesis methods, characterization techniques, applied concentrations, tested bacterial strains, exposure time, and authors.

| **Species** | **Synthesis Method** | **Methods Used for NP Characterization** | **Employed NP concentrations** | **Description of Bacteria Used** | **Experimental Design and Culture Medium** | **Exposure Time** | **References** | **Country** |
| --- | --- | --- | --- | --- | --- | --- | --- | --- |
| Pineapple *(Ananas Comosus (L.))* | 20 g of fruit ground in 100 mL of water and centrifuged at 10,000 rpm for 15 min; jackfruit extract at 50 % (v/v) added to a solution of AuCl₄ 2 mmol L⁻¹ in dark for 2 h at room temperature | UV-Vis, SEM, XRD, and FT-IR | 10 μL (100 mg mL⁻¹) | *Strains: Escherichia coli and* *Streptobacillus sp.; Fungi: Aspergillus niger, Aspergillus flavus* | Disk diffusion; Muller Hinton Agar (MHA) (Hi-Media) and fungal species on Potato Dextrose Agar (PDA) | 24 h (bacteria); 48 h (fungus) | Basavegowda et al., 2014 | India |
| Jackfruit *(Artocarpus heterophyllus Lam.)* | 20 g of fruit ground in 100 mL of water and centrifuged at 10,000 rpm for 15 min; jackfruit extract at 50 % (v/v) added to a solution of AuCl₄ 2 mmol L⁻¹ in dark for 2 h at room temperature | UV-Vis, SEM, and FT-IR | 1 mg mL⁻¹ of metanol | *Escherichia coli and Streptobacillus sp.* | Disk diffusion; Muller Hinton Agar | 24 h | Basavegowda et al., 2019 | India |
| Oriental Melon *(Cucumis melo L)* | 50 g in 250 mL of deionized water, boiled for 15 min; 10 mL extract and 100 mL HAuCl₄ at room temperature for 24 h | FE-SEM, TGA, UV-Vis, TEM, XRD, and FTIR | 50 μL (16.7 mg mL⁻¹) | *Bacillus cereus,* *Listeria monocytogenes, Staphylococcus aureus, Escherichia coli, Salmonella typhimurium* | Disk diffusion; Mueller-Hinton Agar | 48 h | Patra et al., 2016 | South Korea |
| Chinese Wolfberry *(Lycium chinense)* | 10 g of fine powder obtained using a grinding machine and boiled for 1 h in 100 mL sterile water at 100 °C and filtered; 5 mL of filtered L. chinense stock mixed with 50 mL HAuCl₄ at 80 °C, 15,000 rpm for 20 min and air dried | UV-Vis, SEM, XRD, DLS, and FT-IR | 100 mg L⁻¹ | *Escherichia coli and Staphylococcus aureus* | Disk diffusion; Muller Hinton Agar | 24 h | Chokkalingam et al., 2019 | South Korea |
| Chinese Hawthorn *(Crataegus pinnatifida)* | HAuCl₄ dissolved in 10 % (v/v) fruit aqueous extract with a final concentration of 1 mmol L⁻¹ at 80 °C in oil bath; 16,000 rpm for 15 min and air dried | FE-SEM, UV-Vis, TEM, SAED, and DLS | 1, 5, 10, 25, 50, 100 μg mL⁻¹ for MIC and 15, 30, 45 μg mL⁻¹ for Disk | *Escherichia coli and Staphylococcus aureus* | – | 24 h | Kang et al., 2017 | South Korea |
| Pumpkin *(Cucurbita moschata)* | 50 g of dried peel and 750 mL of distilled water (1:3 ratio); 250 mL of HAuCl₄·3H₂O in 750 mL of plant extract (1:3 ratio); 15 min at 50 °C | EDX, UV-Vis, TEM, SEM, XRD, and FTIR | 16.6 mg mL⁻¹ | *Bacteria: Escherichia coli and Staphylococcus aureus; Fungus: Candida albicans* | Minimum inhibitory concentration; Mueller-Hinton broth | 24 h | Kaval et al., 2024 | Turkey |
| Cranberry *(Vaccinium subg. Oxycoccus)* | 10 g chopped fruits with 20 mL distilled water up to 100 mL; fruit extract (1 mL) combined with various amounts of HAuCl₄·4H₂O (10 mmol L⁻¹) (1:0.25, 1:0.5, 1:1, 1:1.25, 1:2 mmol L⁻¹); temperatures: 20, 40, 60, 80, 100, 120 °C; time: 60–240 min | SEM, TEM, TGA, UV-Vis, XRD | 25, 50, 75, and 100 μg mL⁻¹; standard antibiotic azithromycin 30 μg mL⁻¹ | *Klebsiella pneumoniae, Pseudomonas aeruginosa, Bacillus subtilis, Enterococcus faecalis* | Disc diffusion; Mueller-Hinton agar | 24 h | Queen et al., 2025 | India |
| Bahera *(Terminalia bellerica)* | 2 g fruit with 20 mL bidistilled water; 1:1 ratio of aqueous extract and HAuCl₄·3H₂O (3 mmol L⁻¹) at 27 °C until color change | UV-Vis, FE-SEM, FT-IR, XRD | 100 μg mL⁻¹ | *Acinetobacter baumanii, Bacillus subtilis, Enterococcus faecalis* | Agar well diffusion; Mueller-Hinton | 24 h | Chithambharan et al., 2021 | India |
| Arjuna *(Terminalia arjuna)* | 10 g fresh fruits in 100 mL bidistilled water, boiled at 50–60 °C for 5 min and filtered; 1 mL extract in 100 mL HAuCl₄ solution (1 mmol L⁻¹) at room temperature for 15 min | UV-Vis, TEM, XRD, FT-IR, Zeta Potential, DLS | 500 µmol mL⁻¹ and 1000 µmol mL⁻¹ (100 μg mL⁻¹) | *Staphylococcus aureus, Klebsiella pneumoniae, Proteus vulgaris* | Disc diffusion; nutrient agar | 24 h | Gopinath et al., 2014 | India |
| Kiwi *(Actinidia deliciosa)* | 100 g peeled and ground fruit in 100 mL water, filtered; 1 mL fruit extract added to 9 mL HAuCl₄·3H₂O (1 mmol L⁻¹) | EDAX, EDX, XRD, EDAX, FT-IR, UV-Vis, TEM | 100, 200, 300 μg mL⁻¹ | *Pseudomonas aeruginosa* | Disc diffusion; Mueller-Hinton agar | 24 h | Naraginti and Li, 2017 | China |
| Elderberry *(Sambucus nigra L.)* | 2.5 % v/v in extract mixture; extract volume later doubled (5 % v/v) and tripled (7.5 % v/v); increasing volumes of HAuCl₄ solution (10 mmol L⁻¹) added again | UV-Vis, FTIR, DLS, TEM | — | *Staphylococcus aureus, Escherichia coli* | Minimum inhibitory concentration; Mueller-Hinton broth | 24 h | Mariychuk et al., 2020 | Slovakia |
| Privet *(Ligustrum vulgare)* | 10 g fresh berries boiled with 90 mL distilled water; extract volume in HAuCl₄·3H₂O to 1 mmol L⁻¹ at room temp until color change | UV-Vis, SEM, EDX, TEM, DLS, sp-ICP-MS, TGA, FT-IR, MALDI-TOF | 16, 32, 50, 100, 150, 200 μg mL⁻¹ | *Escherichia coli, Pseudomonas aeruginosa* | Disc diffusion; Mueller-Hinton agar | 24 h | Singh and Mijakovic, 2022 | Denmark |
| Parijoto *(Medinilla speciosa)* | 20 g fruit in 200 mL distilled water heated at 90 °C for 15 min; infusion with HAuCl₄·3H₂O (1 mmol L⁻¹) at v/v ratios 1:4 (F₁), 1:6 (F₂), 1:8 (F₃) | UV-Vis, PSA, FTIR, TEM | Extract: 100 mg mL⁻¹; AuNPs: 400, 600, 800 mg mL⁻¹ | *Pseudomonas aeruginosa, Staphylococcus aureus* | Agar well diffusion; Mueller-Hinton | 18–24 h | Prihapsara et al., 2022 | USA |
| Ginseng-berry *(Panax ginseng)* | 10 g powder in 150 mL sterile distilled water, autoclaved at 100 °C for 30 min; 100 mL extract to reach HAuCl₄ 1 mmol L⁻¹ at 23, 40, 60, 80, 90 °C | UV-Vis, FE-TEM, EDX, XRD, SAED, FT-IR | 15, 30, 45 mg mL⁻¹ | *Staphylococcus aureus, Escherichia coli* | Disc diffusion; Mueller-Hinton agar | 24 h | Jiménez Pérez et al., 2017 | South Korea |
| Citrus fruit flavonoids Hesperidin (HDN) and Naringin (NRG) | 4 mg mL⁻¹ and 8 mg mL⁻¹ respectively, stirred 24 h at room temp; 1 mL extract with 0.1 mL HAuCl₄ (5 mmol L⁻¹), magnetically stirred 4 h at 60 °C | UV-Vis, FT-IR, Zeta Potential, AFM | 50 μg mL⁻¹; gentamicin 100 μg mL⁻¹ | *Methicillin-resistant Staphylococcus aureus (MRSA), Escherichia coli K1* | Disc diffusion; nutrient agar | 24 h | Anwar et al., 2019 | Malaysia |
| Mistletoe *(Viscum album)* | 10 g fruit in 100 mL distilled water heated at 25 °C; infusion with HAuCl₄·3H₂O (1 mmol L⁻¹) | UV-Vis, FE-TEM, EDX, XRD | 50 and 100 μg L⁻¹ | *Enterobacter, Salmonella typhi, Escherichia coli, Bacillus subtilis* | Minimum inhibitory concentration; Mueller-Hinton broth | 24 h | Ishaq et al., 2023 | Pakistan |
| Arak tree *(Salvadora persica)* | 10 g dried fruit in 100 mL sterile water; 10 mL extract (10 % w/v) in 40 mL HAuCl₄ (0.1 mmol L⁻¹), stirred in dark at room temp 24 h | Extract: GC-MS; NPs: UV-Vis, EDX, TEM, FTIR, Zeta Potential | 100 μg mL⁻¹; standard antibiotic Gentamicin 4 μg mL⁻¹ | *MRSA Staphylococcus aureus, Escherichia coli, Porphyromonas gingivalis* | Agar well diffusion; nutrient agar | 24 h | Elhabal et al., 2022 | Egypt |
| Chinese Quince *(Chaenomeles sinensis)* | 10 g fine powder of dried fruit in 100 mL distilled water; HAuCl₄·3H₂O (1 mmol L⁻¹) added to diluted extract (70 %) at room temp for 10 s | UV-Vis, FE-TEM, XRD | 100 mg L⁻¹; neomycin | *Staphylococcus aureus, Escherichia coli* | Disc diffusion; Mueller-Hinton agar | 24 h | Oh et al., 2018 | South Korea |
| Tamarind *(Tamarindus indica)* | 5 g dry seeds in 100 mL double⁻deionized water, stirred up to 24 h at 80 °C; 5 mL HAuCl₄ (1 mmol L⁻¹) with 1 mL extract solution at 25 °C until color change | UV-Vis, FT-IR, AFM | 1 mg mL⁻¹; standard antibiotic streptomycin 2 mg mL⁻¹ | *Klebsiella pneumoniae, Bacillus subtilis, Staphylococcus epidermidis* | Disc diffusion; Mueller-Hinton agar | 24 h | Ullah et al., 2021 | Pakistan |

*Thermogravimetric analysis (TGA), Gas chromatography-mass spectrometry (GC-MS/MS) and High performance liquid chromatography/ultraviolet-visible (HPLC/UV-VIS), Electron diffraction (SAED), X-ray diffraction (XRD), Dynamic light scattering (DLS), Energy dispersive spectroscopy (EDX), Energy dispersive spectroscopy (EDS), Fourier transform infrared spectroscopy (FTIR), Raman spectroscopy (Raman), Ultraviolet-visible spectroscopy (UV-Vis), Field emission splitting electron microscopy (FE-SEM), Transmission electron microscopy (TEM), High resolution transmission electron microscopy (HRTEM), Scanning electron microscopy (SEM), Zeta Potential (Zeta).

Table 6. studies on the synthesis of green gold nanoparticles from stem extract, including plant species, synthesis methods, characterization techniques, applied concentrations, tested bacterial strains, exposure time, and authors.

| **Species** | **Synthesis Method** | **Methods Used for NP Characterization** | **Employed NP concentrations** | **Description of Bacteria Used** | **Experimental Design and Culture Medium** | **Exposure Time** | **References** | **Country** |
| --- | --- | --- | --- | --- | --- | --- | --- | --- |
| Dragon’s blood *(Croton draco)* | Aqueous stem extract (1:2) HAuCl₄ 1 mmol L⁻¹ | UV-Vis, XRD, TEM, SEM, FTIR | 1 mg mL⁻¹ | *Staphylococcus aureus and Pseudomonas aeruginosa* | Agar well diffusion method on Mueller-Hinton | 24 h | Elizalde-mata et al., 2025 | Mexico |
| Horsetail *(Equisetum diffusum)* | 10 g of powdered material in 200 mL deionized water, stirred at room temperature for 5 h and filtered; 1 mmol of HAuCl₄ prepared by dissolving 33.97 mg in 100 mL DH₂O; extract:salt ratio (1:4) reacted for 10 min at 60 °C | UV-Vis, TEM, SEM, FTIR, and DLS | 20, 30, and 40 μg mL⁻¹ and 30 μg mL⁻¹ ciprofloxacin; 80 for MIC μg mL⁻¹ | *Staphylococcus epidermidis, Listeria monocytogenes, Staphylococcus aureus, Pseudomonas aeruginosa,* *Bordetella bronchiseptica, and Escherichia coli* | Agar well diffusion method on Mueller-Hinton; Minimum Inhibitory Concentration (MIC) in Mueller-Hinton broth | 24 h | Assad et al., 2025 | Ethiopia |
| Azanza lampas *(Thespesia lampas)* | 10 g of shade⁻dried stem powder extracted in 100 mL water; 10 mL of extract (5 %, pH 6) with 90 mL HAuCl₄ 2 mmol L⁻¹ at 400 rpm for 24 h at room temperature | UV-Vis, XRD, SEM-EDS, HR-TEM, SAED, and FTIR | 0.5 to 50 μg mL⁻¹ | *Escherichia coli, Bacillus subtilis, Proteus vulgaris, and Salmonella typhi* | Agar well diffusion method on Mueller-Hinton | 24 h | Nath et al., 2024 | India |
| White parthenium *(Parthenium hysterophorus)* | 10 g of powdered material with 100 mL bidistilled water; 10 mL extract with 90 mL salt at 70 °C for 60 min. Controlled: reaction time (15, 30, 45, 60, 100 min), metal ion concentration (0.5–5 mmol L⁻¹), pH (4, 6, 8, 10, 11), extract concentration (5–25 %), reaction temperature (25–80 °C); optimized: 10 % extract, pH 6, 1 mmol L, 40 °C, 60 min | UV-Vis, XRD, DLS, and Zeta Potential | 1 mg mL⁻¹ | *Enterococcus faecalis,* *Salmonella enterica, Escherichia coli, Staphylococcus aureus* | Agar well diffusion method on Mueller-Hinton | 24 h | Leyu et al., 2023 | Ethiopia |
| Ababangai *(Oroxylum indicum)* | 30 mg plant extract with 30 mL HAuCl₄ 1 mmol L⁻¹ in round⁻bottom flask in microwave oven (1,100 W) irradiated for 3 min at 800 W | UV-Vis, FTIR, X-ray, TG | 0.25, 0.5, and 1 mg mL⁻¹ | *Escherichia coli, Staphylococcus aureus* | Diffusion disk; Mueller-Hinton agar | - | Worakitjaroenphon et al., 2023 | Thailand |
| Trapoeraba *(Commelina nudiflora)* | 10 g in 100 mL deionized water, heated at 60 °C for 15 min; 10 mL plant broth added with 190 mL aqueous HAuCl₄ (1 mmol L⁻¹), stirred at 37 °C, 150 rpm for 3 h | UV-Vis, FTIR, FE-SEM, XRD | 100 μg mL⁻¹ and streptomycin 10 μg mL⁻¹ | *Escherichia coli, Staphylococcus aureus, Salmonella typhi, Enterococcus faecalis* | - | - | Kuppusamy et al., 2015b | Malaysia |
| Periploca *(Periploca hydaspidis)* | 1:8 ratio of AuCl₃ 1 mmol L⁻¹ with boiled plant extract until color change | UV-Vis, FE-SEM, XRD, FTIR | 125 μg mL⁻¹ | *Escherichia coli, Klebsiella pneumoniae, Xanthomonas compestris, Candida albicans,* *Penicillium chrysogenum* | Diffusion disk; Mueller-Hinton agar | 24 h | Das and Ullah et al., 2020 | Pakistan |
| Eucalypto *(Eucalyptus sp.)* | 2 g of powder in 100 mL of Millipore water under stirring until boiling for 10–15 min and filtered In. 30 mL of HAuCl₄ (1.0575 mmol L⁻¹) placed in 12 different flasks with **2** to 4 mL of extract, temperatures of 30, **40** and 50 °C. | UV-Vis, FE-SEM, XRD, FTIR, AFM | 20, 40 e 60 μL (26, 52 e 78 mg mL⁻¹) | *Staphylococcus sp., Pseudomonas sp., Bacillus sp., Escherichia coli* | Diffusion disk; Mueller-Hinton agar | 24 h | Muthiah et al., 2020 | India |
| Salicornia *(Salicornia brachiata)* | 50 mg lyophilized aqueous extract in 50 mL Milli⁻Q water, filtered. 1 mL HAuCl₄ (1 mmol L⁻¹) added to 50 mL extract, stirred at 60 °C until color change | UV-Vis, FE-SEM, XRD, SEM, TEM | 0.5 mg mL⁻¹ | *Pseudomonas aeruginosa, Salmonella typhi, Escherichia coli, Staphylococcus aureus* | Diffusion disk; Mueller-Hinton agar | 24 h | Ayaz Ahmed et al., 2014 | India |
| Oal *(Amorphophallus paeoniifolius)* | 0.1 g stem powder in 100 mL distilled water for 90 min to form extract. For AuNP synthesis, 100 µL 0.8 mmol L HAuCl₄ in 400 µL extract at room temperature until color change | UV-Vis, FE-SEM, XRD, SEM, FTIR, EDS | 0.8 mg mL⁻¹ | *Escherichia coli,* *Citrobacter freundii, Bacillus subtilis, Pseudomonas aeruginosa, Salmonella typhimurium, Staphylococcus aureus* | Diffusion disk; Mueller-Hinton agar | 48 h | Nayem et al., 2020 | Bangladesh |

* Thermogravimetric analysis (TGA), Gas chromatography-mass spectrometry (GC-MS/MS) and High performance liquid chromatography/ultraviolet-visible (HPLC/UV-VIS), Electron diffraction (SAED), X-ray diffraction (XRD), Dynamic light scattering (DLS), Energy dispersive spectroscopy (EDX), Energy dispersive spectroscopy (EDS), Fourier transform infrared spectroscopy (FTIR), Raman spectroscopy (Raman), Ultraviolet-visible spectroscopy (UV-Vis), Field emission splitting electron microscopy (FE-SEM), Transmission electron microscopy (TEM), High resolution transmission electron microscopy (HRTEM), Scanning electron microscopy (SEM), Zeta Potential (Zeta).

Table 7. Studies on the synthesis of green gold nanoparticles from aquatic plants extract, including plant species, synthesis methods, characterization techniques, applied concentrations, tested bacterial strains, exposure time, and authors.

| **Species** | **Synthesis Method** | **Methods Used for NP Characterization** | **Employed NP concentrations** | **Description of Bacteria Used** | **Experimental Design and Culture Medium** | **Exposure Time** | **References** | **Country** |
| --- | --- | --- | --- | --- | --- | --- | --- | --- |
| Brown algae *(Sargassum plagiophyllum)* | 10 g of dried powder with 100 mL of distilled water; 100–1000 μL of extract were added to 5 mL of HAuCl₄·xH₂O solution (1 mmol L⁻¹) at 60 °C until color change | FE-SEM, SEM, AFM, UV-Vis | 10 μg mL⁻¹ | *Escherichia coli and Salmonella typhi* | Agar well diffusion method; Mueller-Hinton agar | 24 h | Dhas et al., 2020 | India |
| Spiny algae *(Acanthophora spicifera)* | 2.5 g of dried powder in 250 mL Milli⁻Q water at 60 °C for 20 min; 65 µL of HAuCl₄ 1 mol L⁻¹ solution gradually added to 250 mL aqueous extract under stirring at 60 °C for 4 h | UV-Vis, TEM, SEM, XRD, Zeta Potential, FTIR, and EDAX | 25, 50, 75, and 100 μg mL⁻¹ | *Vibrio harveyi and Staphylococcus aureus* | Agar well diffusion method; Mueller-Hinton agar | 24 h | Babu et al., 2020 | India |
| Sea grape *(Caulerpa racemosa)* | 2 g of seeds boiled in 100 mL deionized water for 5 min; 1 mL extract added to 30 mL HAuCl₄ (2.5×10⁻⁴ mol L⁻¹) at boiling (373 K) for 2 min. Experiment repeated with 5, 10, 15, and 20 mL extract volumes to obtain colloids B₂, B₃, B₄, and B₅, respectively | UV-Vis, TEM, SEM, XRD, Zeta Potential, FTIR, and EDAX | 25, 50, 75, and 100 μg mL⁻¹ | *Aeromonas veronii and Streptococcus agalactiae* | Agar well diffusion method; Mueller-Hinton agar | 24 h | Manikandakrishnan et al., 2019 | India |
| Harvey *(Undaria pinnatifida)* | 1 g mL⁻¹ extract with slow addition of 0.4 mmol L⁻¹ HAuCl₄ at room temperature for 24 h | UV-Vis, XRD, Zeta Potential, FTIR, and FESEM | 0.54 and 11.81 μg mL⁻¹ for AuNPs; 5 and 60 μg mL⁻¹ for kanamycin and ampicillin | *Bacteria: Escherichia coli, Staphylococcus aureus, Pseudomonas aeruginosa; Fungi: Candida albicans and* *Candida auris* | Minimum Inhibitory Concentration (MIC); Mueller-Hinton broth | Overnight | González-ballesteros et al., 2023 | Portugal |
| Sargassum algae (*Sargassum incisifolium*) | 2 mg of lyophilized extract dissolved in 10 mL distilled water and stirred for 10 min; 500 µL of HAuCl₄ (0.1 mol L⁻¹) added to 10 mL and reaction continued for 18 h at room temperature | UV-Vis, SEM, XRD, and Zeta Potential | 0.4 mg mL⁻¹; 1 mg mL⁻¹ of vancomycin, ampicillin, and chloramphenicol | *Bacteria: Vancomycin-resistant Enterococcus faecalis, MRSA Staphylococcus aureus, Acinetobacter baumannii, and β-lactam-resistant Klebsiella pneumoniae; Fungus: Candida albicans* | Agar well diffusion method; Nutrient agar | 24 h | Mmola et al., 2016 | South Africa |

*Thermogravimetric analysis (TGA), Gas chromatography-mass spectrometry (GC-MS/MS) and High performance liquid chromatography/ultraviolet-visible (HPLC/UV-VIS), Electron diffraction (SAED), X-ray diffraction (XRD), Dynamic light scattering (DLS), Energy dispersive spectroscopy (EDX), Energy dispersive spectroscopy (EDS), Fourier transform infrared spectroscopy (FTIR), Raman spectroscopy (Raman), Ultraviolet-visible spectroscopy (UV-Vis), Field emission splitting electron microscopy (FE-SEM), Transmission electron microscopy (TEM), High resolution transmission electron microscopy (HRTEM), Scanning electron microscopy (SEM), Zeta Potential (Zeta).

Table 8. Studies on the synthesis of green gold nanoparticles from bark/peel extract, including plant species, synthesis methods, characterization techniques, applied concentrations, tested bacterial strains, exposure time, and authors.

| **Species** | **Synthesis Method** | **Methods Used for NP Characterization** | **Employed NP concentrations** | **Description of Bacteria Used** | **Experimental Design and Culture Medium** | **Exposure Time** | **References** | **País** |
| --- | --- | --- | --- | --- | --- | --- | --- | --- |
| Watermelon *(Citrullis lanatus var)* | 100 g of each part (red and green) in 50 mL of Milli⁻Q® water for 10 min, 45 s microwave heating (1100 W at 2450 Hz) and filtered; 1, 3, and 5 mL to 1 mL of AuCl₄⁻ (500 ppm) for 2 h at room temperature | UV-Vis, XRD, SEM, and EDX | 50 μL (2 g mL⁻¹) | *Escherichia coli and Staphylococcus epidermidis* | Diffusion disk; Nutrient agar | 48 h | Chumsa-ard et al., 2019 | Australia |
| Pomegranate *(Punica granatum)* | Aqueous extract of peel (1:100); 5 min; 56 °C | UV-Vis, XRD, TEM, FT-IR, and Raman | 1 mg of coconut fiber in 43, 128, and 256 μg mL⁻¹ of AuNPs | *Staphylococcus aureus, Enterococcus faecalis, Escherichia coli, and Pseudomonas aeruginosa* | Minimum Inhibitory Concentration (MIC); Nutrient broth | 10 h under agitation at 120 rpm | Silva et al., 2023 | Brazil |
| Langsat *(Lansium domesticum)* | 10 g of fruit peels in 100 mL bidistilled water at 80 °C for 12 h; 2 mL extract in 18 mL HAuCl₄ (0.5 mmol L⁻¹) at room temperature until color change | UV-Vis, XRD, SEM, DLS, and FT-IR | 32 μg mL⁻¹ and 16 μg mL⁻¹ | *Staphylococcus aureus and Escherichia coli* | Diffusion disk; Nutrient agar | 24 h | Shankar et al., 2014 | Australia |
| Macadamia *(Macadamia integrifolia)* | 10 g in 100 mL deionized water for 2 h at 95 °C; aqueous peel extract (1.5, 10, 15, 20, 25 mL in 1 mL HAuCl₄ 1 mmol L⁻¹); 22 h | UV-Vis, XRD, SEM, and EDS | 50 μL (1.0, 1.5, 2.0, 2,5 mg m L⁻¹) | *Escherichia coli and Staphylococcus epidermidis* | Diffusion disk; Mueller Hinton agar | 24 h | Dang et al., 2018 | Australia |
| Cannabis *(Cannabis sativa)* | 150 g in 600 mL of 40 % ethanol solution. Constant extract concentration of 10 % and metal ion concentrations varying from 0.5 to 5.0 mmol L⁻¹ at room temperature until color change | UV-Vis, XRD, SEM, and FT-IR | 10% v/v of Cannabis sativa extract with 2 mmol L⁻¹ tetrachloroauric acid solution (for AuNPs). 1.2, 2.4, 4.8, 7.2, 9.5, 11.9 mg L⁻¹ | *Pseudomonas aeruginosa* | Luria broth (LB) | 24 h | Michailidu et al., 2025 | Czech Republic |
| Cowpea *(Bauhinia variegata)* | 0.1 g mL⁻¹ extract; 30 mL of 1 mmol L⁻¹ HAuCl₄ solution, 20 mL peel extract, stirred for 35 min on magnetic heating plate at 70 °C | UV-Vis, XRD, EDS, and FT-IR | 50 μL (66 mg mL⁻¹) | *Bacillus subtilis and Escherichia coli* | Well diffusion method on Mueller-Hinton agar | 24 h | Vaghela; Parmar; Mahyavanshi, 2023 | India |
| Neem *(Nilavembu Choornam)* | 7.5 g L⁻¹ filtered extract; 10 mL of extract with 40 mL HAuCl₄ solution (0.1 mmol L⁻¹) at 37 °C until color change; 10,000 × g for 10 min, air⁻dried | UV-Vis, XRD, EDS, SEM, and FT-IR | 0.25; 0.5; 1 mg mL⁻¹ | *Klebsiella pneumoniae, Staphylococcus aureus, Pseudomonas aeruginosa, and Enterococcus faecalis* | Well diffusion method on Mueller-Hinton agar | 24 h | Almutleb et al., 2024 | Saudi Arabia |
| Yerba mansa *(Anemopsis californica)* | 5 g biomass in 100 mL boiling water for 2 min; three extracts (water, methanol, isopropanol) in 2:5:3 volume ratio (extract : HAuCl₄ 1 mmol L⁻¹: deionized water) at room temperature until color change | UV-Vis and TEM | 0.8; 1.6; 3.2; 6.4; 13; 26; 39 μg mL⁻¹ | *Staphylococcus aureus and Escherichia coli* | Minimum Inhibitory Concentration (MIC); Nutrient broth | 24 h | Avilés et al., 2024 | Mexico |
| Onion *(Allium cepa L.)* | 100 g dried peel in 200 mL distilled water (1:2) at 85 °C for 10 min; 50 mL extract in 300 mL HAuCl₄·3H₂O 75 mmol L⁻¹ (1:6) at room temp, 20 min (10,000 rpm), dried at 80 °C for 48 h | UV-Vis, TEM, XRD, FT-IR | 16 μg mL⁻¹ | *Bacteria: Staphylococcus aureus, Bacillus subtilis, Escherichia coli, Pseudomonas aeruginosa; Fungus: Candida albicans* | Minimum Inhibitory Concentration (MIC); Mueller-Hinton broth | 24 h | İpek et al., 2024 | Turkey |
| Red sandalwood *(Pterocarpus santalinus L.)* | 5 g peel in 100 mL water; 1 mL extract to 4 mL of different salt concentrations (0.3, 0.5, 1, 1.5, 2 mmol L⁻¹), temperatures (28, 35, 50, 75, 100 °C) under physiological conditions (pH 7.4) | UV-Vis, XRD, FTIR, and TEM | 50 μg mL⁻¹; Chloramphenicol 1 mg mL⁻¹ | *Staphylococcus aureus and Pseudomonas aeruginosa* | Diffusion disk; Mueller Hinton agar | 24 h | Keshavamurthy; Srinath; Rai, 2018 | India |
| Pomelo *(Citrus maxima)* | 1 g peel in 100 mL deionized water, boiled 10 min. 100 μL chloroauric acid (100 mmol L⁻¹) in 10 mL extract, stirred at room temperature until color change | XRD, TEM, FT-IR | 1.0, 1.5, 2.0 mmol L⁻¹ (1 g mL⁻¹) | *Staphylococcus aureus and Escherichia coli* | Minimum Inhibitory Concentration (MIC); Nutrient broth | 24 h | Yuan et al., 2017 | China |
| Curry *(Murraya koenigii)* | 3 mL containing various doses of aqueous peel extract (0.4, 0.6, 0.8, 1 mL) and 1 mmol L⁻¹ HAuCl₄ in PBS (50 mmol L⁻¹, pH 7.4) at 40 °C for 24 h | FT-IR, TGA, UV-Vis, TEM, and zeta potential | 5.6, 9.9, 3.4, 12.23 μg mL⁻¹ | *Staphylococcus aureus, Pseudomonas aeruginosa, Klebsiella pneumoniae, and Escherichia coli* | Minimum Inhibitory Concentration (MIC); Nutrient broth | 24 h | Mishra et al., 2023 | India |
| Red Propolis *(Dalbergia ecastophyllum)* | HAuCl₄·3H₂O (0.5 mmol L⁻¹) with extract solution (200 μg mL⁻¹) and its fractions (hexane, acetate, dichloromethane), then NaOH added to pH 7.0, stirred 1 h at room temperature | SAED, FTIR, TGA, UV-Vis, TEM, and EDX | 200 μg mL⁻¹ | *Staphylococcus aureus, Escherichia coli, Streptococcus mutans, and Candida albicans* | Minimum Inhibitory Concentration (MIC); Nutrient broth | 24 h | Botteon et al., 2021 | Brazil |
| Patala *(Stereospermum chelonoides)* | 5 g root peel in 100 mL bidistilled water at 60 °C for 20 min, filtered; 90 mL HAuCl₄·3H₂O (1 mmol L⁻¹) in 10 mL extract in domestic microwave for 1 min | SAED, FTIR, TGA, UV-Vis, AFM | 1 mg mL⁻¹ | *Bacteria: Bacillus subtilis, Staphylococcus aureus, Escherichia coli, Pseudomonas aeruginosa; Fungi: Aspergillus nidulans, Aspergillus flavus* | Well diffusion method on Mueller-Hinton agar | considerable time | Francis; Koshy; Mathew, 2018 | India |
| Banana *(Musa paradisiaca)* | 1 g in 10 mL distilled water at 90 °C; 2 mL peel extract in 25 mL HAuCl₄ 1 mmol L⁻¹ at 353 K for 20 min | UV-Vis, XRD, FT-IR, TEM, EDX, and zeta potential | 25, 50, 100 μg mL⁻¹ | *Resistant Enterococcus faecalis* | Antibiofilm activity | considerable time | Vaseeharan et al., 2017 | India |

*Thermogravimetric analysis (TGA), Gas chromatography-mass spectrometry (GC-MS/MS) and High performance liquid chromatography/ultraviolet-visible (HPLC/UV-VIS), Electron diffraction (SAED), X-ray diffraction (XRD), Dynamic light scattering (DLS), Energy dispersive spectroscopy (EDX), Energy dispersive spectroscopy (EDS), Fourier transform infrared spectroscopy (FTIR), Raman spectroscopy (Raman), Ultraviolet-visible spectroscopy (UV-Vis), Field emission splitting electron microscopy (FE-SEM), Transmission electron microscopy (TEM), High resolution transmission electron microscopy (HRTEM), Scanning electron microscopy (SEM), Zeta Potential (Zeta).

Table 9. Studies on the synthesis of green gold nanoparticles from sap extract, including plant species, synthesis methods, characterization techniques, applied concentrations, tested bacterial strains, exposure time, and authors. Source: The author (2025)

| **Species** | **Synthesis Method** | **Methods Used for NP Characterization** | **Employed NP concentrations** | **Description of Bacteria Used** | **Experimental Design and Culture Medium** | **Exposure Time** | **References** | | **Country** | |
| --- | --- | --- | --- | --- | --- | --- | --- | --- | --- | --- |
| Bicuíba *(Virola oleifera)* | Aqueous extract of the sap 1 mg mL⁻¹, volumes: 1 mL, 2 mL, and 3 mL in 10 mL of acid | Factorial design with variables: extract solution volume, stirring, and time. UV-Vis, TEM, XRD, Zeta Potential, FT-IR, and Raman | 1 mg mL⁻¹ of extract: 1 mL, 2 mL, and 3 mL in 10 mL of acid | *Staphylococcus aureus and Escherichia coli* | Minimum Inhibitory Concentration (MIC); Mueller Hinton broth | 24 h, 48 h, and 72 h | Millaneze et al., 2015 | Brazil | |  |
| Bicuíba *(Virola oleifera)* | 1 mg of dried sap in 1 mL distilled water at room temperature until dissolved and then filtered; Aqueous sap extract and HAuCl₄ (2.5 × 10⁻⁴ mol L⁻¹) in a 3:7 ratio at 400 rpm, 6 min, 25°C, pH 8.3 | Factorial design with variables: extract solution volume, stirring, and time. UV-Vis, TEM, XRD, Zeta Potential, FT-IR, and Raman | 0.019 mg mL⁻¹ or 9.9912 × 10⁻⁵ mmol mL⁻¹ | *Staphylococcus aureus and Pseudomonas aeruginosa* | Minimum Inhibitory Concentration (MIC); Mueller Hinton broth | 24 h, 48 h, and 72 h | Marques et al., 2023 | Brazil | |  |

*Thermogravimetric analysis (TGA), Gas chromatography-mass spectrometry (GC-MS/MS) and High performance liquid chromatography/ultraviolet-visible (HPLC/UV-VIS), Electron diffraction (SAED), X-ray diffraction (XRD), Dynamic light scattering (DLS), Energy dispersive spectroscopy (EDX), Energy dispersive spectroscopy (EDS), Fourier transform infrared spectroscopy (FTIR), Raman spectroscopy (Raman), Ultraviolet-visible spectroscopy (UV-Vis), Field emission splitting electron microscopy (FE-SEM), Transmission electron microscopy (TEM), High resolution transmission electron microscopy (HRTEM), Scanning electron microscopy (SEM), Zeta Potential (Zeta).

Table 10. Studies on the synthesis of green gold nanoparticles from various extracts, including plant species, synthesis methods, characterization techniques, applied concentrations, tested bacterial strains, exposure time, and authors.

| **Species** | **Synthesis Method** | **Methods Used for NP Characterization** | **Employed NP concentrations** | **Bacteria Described** | **Experimental Design and Culture Medium Used** | **Exposure Time** | **References** | **Country** |
| --- | --- | --- | --- | --- | --- | --- | --- | --- |
| Marjoram (*Origanum majorana*) | 1 kg of powder macerated with 80 % MeOH at room temperature and then concentrated under reduced pressure using a rotary evaporator to a syrupy consistency. The concentrated methanolic extract yielded 60 g, and the dried extract was stored at 4 °C for in vitro and metabolomic studies. | UV-Vis, TEM, SEM, FT-IR | 250 μg mL⁻¹ | *Strains: Bacillus subtilis,* *Bacillus megaterium, Escherichia coli, and Proteus vulgaris; Fungi: Aspergillus niger, Fusarium solani, Candida albicans, and Aspergillus parasiticus* | Luria-Bertani broth (LB broth) | 24 h | El Ghorab et al., 2022 | Saudi Arabia |
| Olive (*Olea europaea*) and Egyptian Acacia (*Acacia nilotica*) | 10 g of fruit in 100 mL distilled water; mixture of olive fruit extract and acacia bark extract in a 3:5 ratio; 5 mL of extract to 50 mL of 1 mmol mL⁻¹ HAuCl₄ | UV-Vis, TEM, SEM, FT-IR | 10 mg mL⁻¹ | *Escherichia coli, Pseudomonas aeruginosa, and Klebsiella pneumoniae* | Diffusion disc; Mueller-Hinton Agar | 24 h | Awad et al., 2019 | Saudi Arabia |
| Eucalyptus and Jaborandi (*Eucalyptus sp. and Piper Longum*) | 10 g of bark from each plant in 1 L water. 1:16 (v/v) extract : HAuCl₄ 1 mol L⁻¹ at room temperature until color change | Not reported | 0.625 mg mL⁻¹ | *Bacteria: Staphylococcus aureus, Streptococcus mutans, E. faecalis; Fungus: Candida albicans* | Diffusion disc; Mueller-Hinton Agar | 24 h | Faiz; Sivaswamy; Rohinikumar, 2024 | India |
| Pomelo (*Citrus maxima*) | 10 g of fruits, leaves, and dried peels crushed in 100 mL Milli⁻Q water, heated in an oil bath at 80 °C for 2 h in round⁻bottom flasks and filtered. 20 mL of extracts dropwise into 200 mL of 1 mmol L⁻¹ HAuCl₄ under constant stirring at room temperature until color change | UV-Vis, TEM, SEM, DLS | 9.5 μg mL⁻¹, MIC Gentamicin (0.01 mg mL⁻¹) | *Resistant Pseudomonas aeruginosa* | Well diffusion method; Mueller-Hinton Agar; Minimum Inhibitory Concentration (MIC); Mueller Hinton Broth | 24 h | Jha et al., 2025 | India |
| Physalis (*Physalis minima*) | 20 g of whole plant extract in 100 mL water; three different concentrations of 0.1 mmol L⁻¹ gold chloride solution and aqueous extract: 1:10, 1:5, and 1:3 at room temperature until color change | UV-Vis, TEM, SEM, FT-IR, XRD, EDX, and DLS | 200 mg mL⁻¹ | *Staphylococcus aureus, Pseudomonas aeruginosa, Streptococcus pneumoniae, and Escherichia coli* | Well diffusion method; Mueller-Hinton Agar; amoxicillin | 24 h | Nagaraj et al., 2022 | India |
| Rooibos (*Aspalathus linearis (Burm.f.)*) | 1 mL (18 mg mL⁻¹) of extract prepared in ethanol added to 17 mL of salt and heated to 45 °C until color change | UV-Vis, XRD, FT-IR, TGA | 2 mg mL⁻¹; standard antibiotic tetracycline 0.2 mg mL⁻¹ | *Cutibacterium acnes* | Luria-Bertani broth (LB broth) | 72 h | Staden et al., 2021 | South Africa |
| Flavonoids: chrysin, kaempferol, and quercetin | 5 mL of aqueous solution of tetrachloroauric acid (0.025 mol L⁻¹) in 50 mL of 0.019 mol L⁻¹ aqueous solution of reduced L⁻glutathione (GSH), vigorously stirred for 30 min. Then conjugated with chrysin, kaempferol, and quercetin | UV-Vis, TEM, FT-IR, XRD, EDX, DLS | Final mixture concentrations: 960, 480, 240, 120, 60, 30, 15, 7.5, 3.25, 1.62, and 0.81 μg mL⁻¹ | *Pseudomonas aeruginosa, Escherichia coli, Proteus vulgaris, and Klebsiella pneumoniae* | Luria-Bertani broth (LB broth) | 24 h | Alhadrami et al., 2021 | Saudi Arabia |
| White poplar (*Populus alba*), Lantana (*Lantana camara*), and Hibiscus (*Hibiscus arboreus*) | Extracts prepared in 5, 10, and 15 % (v/v), boiled and filtered. HAuCl₄.3H₂O (1 mmol L⁻¹) and plant extracts in 9:1 (v/v) ratio, constant stirring in the dark for 7 min at room temperature | UV-Vis, TEM, SEM, DLS, FT-IR | 0, 10, 20, 30, 40, 50, 100 μg mL⁻¹ | *Staphylococcus aureus and Escherichia coli* | Diffusion disc; Mueller-Hinton Agar | 24 h | Acharya et al., 2021 | India |
| African Geranium (*Galenia africana*) and African Potato (*Hypoxis hemerocallidea*) | 50 mL boiled distilled water added to 5 g dried plant powder, centrifuged 2 h at 3750 rpm; 250 μL HAuCl₄.2H₂O 1.0 mmol L⁻¹ in 50 μL plant extract in 96⁻well plate (8.0 to 0.125 mg 300 μL⁻¹). Plate incubated 1 h at 70 °C, 40 rpm | UV-Vis, TEM, SEM, DLS, FTIR, HRTEM, TGA | 8.0 to 0.125 mg/300 μL; ampicillin as standard | *Pseudomonas aeruginosa, Staphylococcus aureus, Staphylococcus epidermidis, and Escherichia coli* | Nutrient broth with Alamar Blue | 24 h | Elbagory et al., 2017 | South Africa |
| Jabuticaba (*Plinia cauliflora*) and Pomegranate (*Punica granatum*) | 2.2 g leaves and fruits boiled at 80 °C in 40 mL bidistilled water. Solutions exposed to 300 W Cermax Xenon lamp for 1 min for photoreduction and pH adjusted to neutral. Then 25 mmol L HAuCl₄ added until color change | UV-Vis, FTIR, SEM, Zeta Potential | 55 mg mL⁻¹ | *Bacteria: Staphylococcus aureus, Bacillus subtilis, MRSA, Enterococcus faecalis, Escherichia coli (clinical isolate), Klebsiella pneumoniae, Salmonella Thiphymurium, Pseudomonas aeruginosa; Fungus: Candida albicans* | Minimum inhibitory concentration; Mueller-Hinton Broth | 20 h | Franzolim et al., 2022 | Brazil |
| Isolated β-caryophyllene | 200 mL deionized water, dissolving 1 mmol L⁻¹ HAuCl₄.3H₂O (pH 9.0), stirred at 60 °C. Dropwise solution of β-caryophyllene (1 mmol L⁻¹) added and stirred continuously at 60 °C | UV-Vis, FTIR, SEM, FETEM, XRD, EDX, Zeta Potential | 128–2048 μg mL⁻¹(MIC); 64–256 μg mL⁻¹(antibiofilm); standard antibiotics and antifungals tetracycline and fluconazole | *Fungus: Candida albicans; Bacteria: Staphylococcus aureus* | Minimum inhibitory concentration; Mueller-Hinton Broth | 24 h | Khan et al., 2023 | South Korea |
| Guduchi (*Tinospora cordifolia*) | 100 μL HAuCl₄ solutions to 100 mg isolated plant extracts at room temperature until color change | SEM, EDX | 1 g mL⁻¹ | *Pseudomonas aeruginosa* | Well diffusion method; Mueller-Hinton Agar | 24 h | Nath et al., 2023 | India |

*Thermogravimetric analysis (TGA), Gas chromatography-mass spectrometry (GC-MS/MS) and High performance liquid chromatography/ultraviolet-visible (HPLC/UV-VIS), Electron diffraction (SAED), X-ray diffraction (XRD), Dynamic light scattering (DLS), Energy dispersive spectroscopy (EDX), Energy dispersive spectroscopy (EDS), Fourier transform infrared spectroscopy (FTIR), Raman spectroscopy (Raman), Ultraviolet-visible spectroscopy (UV-Vis), Field emission splitting electron microscopy (FE-SEM), Transmission electron microscopy (TEM), High resolution transmission electron microscopy (HRTEM), Scanning electron microscopy (SEM), Zeta Potential (Zeta).
